# The Bilirubin Albumin Ratio in the Management of Hyperbilirubinemia in Preterm Infants to Improve Neurodevelopmental Outcome: A Randomized Controlled Trial – BARTrial

**DOI:** 10.1371/journal.pone.0099466

**Published:** 2014-06-13

**Authors:** Christian V. Hulzebos, Peter H. Dijk, Deirdre E. van Imhoff, Arend F. Bos, Enrico Lopriore, Martin Offringa, Selma A. J. Ruiter, Koen N. J. A. van Braeckel, Paul F. M. Krabbe, Elise H. Quik, Letty van Toledo-Eppinga, Debbie H. G. M. Nuytemans, Aleid G. van Wassenaer-Leemhuis, Manon J. N. Benders, Karen K. M. Korbeeck-van Hof, Richard A. van Lingen, Liesbeth J. M. Groot Jebbink, Djien Liem, Petri Mansvelt, Jan Buijs, Paul Govaert, Ineke van Vliet, Twan L. M. Mulder, Cecile Wolfs, Willem P. F. Fetter, Celeste Laarman, together with the BARTrial Study Group

**Affiliations:** 1 Department of Neonatology, Beatrix Children's Hospital, University Medical Center Groningen, Groningen, The Netherlands; 2 Division of Neonatology, Department of Pediatrics, Leiden University Medical Center, Leiden, The Netherlands; 3 Child Health Evaluative Sciences, The Hospital for Sick Children Research Institute, University of Toronto, Toronto, Canada; 4 Department of Orthopedagogy, University of Groningen, Groningen, The Netherlands; 5 Department of Epidemiology, University of Groningen, University Medical Center Groningen, Groningen, The Netherlands; 6 Department of Neonatology, Emma Children's Hospital Academic Medical Center, Amsterdam, The Netherlands; 7 Department of Neonatology, Wilhelmina Children's Hospital, University Medical Center Utrecht, Utrecht, The Netherlands; 8 Princess Amalia Department of Pediatrics, Department of Neonatology, Isala, Zwolle, The Netherlands; 9 Division of Neonatology, Department of Pediatrics, UMC St. Radboud Nijmegen, Nijmegen, The Netherlands; 10 Department of Pediatrics, Máxima Medical Center, Veldhoven, The Netherlands; 11 Erasmus MC, Sophia Children's Hospital, Rotterdam, The Netherlands; 12 Department of Pediatrics, Maastricht University Medical Center, GROW–School for Oncology and Developmental Biology, Maastricht, The Netherlands; 13 Department of Pediatrics, VU University Medical Center, Amsterdam, The Netherlands; University of Alabama at Birmingham, United States of America

## Abstract

**Background and Objective:**

High bilirubin/albumin (B/A) ratios increase the risk of bilirubin neurotoxicity. The B/A ratio may be a valuable measure, in addition to the total serum bilirubin (TSB), in the management of hyperbilirubinemia. We aimed to assess whether the additional use of B/A ratios in the management of hyperbilirubinemia in preterm infants improved neurodevelopmental outcome.

**Methods:**

In a prospective, randomized controlled trial, 615 preterm infants of 32 weeks' gestation or less were randomly assigned to treatment based on either B/A ratio and TSB thresholds (consensus-based), whichever threshold was crossed first, or on the TSB thresholds only. The primary outcome was neurodevelopment at 18 to 24 months' corrected age as assessed with the Bayley Scales of Infant Development III by investigators unaware of treatment allocation. Secondary outcomes included complications of preterm birth and death.

**Results:**

Composite motor (100±13 vs. 101±12) and cognitive (101±12 vs. 101±11) scores did not differ between the B/A ratio and TSB groups. Demographic characteristics, maximal TSB levels, B/A ratios, and other secondary outcomes were similar. The rates of death and/or severe neurodevelopmental impairment for the B/A ratio versus TSB groups were 15.4% versus 15.5% (P = 1.0) and 2.8% versus 1.4% (P = 0.62) for birth weights ≤1000 g and 1.8% versus 5.8% (P = 0.03) and 4.1% versus 2.0% (P = 0.26) for birth weights of >1000 g.

**Conclusions:**

The additional use of B/A ratio in the management of hyperbilirubinemia in preterm infants did not improve their neurodevelopmental outcome.

**Trial Registration:**

Controlled-Trials.com ISRCTN74465643

## Background

Free bilirubin (Bf), i.e. the fraction of bilirubin not bound to albumin, crosses the blood-brain barrier and exhibits neurotoxicity. In accordance, the total bilirubin load together with Bf is thought to predict bilirubin neurotoxicity more reliably than the total serum bilirubin (TSB), as assessed by clinical and electrophysiological parameters, i.e. neurodevelopmental outcome and maturation of automated brain stem responses, respectively. [1]–[4] Nevertheless, only TSB and not Bf is incorporated in the clinical management of hyperbilirubinemia. Neither is it routine practice to measure Bf in clinical laboratories nor are FDA approved instruments commercially available. The bilirubin (TSB)/albumin (B/A) ratio, which could be a surrogate for Bf, might be a good additional parameter to TSB, to indicate an increased risk of bilirubin-induced neurotoxicity in preterm infants. To date, mainly retrospective data have favored an additional role for high B/A ratios as risk factors for bilirubin-induced neurotoxicity and only limited data exist regarding B/A ratios in the management and neurodevelopmental outcome of preterm infants with unconjugated hyperbilirubinemia. [5] What is lacking are prospective clinical trials on the use of the B/A ratio in jaundiced preterm infants to evaluate its safety and clinical utility in preventing or reducing long-term bilirubin-induced neurotoxicity.

We conducted a prospective, multicenter, randomized controlled trial on the management of preterm infants with hyperbilirubinemia to determine the effects of the additional use of B/A ratios on their neurodevelopmental outcome at 18 to 24 months after the expected date of delivery.

## Methods

### Patients

Preterm infants of 31^+6^ weeks' gestation or less (based on the first day of the last menstruation), were eligible for enrollment after admission to a neonatal intensive care unit (NICU) during the first 24 hours after birth. Exclusion criteria were major congenital malformations, clinical syndromes, or chromosomal abnormalities.

### Ethics Statement

The Medical Ethics Review Board of the University Medical Center Groningen, the Netherlands, and all ten Dutch NICUs approved the study. Written informed consent was obtained from the parents or guardian of each participating infant. This study was conducted according to the principles expressed in the Declaration of Helsinki. This study is registered in the ISRCTN Register, number 74465643.

The protocol for this trial and supporting CONSORT checklist and flowchart are available as supporting information (see **Checklist S1**, **Flowchart S1** and **Protocol S1**).

### Randomisation and masking

Infants were stratified on the basis of center and gestational age (24^+0^ to 28^+6^ and 29^+0^ to 31^+6^ weeks), and were randomly assigned by neonatologists to a treatment group (balance [1∶1]) by a centralized computer system. Parents, care givers, and those assessing the outcomes were blinded to group assignment.

### Treatment

During the first ten days hyperbilirubinemia in infants in the B/A ratio group was evaluated daily using the B/A ratio together with TSB. Treatment decisions were based on the B/A ratio and the TSB level, whichever threshold was crossed first. We chose to use both thresholds in this study group because we considered the evidence from previous studies, regarding the safety of B/A ratio thresholds insufficient, to justify ignoring TSB thresholds.

TSB and albumin levels were also measured daily for infants in the TSB group (control group), but treatment was based on the TSB level only. TSB and albumin were measured using routine analytical methods. [6]

The TSB treatment thresholds (in μmol/L) for phototherapy (PT) and exchange transfusion were uniform and consensus-based. The B/A ratio thresholds (in μmol/L divided by g/L = μmol/g) were also uniform and derived from the corresponding TSB values and albumin levels of 25 g/L and 30 g/L for infants with birth weights of < or >1250 g, respectively. [7] Treatment nomograms depended on infants' birth weights and risk factors (see **Figure S1**), and national guidelines for phototherapy and exchange transfusions were available on line. [8]

### Assessments

The primary outcome was the composite motor score at 18 to 24 months' corrected age because previous retrospective data analyses of the National Institute of Child Health and Human Development (NICHD) Phototherapy Trial showed that peak bilirubin levels correlated with psychomotor developmental indices (PDI). [9] The 3rd Version of the Bayley Scales of Infant and Toddler Development (BSID III) was used to assess fine and gross motor as well as cognitive functions by investigators who were unaware of the treatment allocations. Standardized, pediatric, neurologic evaluations were performed at the same age as the BSID III assessments. Cerebral palsy and mild neurological dysfunction were diagnosed using international criteria and definitions. [10] Hearing and vision were assessed with standardized tests.

Secondary outcomes included death, neurodevelopmental impairment (NDI), pre-defined clinical diagnoses, and potential adverse events. Neurodevelopmental impairment was defined as a composite motor score of <85 or a composite mental score of <85, any degree of cerebral palsy, any visual impairment, or any hearing impairment. Severe NDI was defined as a composite motor score of <70 or a composite mental score of <70, moderate or severe cerebral palsy, bilateral blindness, or bilateral hearing loss.

### Statistical analysis

In a consensus meeting of the Netherlands Neonatology Research Network a difference between the two treatment groups on the composite motor score ≥7 points was considered clinically relevant. To detect this on a 100 point scale, SD 26, alpha 0.05, power 0.80, we planned to evaluate 434 infants. Accounting for 10% mortality and 20% incomplete follow-up, a total of 614 subjects needed to be included, i.e. 307 per group. Intentions to treat analyses were performed.

We pre-specified two subgroups: birth weights ≤1000 g and birth weights of >1000 g with high B/A ratios. We selected these two birth weight groups because previous NICHD data showed an increase in mortality of approximately 20% in infants with birth weights ≤ 1000 g who had received PT. [11]

Another subgroup analysis was performed to determine the contrast between the two groups. Exploratory analyses were performed of survivors and non-survivors.

We used the *t* test or Mann-Whitney test to compare continuous variables and the chi-square test or Fisher exact test for categorical variables. Repeatedly measured variables, such as TSB, albumin and B/A ratio's, were pooled for each infant; mean, maximum or trough value's were calculated for each infant and used for further analyses. A two-sided P value of <0.05 was considered statistically significant. Statistical analyses were performed with SPSS 18.0 software for Windows (SPSS Inc, Chicago, IL).

We did not plan an interim analysis on the primary outcome since these data would not be available before 18 to 24 months. An independent data and safety monitoring committee reviewed serious interim results, including death and other adverse outcomes of prematurity, and the rate of exchange transfusions after inclusion of 200 infants.

## Results

**Figure 1:**
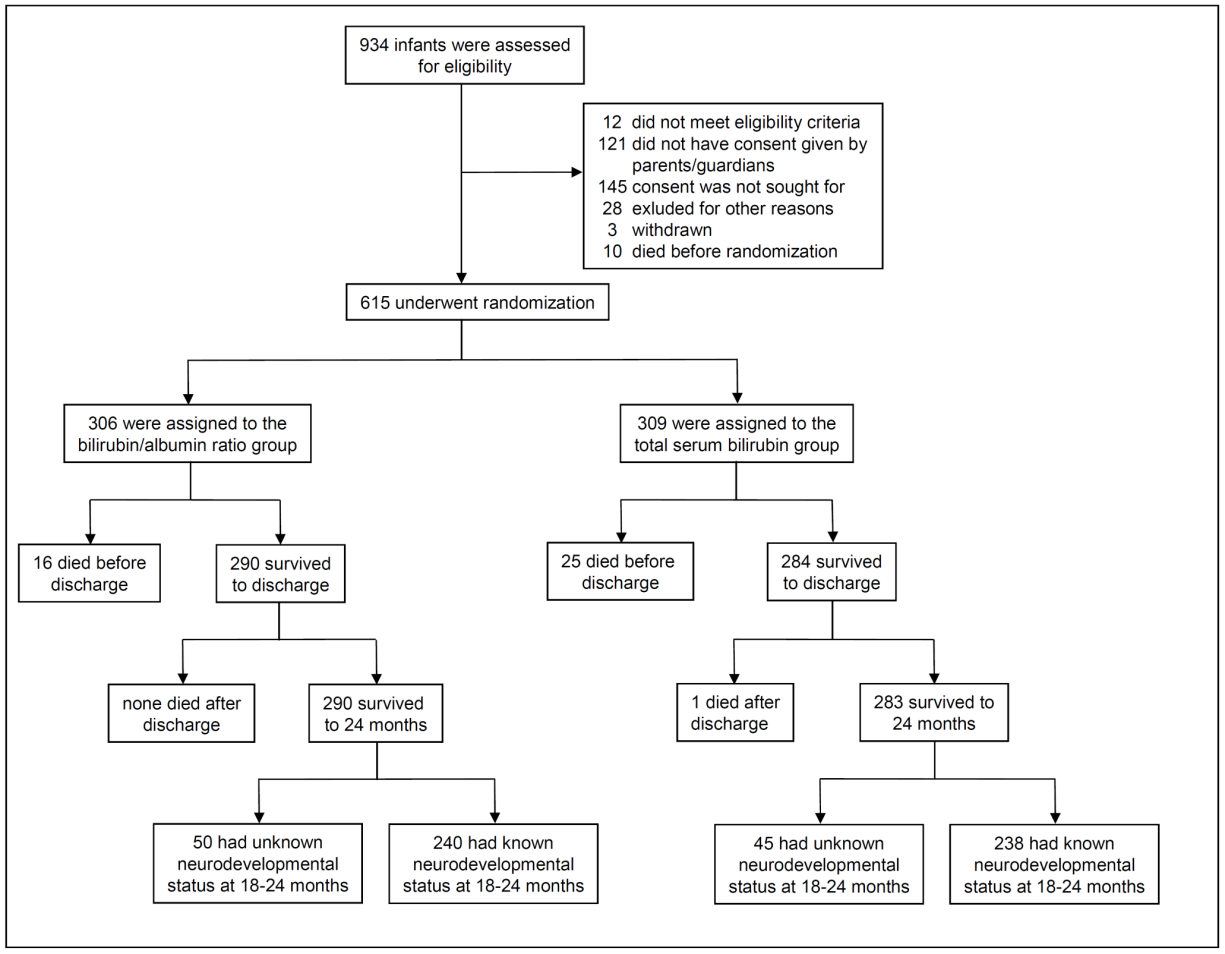
Enrollment, randomization, and follow-up. At follow up there were no significant baseline differences between infants with known and those with unknown neurodevelopmental status.

Between April 2007 and April 2008, 615 (66%) out of 934 eligible infants were enrolled (**Figure 1**). On the whole, the B/A ratio and TSB groups were similar with regards to the baseline characteristics (**Table 1**).

**Table 1:** Baseline characteristics at randomization.

	Bilirubin/albumin ratio group	Total serum bilirubin group
Characteristics	n = 306	n = 309
Gestational age – wk	29±2	29±2
Birth weight – g	1264±360	1250±330
Small for gestational age – no.(%)	70 (23)	73 (24)
Male sex – no. (%)	171 (56)	181 (59)
Multiple birth – no.(%)	98 (32)	104 (34)
Race or ethnicity ^#^ – no./total no.(%)		
- Caucasian	253/299 (83)	263/304 (85)
- Mediterranean	12/299 (4)	11/304 (4)
- Asian	10/299 (3)	14/304 (5)
- African	15/299 (5)	13/304 (4)
- Latin-American	9/299 (3)	3/304 (1)
Antenatal steroids - no./total no.(%)	257/290 (89)	246/295 (83)
Caesarean delivery – no./total no.(%)	139/304 (46)	138/297 (46)
5 min. Apgar score <3 – no./total no.(%)	5/302 (2)	6/306 (2)
Positive Coombs test – no. (%)	1 (0.3)	1 (0.3)
Maternal irregular antibodies – no./total no.(%)	8/197 (4)	5/202 (2)

Plus-minus values are means ± standard deviations. The denominator used to calculate the percentages of infants or mothers with a specific characteristic was the number for whom the characteristic was known. This number was the total number in each group, unless otherwise specified. No significant differences were found between the two groups. # Race or ethnicity was reported by patients' parents or guardians or determined by the physician on reviewing the charts.

### Outcome at 18 to 24 months

**Table 2** shows the neurodevelopmental outcome at 18 to 24 months' corrected age for 478 children, i.e. 78% of the 615 randomized infants and 83% of the 573 children who survived until neurodevelopmental testing (see also **Table S1**). The primary outcome, i.e. the mean (± SD) composite motor score for the two groups was similar. The number of children with a composite motor score of <85 was also similar: 12 (5.1%) out of 237 in the B/A ratio group versus 9 (3.7%) out of 243 in the TSB group (P = 0.51). We found no differences between the two groups on the fine and gross motor scales, nor on the composite cognitive scale. Moreover, the additional use of the B/A ratio did not reduce the rate of NDI, severe or otherwise.

**Table 2:** Outcomes at 18 to 24 months.

	B/A ratio group	TSB group	P value^#^	RR (95%CI)	P value^$^
**Primary outcome**					
Composite motor score	100±13	101±12	0.49		
- Fine motor scale	11±3	12+3	0.28		
- Gross motor scale	9±2	9±2	0.95		
Composite cognitive score	101±12	101±11	0.61		
**Secondary outcome**					
Death	16/306 (5)	26/309 (8)	0.15	0.62 (0.34–1.14)	0.12
Severe neurodevelopmental impairment	10/264 (4)	5/269 (2)	0.20	2.04 (0.71–5.88)	0.19
Neurodevelopmental impairment	49/264 (19)	59/269 (22)	0.39	0.85 (0.60–1.19)	0.33
- Composite motor score <70	4/237 (2)	2/243 (1)	0.44	2.05 (0.38–11.1)	0.40
- Composite motor score <85	12/237 (5)	9/243 (4)	0.51	1.37 (0.59–3.18)	0.47
- Composite cognitive score <70	4/256 (2)	0/264 (0)	0.06	9.28 (0.50–171)	0.13
- Composite cognitive score <85	10/256 (4)	11/264 (4)	1.0	0.94 (0.41–2.17)	0.88
Cerebral palsy	8/264 (3)	4/269 (1)	0.26	2.04 (0.62–6.69)	0.24
Severe hearing loss	1/264 (<1)	1/269(<1)	1.0	1.02 (0.06–16.2)	0.99
Any hearing impairment	9/264 (3)	12/269 (5)	0.66	0.76 (0.33–1.78)	0.53
Severe visual impairment	0/264 (0)	1/269 (<1)	1.0	0.34 (0.014–8.3)	0.51
Any visual impairment	18/264 (7)	23/269 (9)	0.52	0.80 (0.44–1.44)	0.45

Plus-minus values are means ± standard deviations. The denominator used to calculate the percentage of infants with a specific outcome was the number of infants randomly assigned to each treatment group for whom that outcome was known at 18 to 24 months. This number was the total number in each group, unless otherwise specified. The motor and cognitive scores were assessed with the BSID III (scores range from 50 to 150, where 150 indicates most advanced development).

The relative risk of each outcome was calculated for the BA ratio group as compared to the TSB group. In the B/A ratio group the mean (±SD) age at death was 30±16 days and 10±7 days in the TSB group. Severe NDI was a composite motor score of <70, a composite cognitive score of <70, moderate or severe cerebral palsy, severe unilateral or bilateral hearing loss, or unilateral or bilateral blindness. Neurodevelopmental impairment was a composite motor score of <85, a composite cognitive score of <85, any neurological impairment, any visual impairment, or hearing impairment.

P value#: Outcomes of t-test for continuous variables or Fischer exact test for categorical variables, two-tailed; RR is relative risk with (95% confidence interval). P value ^$^: corresponding P value.

The two groups did not differ regarding TSB levels and B/A ratios (**Table 3**) and the number and duration of PT sessions was similar. Two infants in the B/A ratio group received an exchange transfusion (one based on TSB, one based on TSB and B/A ratio thresholds) against none in the TSB group.

**Table 3:** Bilirubin-related course and secondary outcomes during hospitalization.

	B/A ratio group	TSB group	P value^#^	RR (95%CI)	P value^$^
Neonatal course	n = 306	n = 309			
Peak TSB - μmol/l	179±44	181±46	0.66		
Mean TSB - μmol/l	124±35	127±36	0.51		
Trough albumin - g/L	25.5±5.2	25.4±5.2	0.76		
Mean albumin – g/L	29.1±4.8	29.4±5.4	0.54		
Peak B/A-ratio - μmol/g	6.2±1.4	6.3±1.7	0.41		
Mean B/A-ratio - μmol/g	4.4±1.1	4.4±1.2	0.36		
Phototherapy – no. (%)	270 (88%)	268 (87%)	0.63		
Duration phototherapy - hrs	77±51	71±48	0.12		
Exchange transfusions – no.	2	0	0.25		
**Secondary outcome n/n (%)**					
Death before day 15	4/306 (1.3)	17/309 (5.5)	0.006*	0.24 (0.08–0.70)	0.009*
Death before discharge	16/306 (5.2)	25/309 (8.1)	0.20	0.64 (0.35–1.19)	0.16
Sepsis	91/304 (30)	88/308 (29)	0.72	1.05 (0.82–1.34)	0.71
Meningitis	2/300 (0.7)	1/306 (0.3)	0.25	2.0 (0.19–22)	0.56
Intraventricular hemorrhage, grades 3 or 4	15/304 (5)	19/307 (6)	0.60	0.80 (0.41–1.54)	0.50
Patent ductus arteriosus	92/306 (30)	98/309 (32)	0.87	0.95 (0.75–1.20)	0.66
PDA treated with surgical ligation	15/306 (5)	17/309(6)	0.86	0.89 (0.45–1.75)	0.74
Necrotizing enterocolitis	28/306 (9)	31/309 (10)	0.79	0.91 (0.56–1.48)	0.71
NEC treated surgically	11/306 (4)	12/309 (4)	1.0	0.93 (0.41–2.10)	0.85
Periventricular leucomalacia, grades 3 or 4	2/304 (0.7)	1/307 (0.3)	0.25	2.02 (0.18–22)	0.57
Bronchopulmonary dysplasia at 36 wks' postmenstrual age	26/306(9)	32/309 (10)	0.49	0.52 (0.50–1.34)	0.43
Retinopathy of prematurity grade 3 or higher	6/304 (2)	4/309 (1)	0.54	1.52 (0.43–5.35)	0.51
ALGO refer	14/269 (5)	16/270 (6)	0.85	0.88 (0.44–1.76)	0.72

Plus-minus values are means ± standard deviations. The denominator used to calculate the percentage of infants with a specific outcome was the number for whom the variable was known. This number was the total number in each group, unless otherwise specified. ALGO is automated auditory brainstem response. #: Outcome of t-test for continuous variables or Fischer exact test for categorical variables, two-tailed; RR relative risk of the B/A ratio group in comparison to the TSB group with (95% confidence interval) and corresponding ^$^ P-value. *p<0.05.

The mean (±SD) albumin levels were 27.8 (±5.5) versus 30.8 (±4) for infants with birth weights of <1250 g (n = 319) versus ≥1250 g (n = 291), respectively.

**Table 3** shows secondary outcomes during NICU hospitalization. Fewer infants allocated to the B/A ratio group died during the first two weeks. Although not statistically significant, the overall mortality rate until discharge also appeared somewhat lower in the B/A ratio versus the TSB group (RR, 0.64; 95% CI, 0.35 to 1.19; P = 0.16). This prompted us to analyze mortality in these subgroups in more detail.

### Subgroup analyses according to birth weights (Table S2)

Among the 162 infants with birth weights ≤1000 g the additional use of the B/A ratio did not reduce mortality. Among the 453 infants with birth weights of >1000 g, mortality in the B/A ratio group was significantly lower than in the TSB group (RR, 0.30; 95%CI: 0.10–0.92, P = 0.03).

The composite motor and cognitive scores were not affected by treatment allocation in the two birth weight groups. The reduction of approximately 10% in the composite outcome of death or NDI in children with birth weights of >1000 g who were allocated to the B/A ratio group was, therefore, attributed to the reduction in mortality. Correspondingly, fewer adverse secondary outcomes, e.g. sepsis, intraventricular hemorrhage, patent ductus arteriosus, bronchopulmonary dysplasia, and necrotizing enterocolitis were observed in this group, although the reduction in none of these morbidities was statistically significant. Adverse secondary outcomes tended to occur more frequently in the B/A ratio subgroup of infants weighing ≤1000 g. The 18% increase in the incidence of sepsis was statistically significant (RR, 1.5; 95% CI: 1.1–2.1, P = 0.027).

**Table S3** shows the results of the subgroup analysis regarding treatment based on B/A ratio. In the B/A ratio group, 30% of the infants received PT at least once on the basis of the B/A ratio and not on the basis of the TSB level. In retrospect, 25% of the infants in the TSB group had crossed the B/A ratio and not the TSB threshold, but were by group definition not treated with PT. Developmental outcomes did not differ between the two groups. We found a tendency towards fewer mortalities in the B/A ratio group: 7 (7.6%) out of 92 versus 13 (16.9%) out of 77 (RR, 0.45; 95% CI, 0.19 to 1.07, P = 0.09). Mortality was not reduced in the B/A ratio subgroup of infants who were not treated on the basis of the B/A ratio (RR, 0.75; 95% CI, 0.33 to 1.72, P = 0.52). Despite comparable TSB and albumin levels, peak and mean B/A ratios were significantly lower in the B/A ratio group, without any effect on the total duration of PT.

On the other hand, almost half (48%) of all infants in the B/A ratio group received PT at least once on the basis of the TSB level, and not on the basis of the B/A ratio.

### Relationship of bilirubin and outcome

Peak B/A ratios during the first ten days were >12% higher in the non-survivors compared to the survivors (P = 0.004); peak TSB levels and trough (±SD) albumin levels were significantly lower in the non-survivors (**Table S4**).

The peak B/A ratios and TSB levels of the survivors without hearing loss or with a composite motor score ≥85 were comparable to the infants with any amount of hearing loss or a composite motor score of <85.

## Discussion

In this multicenter trial we found no difference in the composite motor score at 18 to 24 months' corrected age between the infants randomly allocated to receive PT and/or exchange transfusion based on the additional use of the B/A ratio and the infants allocated to receive treatment based exclusively on TSB levels. Nor did we find differences in other clinical outcomes including death. In pre-specified subgroup analyses mortality among the infants with birth weights of >1000 g was significantly lower in those allocated to the B/A ratio group, while mortality in both subgroups of infants weighing ≤1000 g was similar. Furthermore, we observed no significant differences in any of the bilirubin parameters between the survivors with NDI, severe or otherwise, or hearing loss and those without NDI or hearing loss.

The absence of any effect of the additional use of the B/A ratio on infants' neurological development including hearing loss or mortality may be explained, at least partly, by similar TSB levels, similar B/A ratios, and no significant differences in PT treatment. In preterms, each of these variables is putatively involved in the risk of bilirubin-associated NDI or death. [11], [12] Considering the occurrence of bilirubin encephalopathy in preterms with low TSB levels the risk of developing bilirubin neurotoxicity is not determined by TSB alone. [13]–[15] Only free bilirubin, and perhaps the B/A ratio, are more closely associated with bilirubin neurotoxicity. [2], [5] Nevertheless, a correlation between peak TSB levels and NDI or death was found in infants with extremely low birth weights (ELBW). [9], [16] This finding is in agreement with the reduction in overall NDI at lower TSB levels in ELBW infants in the ‘aggressive’ PT part of a recent randomized controlled trial (RCT). [12] In this as well as in another RCT a trend was found toward increased mortality in ELBW infants who had received more PT. [11], [12], [17]

In our RCT fewer deaths in the B/A ratio subgroup of infants with birth weights of >1000 g was not associated with less PT (data not shown) or with any significant differences in neonatal morbidities. The observed difference in mortality seemed biologically unrelated to the management of hyperbilirubinemia. Moreover, this trial was not powered for mortality, and in particular not for mortality in subgroups, therefore caution should be exercised to interpret this finding, as it could be found by change. The mortality rate in this subgroup, 1.8%, was also less than the historical figure of approximately 6% kept by the Dutch Perinatal Registry for infants who had also been treated according to their TSB levels. Furthermore, the reduction in mortality between the two categories of our trial appeared to be consistent. We found a trend towards fewer deaths in the subgroup allocated to the B/A ratio group who had been treated at least once on the basis of their B/A ratios (−9.3%, P = 0.09) and who had statistically significant lower (mean) peak B/A ratios.

We cannot explain why mortality was not reduced in the B/A ratio subgroup with birth weights < 1000 g. We did not find significant differences in causes of death between the two treatment and birth weight categories, although there was a trend towards fewer infants who had necrotizing enterocolitis in the B/A ratio subgroup weighing >1000 g at birth (but not in the subgroup < 1000 g). Infants in this subgroup lived significantly longer compared to infants allocated to the TSB category (30±16 versus 10±7 days, P = 0.003). Overall, peak B/A ratios were significantly higher in the non-survivors, whereas TSB and albumin levels were actually lower. We do not know whether the infants who died had a reduced anti-oxidant capacity due to lower albumin and bilirubin levels. This finding supported our original hypothesis that the additional use of the B/A ratio is a valuable parameter in hyperbilirubinemia management in preterms. It is tempting to speculate that it may be more appropriately to treat infants with PT when bilirubin is more harmful and the infant brain is more vulnerable to bilirubin neurotoxicity, i.e. when albumin levels are low. The pathophysiological conditions may involve respiratory and circulatory failure and sepsis, as was previously described. [18] Oh et al. found that higher Bf levels are associated with a higher risk of death or adverse neurodevelopmental outcome regardless of clinical status, while in stable infants higher TSB levels were associated with better outcomes. This finding also supports the potential benefits of using the B/A ratio in addition to the TSB level. It is a more useful indicator of the Bf level than using the TSB level alone. [19], [20] Using the B/A ratio in addition to TSB levels may lead to reducing early mortality by a mechanism that is as yet unknown. Our unexpected finding that use of the B/A ratio in the management of preterms with birth weights of >1000 g reduces mortality needs to be confirmed by a larger study.

The findings of the current study also underscore the efficacy of phototherapy in managing hyperbilirubinemia in preterm infants: only two babies in the entire cohort received an exchange transfusion and none of the infants in the current cohort exhibited neurological findings of classical kernicterus.

How can our results be translated into clinical practice? Incorporating B/A ratios in management guidelines does not seem an essential parameter to improve neurodevelopmental outcome, but it may reduce mortality in infants weighing >1000 g at birth. More specifically, the additional use of pre-defined B/A ratios did not allow for earlier or more intensive treatment and lower TSB levels. This was, at least in part, what we expected and was related to its add-on use. Approximately half of all infants in the B/A ratio group received PT at least once on the basis of the TSB level crossing its threshold and not on the basis of the B/A ratio, which would have resulted in unintentional undertreatment when using the B/A ratio as the sole threshold for treatment decision. We found no evidence for safe B/A ratio thresholds that would justify the sole use of B/A ratio rather than TSB thresholds. The contrast between the two treatment groups was thus diluted. Higher pre-specified albumin levels and lower B/A ratios would have resulted in more contrast, i.e. earlier treatment based on the B/A ratio. Actually, the mean albumin levels were slightly higher than the albumin levels that we used to predefine the BA ratio threshold. Based on these relatively low differences, we anticipated a rather small contrast. Another explanation for the absence of an effect of the additional use of the B/A ratio on neurodevelopmental outcome could be the relatively low treatment thresholds: the majority of preterms received PT. To date, the applied thresholds were consensus–based. The results of our RCT provided some evidence of the benefits without causing any harm. We demonstrated that the additional use of the B/A ratio does not result in harmful PT.

### Conclusion

In preterm infants of 32 weeks' gestation or less with hyperbilirubinemia we found no significant effect of the additional use of B/A ratio compared to TSB-based treatment on the composite motor score at 18 to 24 months' corrected age.

## Supporting Information

Figure S1 **BARTrial bilirubin nomograms for B/A ratio's and TSB.** BARTrial bilirubin nomograms for phototherapy and exchange transfusion. Treatment nomograms for the B/A ratio group consist of B/A ratio and TSB nomogram, for the TSB group consists of only TSB nomograms and depend on infants' birth weights and risk factors. The TSB treatment thresholds (in μmol/L) for phototherapy (PT) and exchange transfusion. The B/A ratio thresholds (in μmol/L divided by g/L = μmol/g) were derived from the corresponding TSB values and albumin levels of 25 g/L and 30 g/L for infants with birth weights of < or >1250 g, respectively.
(PDF)

Table S1 **Characteristics of randomized versus evaluated infants.**
(PDF)

Table S2 **Stratified analysis of outcomes according to birth weight categories.**
(PDF)

Table S3 **Characteristics of B/A ratio treated versus not treated infants.**
(PDF)

Table S4 **Bilirubin-related values of different subgroups.**
(PDF)

Checklist S1 **CONSORT 2010 checklist of information of the BARTrial.**
(PDF)

Flowchart S1 **CONSORT 2010 Flow Diagram of the BARTrial.**
(PDF)

Protocol S1 **Study protocols of the BARTrial.**
(PDF)
