# Supplementary material for: The Bilirubin Albumin Ratio in the Management of Hyperbilirubinemia in Preterm Infants to Improve Neurodevelopmental Outcome: A Randomized Controlled Trial – BARTrial
Source: PLoS One. 2014 Jun 13;9(6):e99466. doi: 10.1371/journal.pone.0099466 (PMC4057208; doi:10.1371/journal.pone.0099466)
Supplement: Figure S1 — BARTrial bilirubin nomograms for B/A ratio's and TSB. BARTrial bilirubin nomograms for phototherapy and exchange transfusion. Treatment nomograms for the B/A ratio group consist of B/A ratio and TSB nomogram, for the TSB group consists of only TSB nomograms and depend on infants' birth weights and risk factors. The TSB treatment thresholds (in μmol/L) for phototherapy (PT) and exchange transfusion. The B/A ratio thresholds (in μmol/L divided by g/L = μmol/g) were derived from the corresponding TSB values and albumin levels of 25 g/L and 30 g/L for infants with birth weights of < or >1250 g, respectively. (PDF) [file pone.0099466.s001.pdf]

BARTrial bilirubin normograms for phototherapy and exchange transfusion

Total Serum Bilirubin (TSB)
and for Bilirubin:Albumin ratio (BA)

Birthweight catogories

<1000 g	BA ratio group	TSB and BA normograms
<1000 g	TSB group	TSB normogram
1000-1250 g	BA ratio group	TSB and BA normograms
1000-1250 g	TSB group	TSB normogram
1250-1500 g	BA ratio group	TSB and BA normograms
1250-1500 g	TSB group	TSB normogram
1500-2000 g	BA ratio group	TSB and BA normograms
1500-2000 g	TSB group	TSB normogram
>2000 g	BA ratio group	TSB and BA normograms
>2000 g	TSB group	TSB normogram

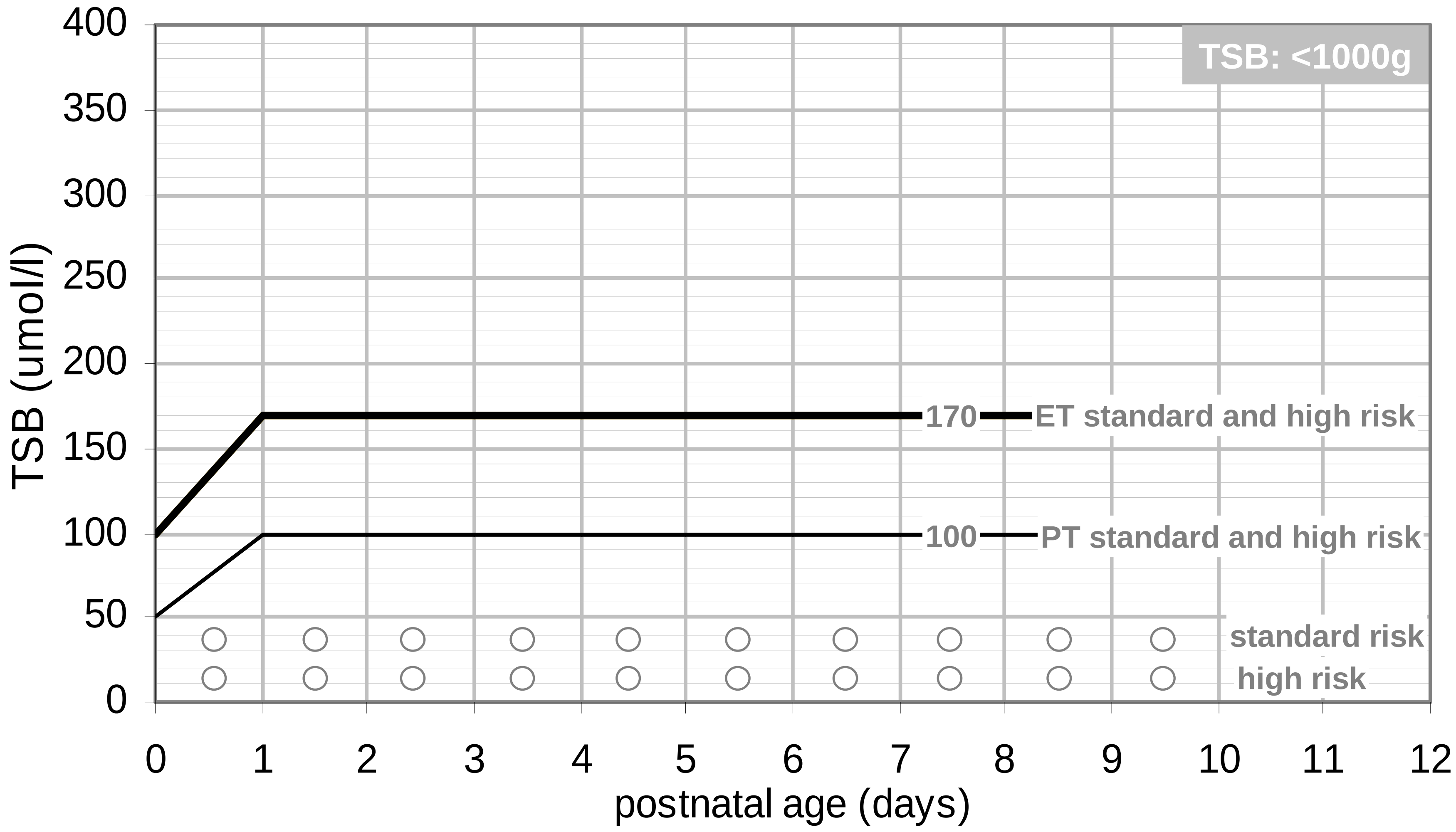

TSB: <1000g
TSB (umol/l)
400
350
300
250
200
150
100
50
0
170
ET standard and high risk
100
PT standard and high risk
standard risk
high risk
0
1
2
3
4
5
6
7
8
9
10
11
12
postnatal age (days)

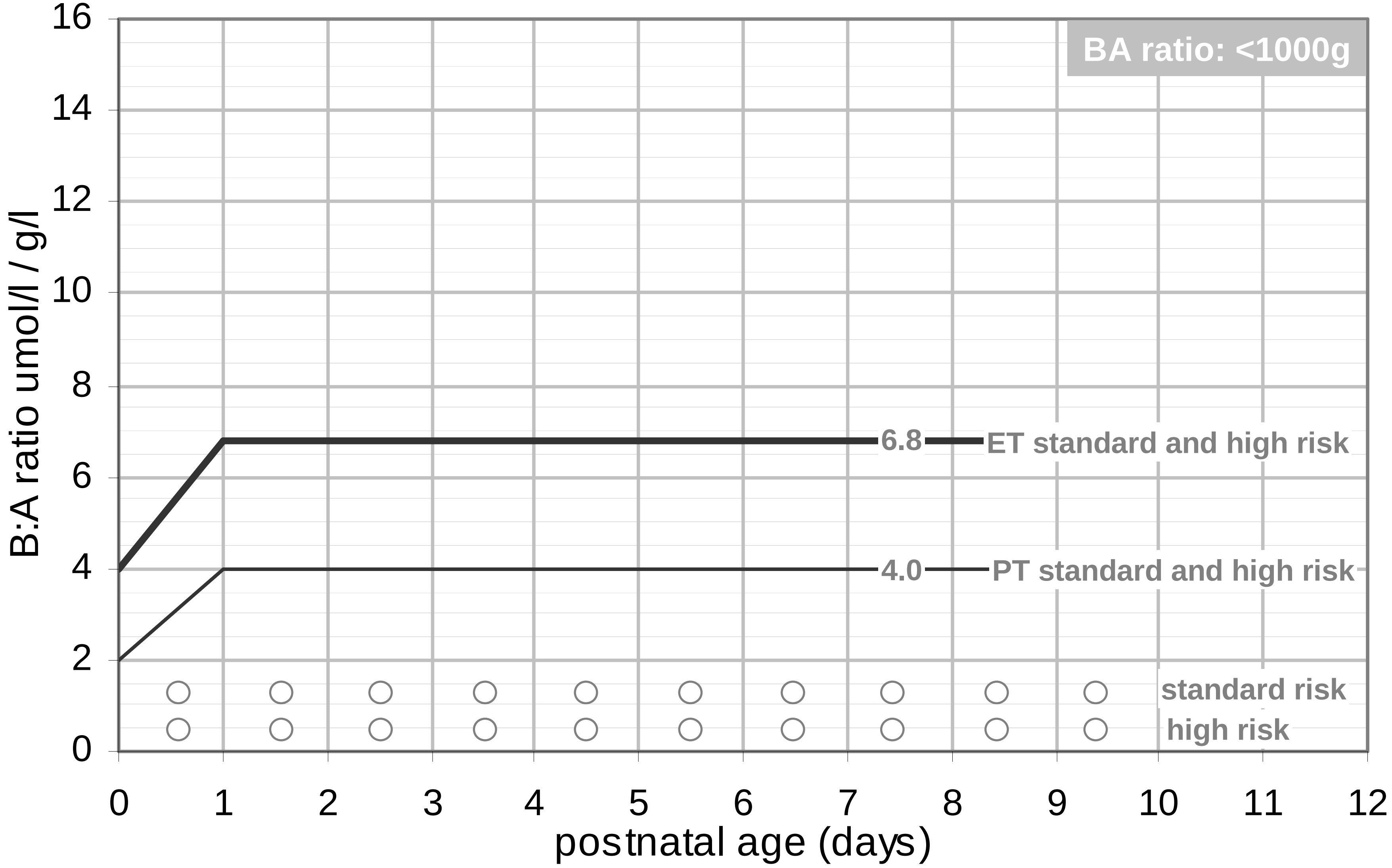

BA ratio: <1000g
B:A ratio umol/l / g/l
16
14
12
10
8
6
4
2
0
6.8
ET standard and high risk
4.0
PT standard and high risk
standard risk
high risk
0
1
2
3
4
5
6
7
8
9
10
11
12
postnatal age (days)

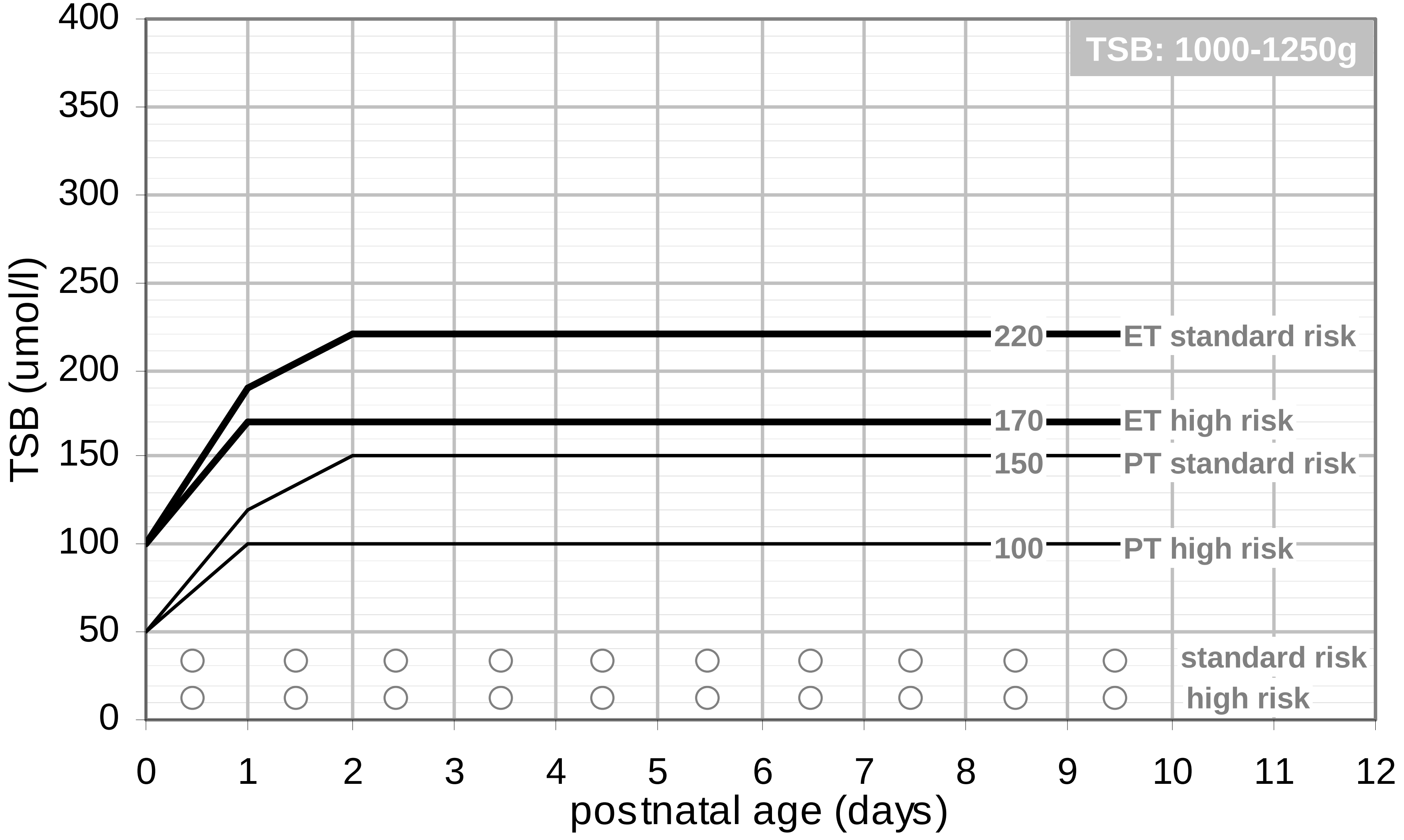

TSB: 1000-1250g
TSB (umol/l)
400
350
300
250
200
150
100
50
0
220
ET standard risk
170
ET high risk
150
PT standard risk
100
PT high risk
standard risk
high risk
0
1
2
3
4
5
6
7
8
9
10
11
12
postnatal age (days)

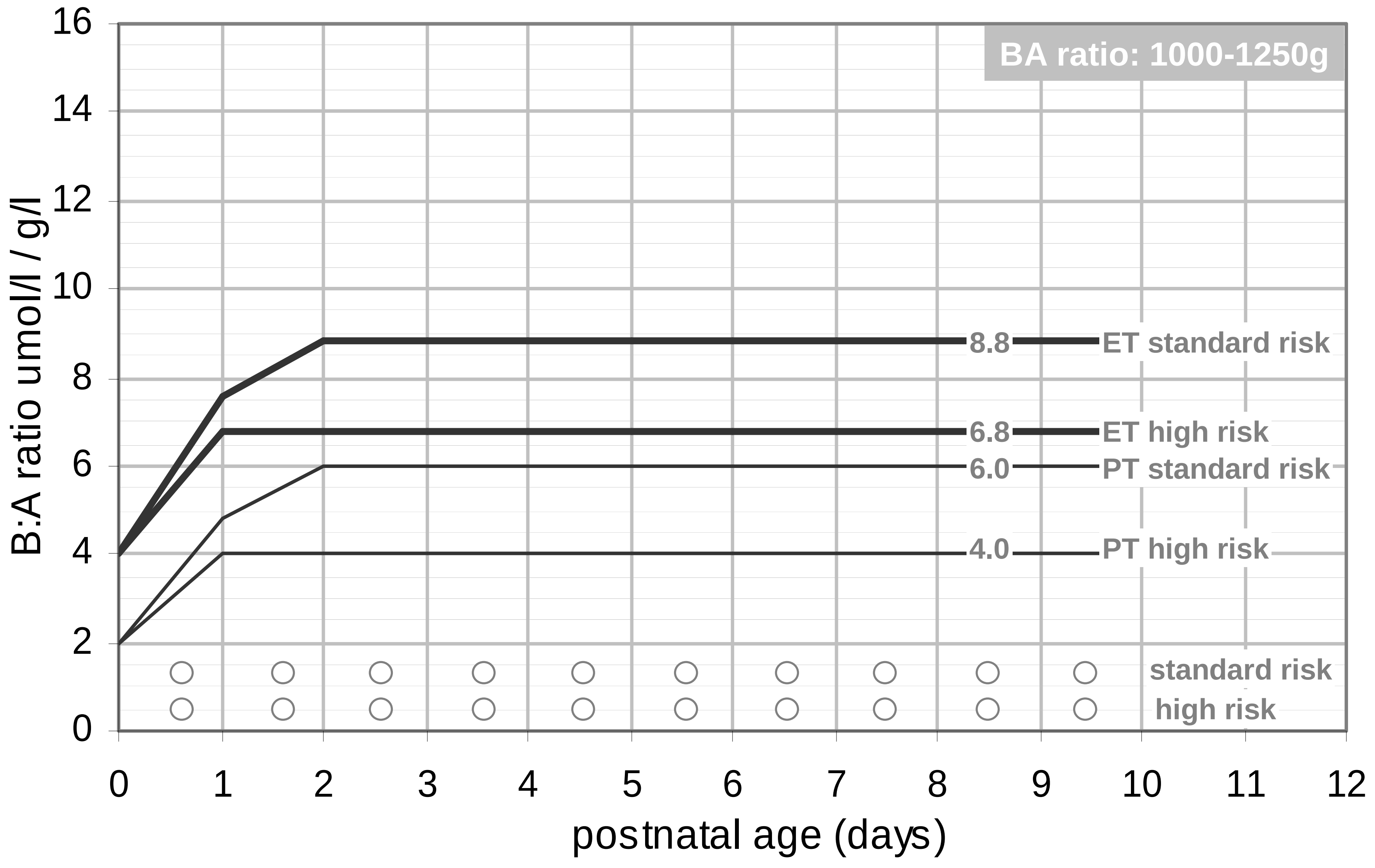
BA ratio: 1000-1250g
B:A ratio umol/l / g/l
postnatal age (days)
16
14
12
10
8
6
4
2
0
0
1
2
3
4
5
6
7
8
9
10
11
12
8.8
ET standard risk
6.8
ET high risk
6.0
PT standard risk
4.0
PT high risk
standard risk
high risk

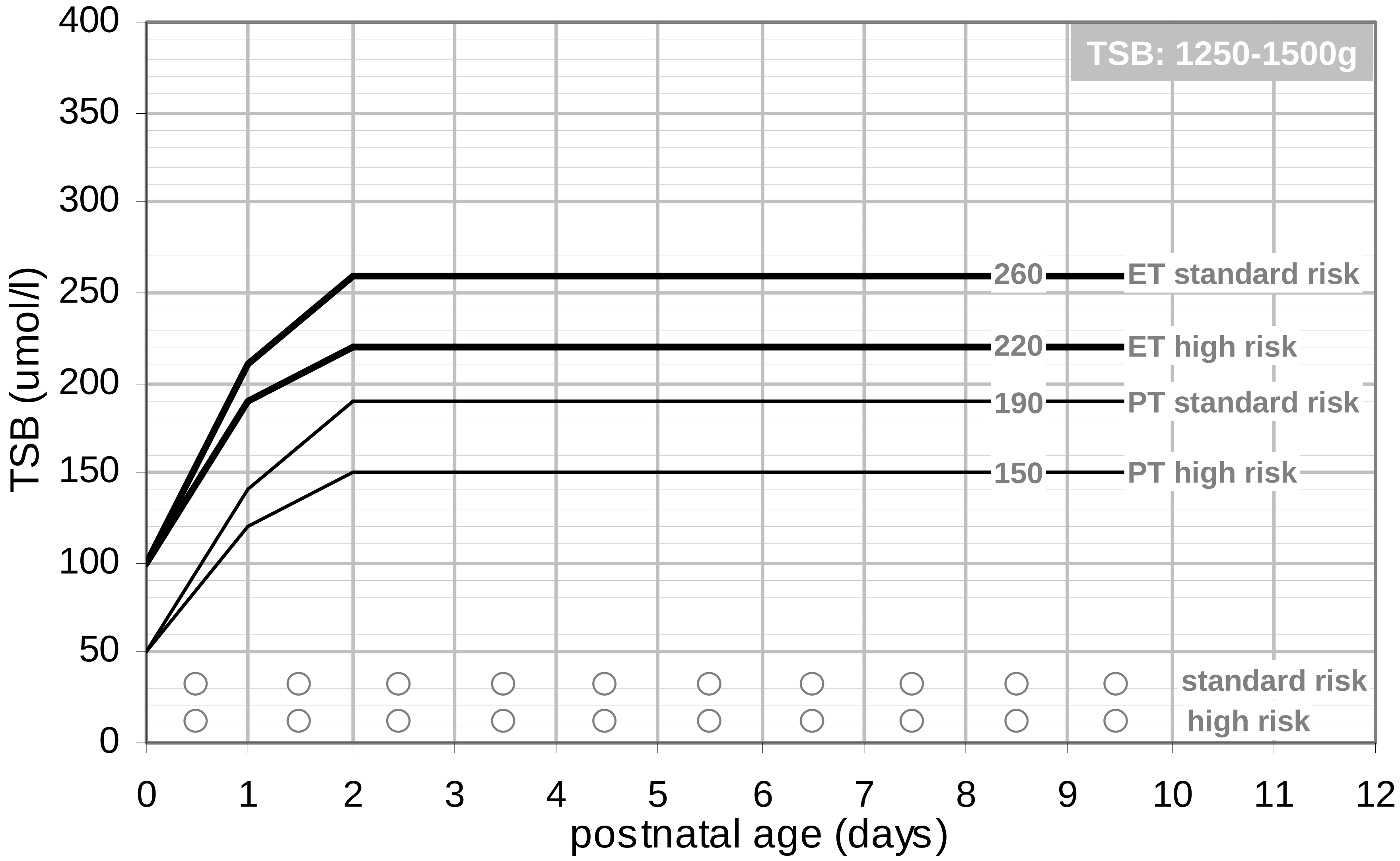
TSB: 1250-1500g
TSB (umol/l)
400
350
300
250
200
150
100
50
0
260
ET standard risk
220
ET high risk
190
PT standard risk
150
PT high risk
standard risk
high risk
0
1
2
3
4
5
6
7
8
9
10
11
12
postnatal age (days)

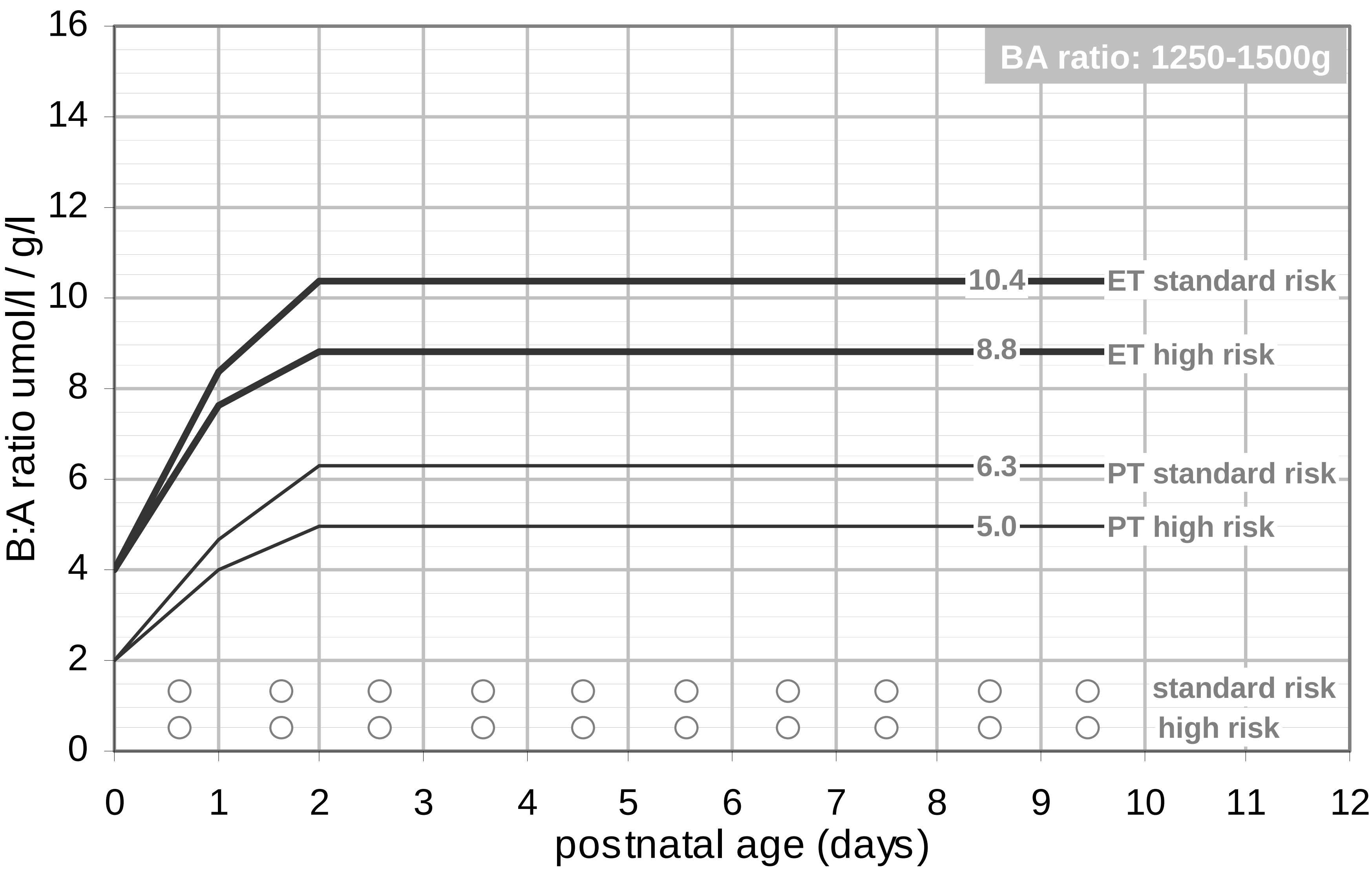

BA ratio: 1250-1500g
B:A ratio umol/l / g/l
16
14
12
10
8
6
4
2
0
10.4
ET standard risk
8.8
ET high risk
6.3
PT standard risk
5.0
PT high risk
standard risk
high risk
0
1
2
3
4
5
6
7
8
9
10
11
12
postnatal age (days)

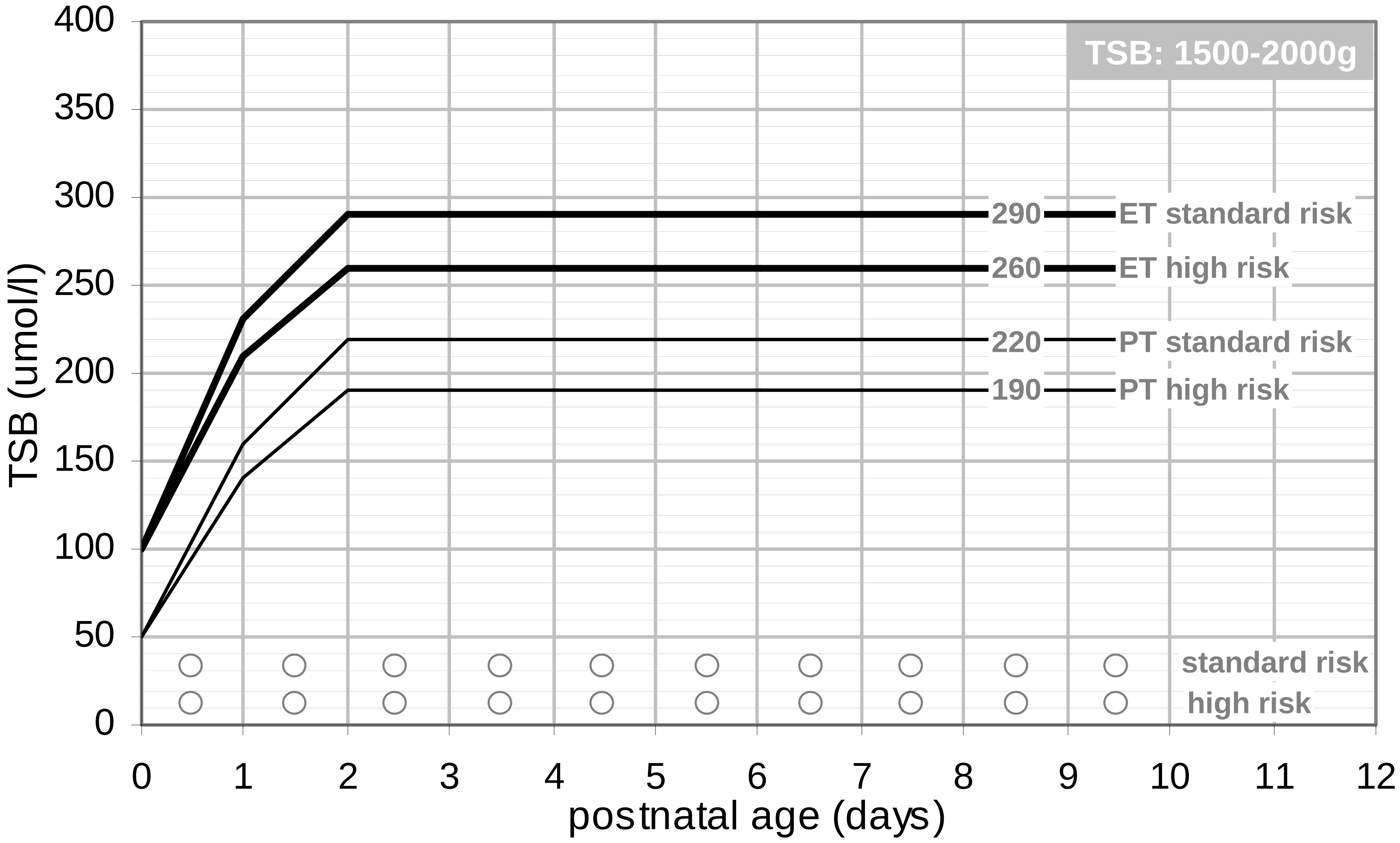
TSB: 1500-2000g
TSB (umol/l)
postnatal age (days)
290 ET standard risk
260 ET high risk
220 PT standard risk
190 PT high risk
standard risk
high risk
400
350
300
250
200
150
100
50
0
0 1 2 3 4 5 6 7 8 9 10 11 12

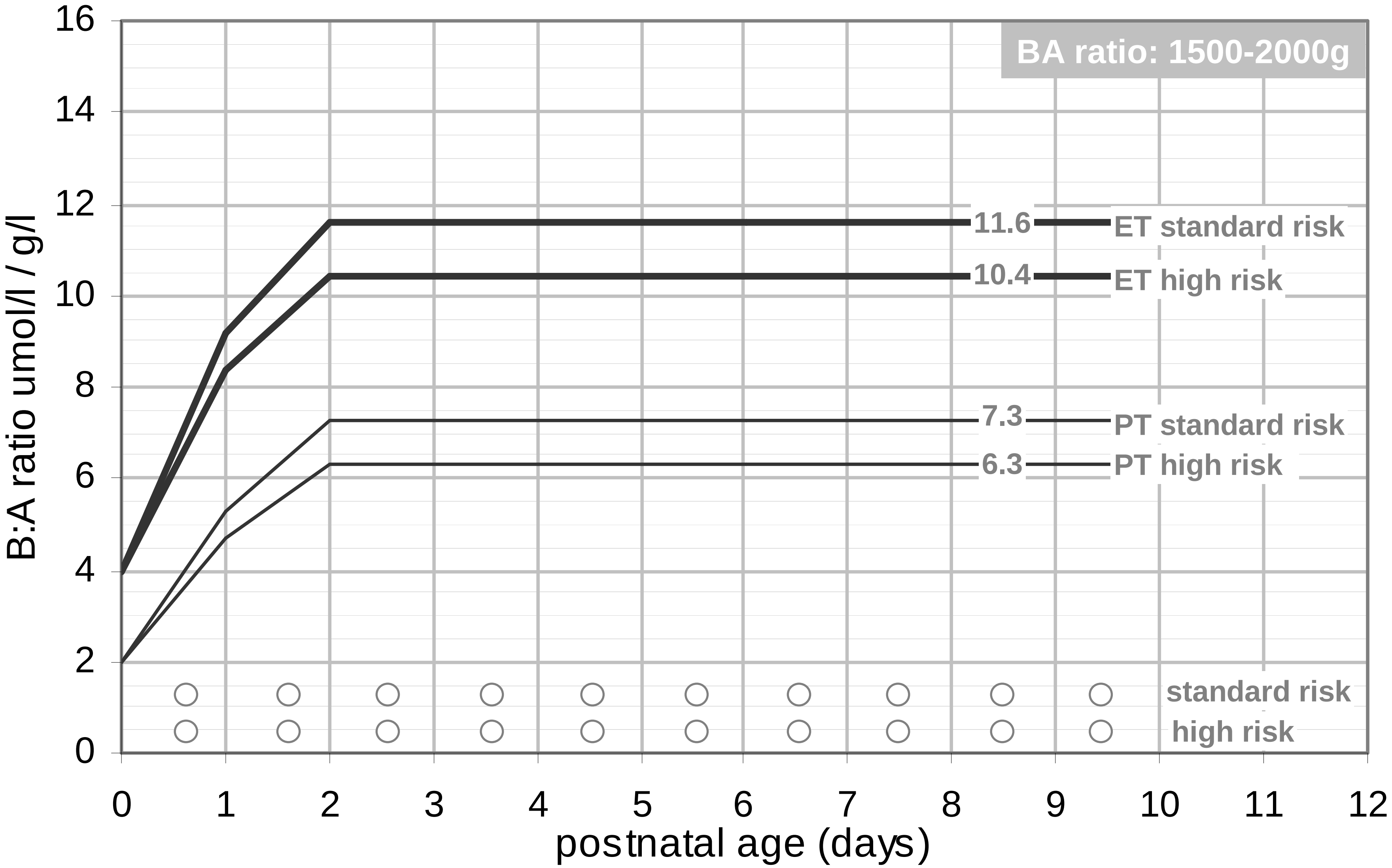
BA ratio: 1500-2000g
B:A ratio umol/l / g/l
postnatal age (days)
11.6
ET standard risk
10.4
ET high risk
7.3
PT standard risk
6.3
PT high risk
standard risk
high risk
0
2
4
6
8
10
12
14
16
0
1
2
3
4
5
6
7
8
9
10
11
12

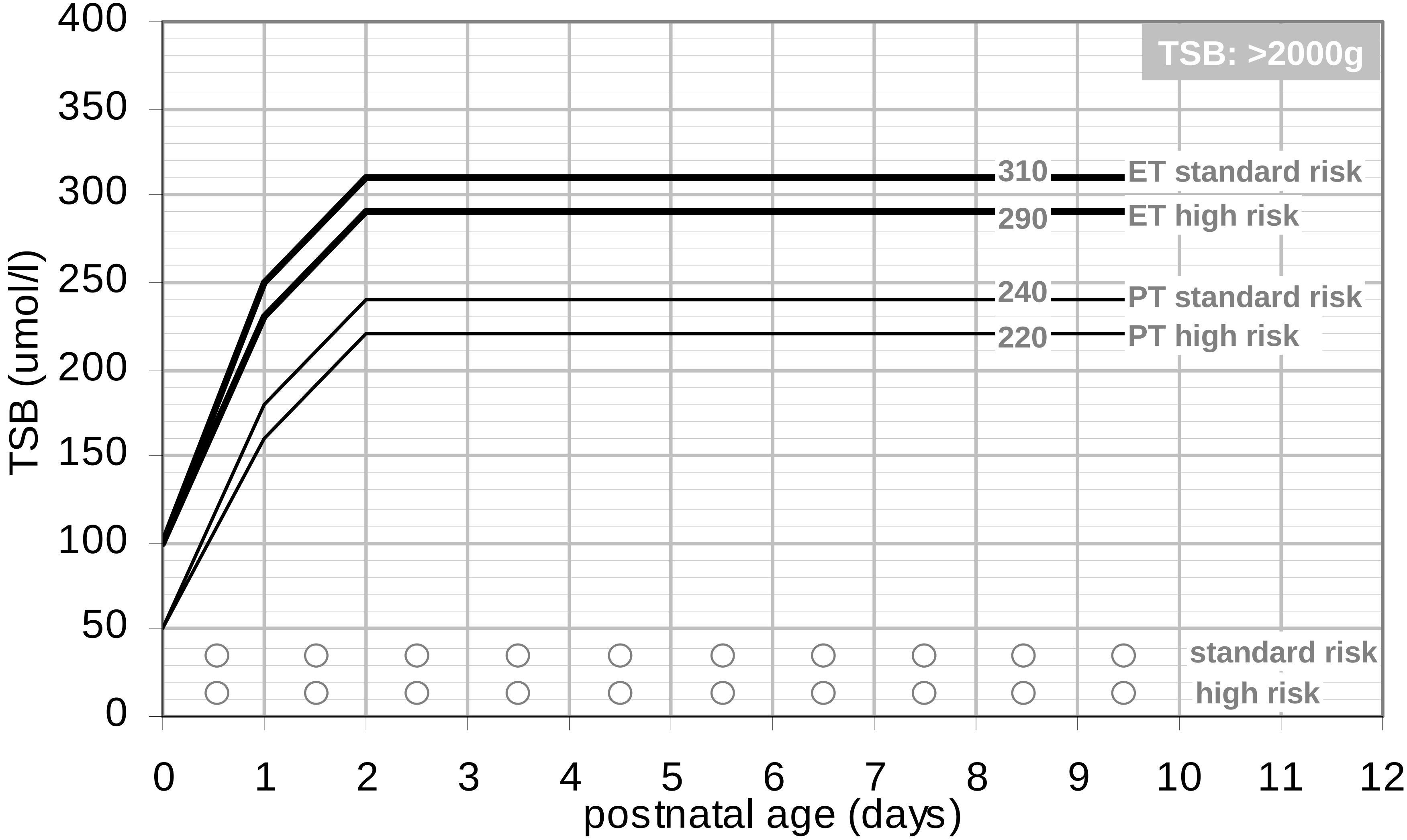
TSB: >2000g
310
ET standard risk
290
ET high risk
240
PT standard risk
220
PT high risk
standard risk
high risk
TSB (umol/l)
400
350
300
250
200
150
100
50
0
0
1
2
3
4
5
6
7
8
9
10
11
12
postnatal age (days)

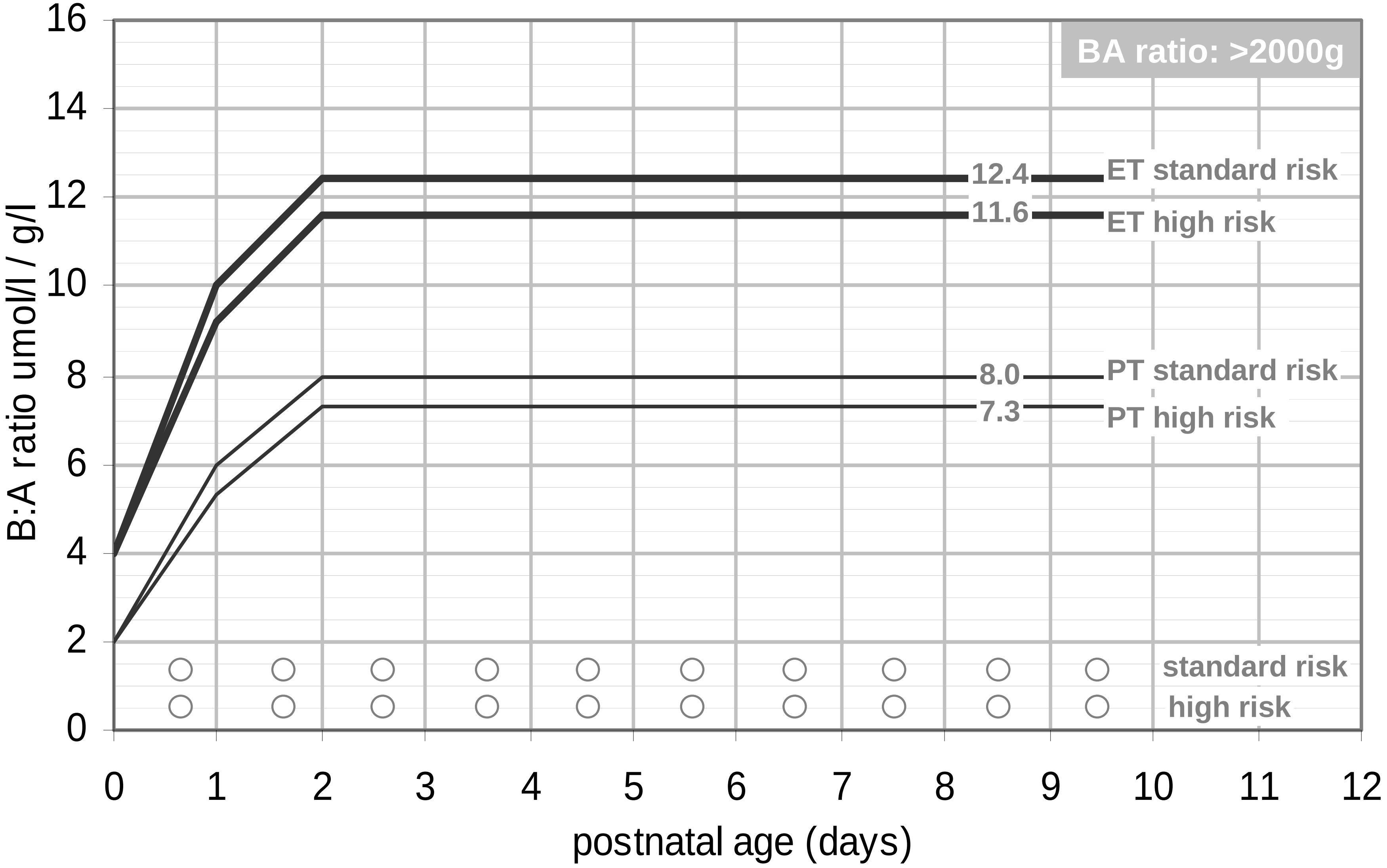

BA ratio: >2000g
B:A ratio umol/l / g/l
postnatal age (days)
12.4
ET standard risk
11.6
ET high risk
8.0
PT standard risk
7.3
PT high risk
standard risk
high risk
16
14
12
10
8
6
4
2
0
0
1
2
3
4
5
6
7
8
9
10
11
12

Guideline for the use of TSB and BA ratio normograms for phototherapy (PT) and exchange transfusion (ET). TSB in µmol/L, BA-ratio in µmol/L / g/L = µmol/g. TSB 17.1 µmol/L = 1 mg/L

1. Select appropriate normograms based on birthweight
2. Mark risk status (standard risk or high risk)
3. Mark TSB and BA ratio in normograms
4. Start phototherapy if TSB or BA is > PT-threshold
   Stop phototherapy if TSB and BA < PT-threshold
5. Consider exchange transfusion if TSB or BA reaches ET threshold despite intensive PT

High Risk (check daily)
- asfyxia: Apgar score < 3 at 5 min
- hypoxemia: PaO_2<40 mmHg (<5.3 kPa) > 2 hrs (recent 24 hrs)
- acidosis: pH < 7.15 >1 hr (recent 24 hrs)
- hemolysis with positive Coombs
- clinical of neurological deterioration (sepsis with use of vasopressors, meningitis, intracranial hemorrhage > gr 2

based on guidelines published by Ahlfors 1994 and Maisels 2003

Guideline for the use of TSB nomogram for phototherapy (PT) and exchange transfusion (ET).
TSB in µmol/L. 17.1 µmol/L = 1 mg/L

1. Select appropriate normogram based on birthweight
2. Mark risk status (standard risk or high risk)
3. Mark TSB in normogram
4. Start phototherapy if TSB > PT-threshold
   Stop phototherapy if TSB < PT-threshold
5. Consider exchange transfusion if ET-threshold
   is reached despite intensive PT

High Risk (check daily)
- asfyxia: Apgar score < 3 at 5 min
- hypoxemia: PaO_2<40 mmHg (<5.3 kPa) > 2 hrs (recent 24 hrs)
- acidosis: pH < 7.15 >1 hr (recent 24 hrs)
- hemolysis with positive Coombs
- clinical of neurological deterioration (sepsis with use of vasopressors, meningitis, intracranial hemorrhage > gr 2

based on guidelines published by Ahlfors 1994 and Maisels 2003

**BA ratio GROUP**	name………………………………………………	date of birth………………………….
**birthweigthgroup**	gestational age……………………………….	birthweight………………………….
**< 1000 g**	patiëntnumber…………………………………	studynumber………………………...

**BA ratio GROUP**	name………………………………………………	date of birth………………………….
**birthweigthgroup**	gestational age……………………………….	birthweight………………………….
**1000-1250 g**	patiëntnumber…………………………………	studynumber………………………...

**BA ratio GROUP**	name………………………………………………	date of birth………………………….
**birthweigthgroup**	gestational age……………………………….	birthweight………………………….
**1250-1500 g**	patiëntnumber…………………………………	studynumber………………………...

**BA ratio GROUP**	name………………………………………………	date of birth………………………….
**birthweigthgroup**	gestational age……………………………….	birthweight………………………….
**1500-2000 g**	patiëntnumber…………………………………	studynumber………………………...

**BA ratio GROUP**	name………………………………………………	date of birth………………………….
**birthweigthgroup**	gestational age……………………………….	birthweight………………………….
**>2000 g**	patiëntnumber…………………………………	studynumber………………………...

**TSB GROUP**	name……………………………………………	date of birth……………………………
**birthweigthgroup**	gestational age……………………………….	birthweight…………………………….
**<1000 g**	patiëntnumber………………………………….	studynumber………………………….
**TSB GROUP**	name……………………………………………	date of birth……………………………
**birthweigthgroup**	gestational age……………………………….	birthweight…………………………….
**1000-1250 g**	patiëntnumber………………………………….	studynumber………………………….
**TSB GROUP**	name……………………………………………	date of birth……………………………
**birthweigthgroup**	gestational age……………………………….	birthweight…………………………….
**1250-1500 g**	patiëntnumber………………………………….	studynumber………………………….
**TSB GROUP**	name……………………………………………	date of birth……………………………
**birthweigthgroup**	gestational age……………………………….	birthweight…………………………….
**1500-2000 g**	patiëntnumber………………………………….	studynumber………………………….
**TSB GROUP**	name……………………………………………	date of birth……………………………
**birthweigthgroup**	gestational age……………………………….	birthweight…………………………….
**>2000 g**	patiëntnumber………………………………….	studynumber………………………….

Guideline for the use of TSB and BA ratio nomograms for phototherapy (PT) and exchange transfusion (ET)

TSB in µmol/l and albumin in g/l and B:A ratio in µmol/l/g/l= µmol/g

1. Select appropriate nomogram based on birthweight
2. Mark risk status (standard risk of high risk)
3. Mark TSB and/or B:A ratio in nomogram
4. Start phototherapy if PT-threshold is reached
   Stop phototherapy if TSB is under PT-threshold
5. Consider exchange transfusion if ET-threshold is reached despite intensive PT

High risk (check daily)

asfyxia: Apgar score < 3 at 5 minutes

hypoxemia: PaO_2<40 mmHg (<5.3 kPa) > 2 hrs (recent 24 hrs)

acidosis: pH < 7.15 >1 hr (recent 24 hrs)

hemolysis with positive Coombs

clinical of neurological deterioration (sepsis with use of vasopressors, meningitis, intracranial hemorrhage > gr 2

based on guidelines published by Ahlfors 1994 and Maisels 2003

TSB: 17.1 umol/l = 1 mg/dl
