# Supplementary material for: The Bilirubin Albumin Ratio in the Management of Hyperbilirubinemia in Preterm Infants to Improve Neurodevelopmental Outcome: A Randomized Controlled Trial – BARTrial
Source: PLoS One. 2014 Jun 13;9(6):e99466. doi: 10.1371/journal.pone.0099466 (PMC4057208; doi:10.1371/journal.pone.0099466)
Supplement: Table S1 — Characteristics of randomized versus evaluated infants. (PDF) [file pone.0099466.s002.pdf]

Table S1. Characteristics of randomized versus evaluated infants

	BA ratio randomized	evaluated FU @ 2yrs	TSB randomized	Evaluated FU @ 2 yrs
Number	306	243	309	245
Gestational age – wk	29±2	29±2	29±2	29±2
Birth weight – g	1264±360	1271±348	1250±330	1276±320
Small for GA – n(%)	70 (23)	51 (21)	73 (24)	50 (20)
Male sex – n(%)	171 (56)	127 (52)	281 (59)	141 (58)
Multiple birth – n(%)	98 (32)	82 (34)	104 (34)	89 (36)
Caucasian – n/n(%)	253/299 (83)	210/241 (87)	563/304 (88)	220/245 (90)
Antenatal steroids- n/n(%)	257/290 (89)	205/230 (89)	246/295 (83)	201/233 (86)
Caesarean delivery -n/n(%)	139/304 (46)	112/241 (46)	138/297 (46)	105/236 (44)
Apgar score <3 – n/n(%)	5/302 (2)	2/239 (1)	6/306 (2)	4/242 (2)
TSB mean - µmol/l	124±35	127±34	127±36	129±35
TSB max - µmol/l	179±44	180±44	181±46	183±45
Albumin mean – g/l	29.1±4.8	29.4±4.6	29.4±5.4	29.7±4.6
Albumin trough – g/l				
B/A ratio mean - µmol/g	4.4±1.1	4.3±1.0	4.4±1.2	4.4±1.2
B/Aratio max - µmol/g	6.2±1.4	6.1±1.3	6.3±1.7	6.2±1.6
PT duratation – hrs	77±51	79±43	71±48	70±49

Baseline characteristics and bilirubin related variables of infants who were randomized versus the children evaluated at the age of 2 years. The number of evaluated children is the number of randomized children minus the number of deaths and number of children lost for follow up. For the BA ratio group: 306 infants were randomized, 16 died, 47 lost for follow up, 243 evaluated. For the TSB group 309 were randomized, 26 died, 38 lost for follow up, 245 evaluated.
Plus-minus values are means ± standard deviations and (numbers). The denominator used to calculate the percentage of infants with a specific characteristic was the number of infants randomly assigned to each group, and classified in each birth weight subgroup, for whom that characteristic was known. GA is gestational age. PT is phototherapy. FU @ 2 years is Follow-up at 2 years corrected age.
