# Supplementary material for: The Bilirubin Albumin Ratio in the Management of Hyperbilirubinemia in Preterm Infants to Improve Neurodevelopmental Outcome: A Randomized Controlled Trial – BARTrial
Source: PLoS One. 2014 Jun 13;9(6):e99466. doi: 10.1371/journal.pone.0099466 (PMC4057208; doi:10.1371/journal.pone.0099466)
Supplement: Table S2 — Stratified analysis of outcomes according to birth weight categories. (PDF) [file pone.0099466.s003.pdf]

Table S 2. Stratified analysis of outcomes according to birth weight categories

Outcome	B/A ratio group	TSB group	P
Composite motor score			
≤1000 g	100±11 (56)	100±11 (51)	0.89
>1000 g	101±13 (165)	102±13 (166)	0.49
Gross motor scale			
≤1000 g	9±3 (55)	9±2 (53)	0.83
>1000 g	9±2 (166)	9±2 (166)	0.70
Fine motor scale			
≤1000 g	11±2 (57)	11±3 (53)	0.40
>1000 g	11.34±2.60 (177)	12±3 (176)	0.44
Composite cognitive score			
≤1000 g	101±11 (58)	100±11 (57)	0.90
>1000 g	101±12 (182)	101±11 (181)	0.61
Composite motor score <70			
≤1000 g	0/68	0/64	1.00
>1000 g	4/169 (2.4%)	2/179 (1.1%)	0.44
Composite motor score <85			
≤1000 g	2/68 (2.9%)	1/64 (1.6%)	1.0
>1000 g	10/169 (5.9%)	8/179 (4.5%)	0.63
Composite cognitive score <70			
≤1000 g	0/70	0/70	1.00
>1000 g	4/186 (2.2%)	0/194	0.057
Composite cognitive score <85			
≤1000 g	1/70 (1.4%)	2/70 (2.9%)	1.00
>1000 g	9/186 (4.8%)	9/194 (4.6%)	1.00
Death			
≤1000 g	12/78 (15.4%)	13/84 (15.5%)	1.00
>1000 g	4/228 (1.8%)	13/225 (5.8%)	0.027*
Severe NDI #			
≤1000 g	2/71 (2.8%	1/72 (1.4%)	1.0
>1000 g	8/193 (4.1%)	4/197 (2.0%)	0.26
Death or severe NDI			
≤1000 g	14/71 (19.7%)	14/72 (19.4%)	1.00
>1000 g	12/193 (6.2%)	17/197 (8.6%)	0.44
NDI			
≤1000 g	13/71 (18.3%)	12/72 (16.7%)	0.83
>1000 g	36/193 (18.7%)	47/197 (23.9%)	0.22
Death or NDI			
≤1000 g	25/71 (35.2%)	25/72 (34.7%)	1.00
>1000 g	40/193 (20.7%)	60/197 (30.5%)	0.037*
Cerebral palsy			
≤1000 g	1/71 (1.4%)	0/72	0.50
>1000 g	7/193 (3.6%)	4/197 (2.2%)	0.38
Severe hearing loss			
≤1000 g	1/71 (1.4%)	0/72	0.50
>1000 g	0/193	1/197 (0.5%)	1.00
Any hearing impairment			
≤1000 g	2/71 (2.8%)	2/72 (2.8%)	1.00
>1000 g	7/193 (3.6%)	10/197 (5.1%)	0.62
Severe visual impairment			
≤1000 g	0/71	1/72 (1.4%)	1.00
>1000 g	0/193	0/197	1.00
Any visual impairment			
≤1000 g	3/71 (4.2%)	5/72 (6.9%)	0.72
>1000 g	15/193 (7.8%)	18/197 (9.1%)	0.72

Sepsis			
≤1000 g	43/78 (55%)	31/84 (37%)	0.027*
>1000 g	48/226 (21%)	57/224 (25%)	0.32
IVH all grades			
≤1000 g	26/78 (33%)	18/84 (21%)	0.11
>1000 g	41/226 (18.1%)	50/223 (22%)	0.29
IVH > grade 2			
≤1000 g	10/78 (12.8%)	10/84 (11.9%)	1.00
>1000 g	5/226 (2.2%)	9/223 (4.0%)	0.29
PDA			
≤1000 g	46/78 (59%)	44/84 (52%)	0.43
>1000 g	46/228 (20%)	54/225 (24%)	0.37
PDA surgery			
≤1000 g	10/78 (12.8%)	10/84 (11.9%)	1.00
>1000 g	5/228 (2.2%)	7/225 (3.1%)	0.57
NEC all			
≤1000 g	18/78 (23%)	12/84 (14.3%)	0.16
>1000 g	10/228 (4.4%)	19/225 (8.4%)	0.089
NEC surgery			
≤1000 g	8/78 (10.3%)	5/84 (6.0%)	0.39
>1000 g	3/228 (1.3%)	7/225 (3.1%)	0.22
CLD			
≤1000 g	30/78 (38%)	34/84 (40%)	0.87
>1000 g	19/228 (8.3%)	19/225 (8.4%)	1.00
BPD			
≤1000 g	18/78 (23%)	20/84 (24%)	1.00
>1000 g	8/228 (3.5%)	12/225 (5.3%)	0.37
PVL all			
≤1000 g	19/78 (24%)	20/84 (24%)	1.00
>1000 g	62/226 (27%)	62/223 (28%)	1.00
PVL > grade 2			
≤1000 g	1/78 (1.3%)	1/84 (1.2%)	1.00
>1000 g	1/226 (0.4%)	0/223	1.00
ROP all			
≤1000 g	21/78 (27%)	23/84 (27%)	1.00
>1000 g	12/226 (5.3%)	7/223 (3.1%)	0.35
ROP >2 or Plus			
≤1000 g	3/78 (3.8%)	3/84 (3.6%)	1.0
>1000 g	3/226 (1.3%)	1/223 (0.4%)	0.62
ALGO refer			
≤1000 g	5/62 (8.1%)	3/69 (4.3%)	0.48
>1000 g	9/207 (4.3%)	13/201 (6.5%)	0.39

Plus-minus values are means ± standard deviations and (numbers). The denominator used to calculate the percentage of infants with a specific outcome was the number of infants randomly assigned to each group, and classified in each birth weight subgroup, for whom the outcome was known. *:Outcome of Fischer exact test, two-tailed. # Severe NDI is a composite motor score of <70 or a composite cognitive score of <70, moderate or severe cerebral palsy, severe bilateral hearing loss or bilateral blindness. NDI is neurodevelopmental impairment is a composite motor score of <85 or a composite cognitive score of <85, any neurological impairment, any visual impairment, or any hearing impairment. IVH is intraventricular hemorrhage. PDA is persistent ductus arteriosus. NEC is necrotizing enterocolitis. CLD is chronic lung disease at 28 days. BPD is bronchopulmonary dysplasia at 36 weeks. PVL is periventricular leukomalacia. ROP is retinopathy of prematurity. ALGO is automated auditory brainstem response.
