# Supplementary material for: The Bilirubin Albumin Ratio in the Management of Hyperbilirubinemia in Preterm Infants to Improve Neurodevelopmental Outcome: A Randomized Controlled Trial – BARTrial
Source: PLoS One. 2014 Jun 13;9(6):e99466. doi: 10.1371/journal.pone.0099466 (PMC4057208; doi:10.1371/journal.pone.0099466)
Supplement: Table S3 — Characteristics of B/A ratio treated versus not treated infants. (PDF) [file pone.0099466.s004.pdf]

Table S3. Characteristics of B/A ratio treated versus not treated infants.

	B/A ratio	TSB	P value
**Baseline characteristics**	**n=92**	**n=77**	
Birth weight (g)	1258±364	1232±313	0.62
Gestational age (wks)	29±1.8	29±1.8	0.81
**Outcomes**			
Mortality (n/N (%))	7/92 (7.6%)	13/77 (16.9%)	0.09
Motor composite score	101±12	103±12	0.51
- Fine motor score	12±2	12±3	0.32
- Gross motor score	9±2	9±2	0.77
Cognitive composite score	102±12	102±10	0.82
NDI severe (n/N (%))	4/81 (4.9%)	0/68	0.38
NDI mild	15/81 (18.5%)	11/68 (16.2%)	0.83
NDI mild + severe	19/81 (23%)	11/68 (16.2%)	0.42
Death or severe NDI	11/81 (13.6%)	14/68 (21%)	0.28
**TSB, B/A, and Phototherapy**			
TSB max (µmol/L)	174±42	186±48	0.11
TSB mean (µmol/L)	121±36	127±35	0.26
B/A ratio max (µmol/g)	7.05±1.37	7.72±1.83	0.01*
B/A mean (µmol/g)	4.88±1.09	5.27±1.25	0.03*
Alb trough (g/L)	20.7±4.0	20.2±4.3	0.36
Alb mean (g/L	24.8± 4.3	24.4±4.1	0.51
PT duration (hrs)	87±60	77±55	0.29

Baseline characteristics, outcomes, and bilirubin-related course of infants who were treated at least once on the basis of their B/A ratio and not on the basis of TSB levels (n=92/306 (30%)) versus control infants who at least once had a B/A ratio that exceeded the B/A ratio threshold, but who were not treated accordingly (n=77/309(25%)).
Plus-minus values are means ± standard deviations. NDI is Neurodevelopmental impairment. Severe NDI is a composite motor score of <70 or a composite cognitive score of <70, moderate or severe cerebral palsy, severe bilateral hearing loss or bilateral blindness. Mild NDI is a composite motor score of <85 or a composite cognitive score of <85, any neurological impairment, any visual impairment, or any hearing impairment. PT is phototherapy. P values are the outcomes of either the *t* test or Fisher exact test. * p<0.05. B/A ratio group: n=92, 7 died, 11 lost to follow-up. TSB group: n=77, 13 died, 9 lost to follow-up.
