# Supplementary material for: The Bilirubin Albumin Ratio in the Management of Hyperbilirubinemia in Preterm Infants to Improve Neurodevelopmental Outcome: A Randomized Controlled Trial – BARTrial
Source: PLoS One. 2014 Jun 13;9(6):e99466. doi: 10.1371/journal.pone.0099466 (PMC4057208; doi:10.1371/journal.pone.0099466)
Supplement: Table S4 — Bilirubin-related values of different subgroups. (PDF) [file pone.0099466.s005.pdf]

Table S4. Bilirubin-related values of different subgroups

**Survivors versus non-survivors**	**group**<br>**Survivors** (n=572)	**group**<br>**Non-survivors** (n=42)[#]	**P**
TSB max (µmol/L)	181±44	166±47	0.036*
TSB mean (µmol/L)	127±35	111±35	0.005*
B/A ratio max (µmol/g)	6.19±1.48	6.91±2.38	0.004*
B/A mean (µmol/g)	4.37±1.12	4.65±1.56	0.145
Albumin trough (g/L)	25.8±4.9	20.5±5.8	0.000*
Albumin mean (g/L)	29.5± 4.5	25.6±9.3	0.011*
PT duration (hrs)	73±50	84±53	0.228
**Neurodevelopmentally non-impaired versus NDI survivors**			
	**Non-impaired** (n=383)	**NDI** (n=107)	
TSB max (µmol/L)	181±44	182±45	0.88
TSB mean (µmol/L)	128±35	129±35	0.67
B/A ratio max (µmol/g)	6.16±1.44	6.30±1.52	0.38
B/A mean (µmol/g)	4.34±1.06	4.50±1.13	0.18
Albumin trough (g/L)	26.1±5.1	25.4±4.8	0.19
Albumin mean (g/L)	29.7± 4.7	29.1±4.5	0.18
PT duration (hrs)	74±48	80±60	0.25
**Survivors with a composite motor scores ≥85 versus <85**			
	**CMS ≥85** (n=480)	**CMS<85** (n=21)	
TSB max (µmol/L)	181±45	170±39	0.29
TSB mean (µmol/L)	127±35	122±28	0.59
B/A ratio max (µmol/g)	6.20±1.47	5.97±1.61	0.48
B/A mean (µmol/g)	4.36±1.08	4.34±1.02	0.95
Albumin trough (g/L)	25.8±5.1	25.0±4.3	0.50
Albumin mean (g/L)	29.5± 4.7	29.0±4.11	0.66
PT duration (hrs)	77±50	85±86	0.56
**Survivors with normal hearing versus any hearing impairment**			
	**normal** (n=464)	**any hearing impairment** (n=19)	
TSB max (µmol/L)	181±43	194±55	0.24
TSB mean (µmol/L)	128±34	136±37	0.35
B/A ratio max (µmol/g)	6.18±1.42	6.79±2.11	0.07
B/A mean (µmol/g)	4.37±1.06	4.77±1.39	0.12
Albumin trough (g/L)	26±5	26.4±9.98	0.72
Albumin mean (g/L)	29.6± 4.6	29.4±5.74	0.85
PT duration (hrs)	75±52	66±29	0.47

Plus-minus values are means ± standard deviations. CMS is composite motor score. PT is phototherapy. #: One patient's TSB values are missing; *:Outcome of *t* test, two-tailed $p<0.05$
