# Supplementary figures and images for: The Bilirubin Albumin Ratio in the Management of Hyperbilirubinemia in Preterm Infants to Improve Neurodevelopmental Outcome: A Randomized Controlled Trial – BARTrial
Source: PLoS One. 2014 Jun 13;9(6):e99466. doi: 10.1371/journal.pone.0099466 (PMC4057208; doi:10.1371/journal.pone.0099466)

## CONSORT 2010 Flow Diagram

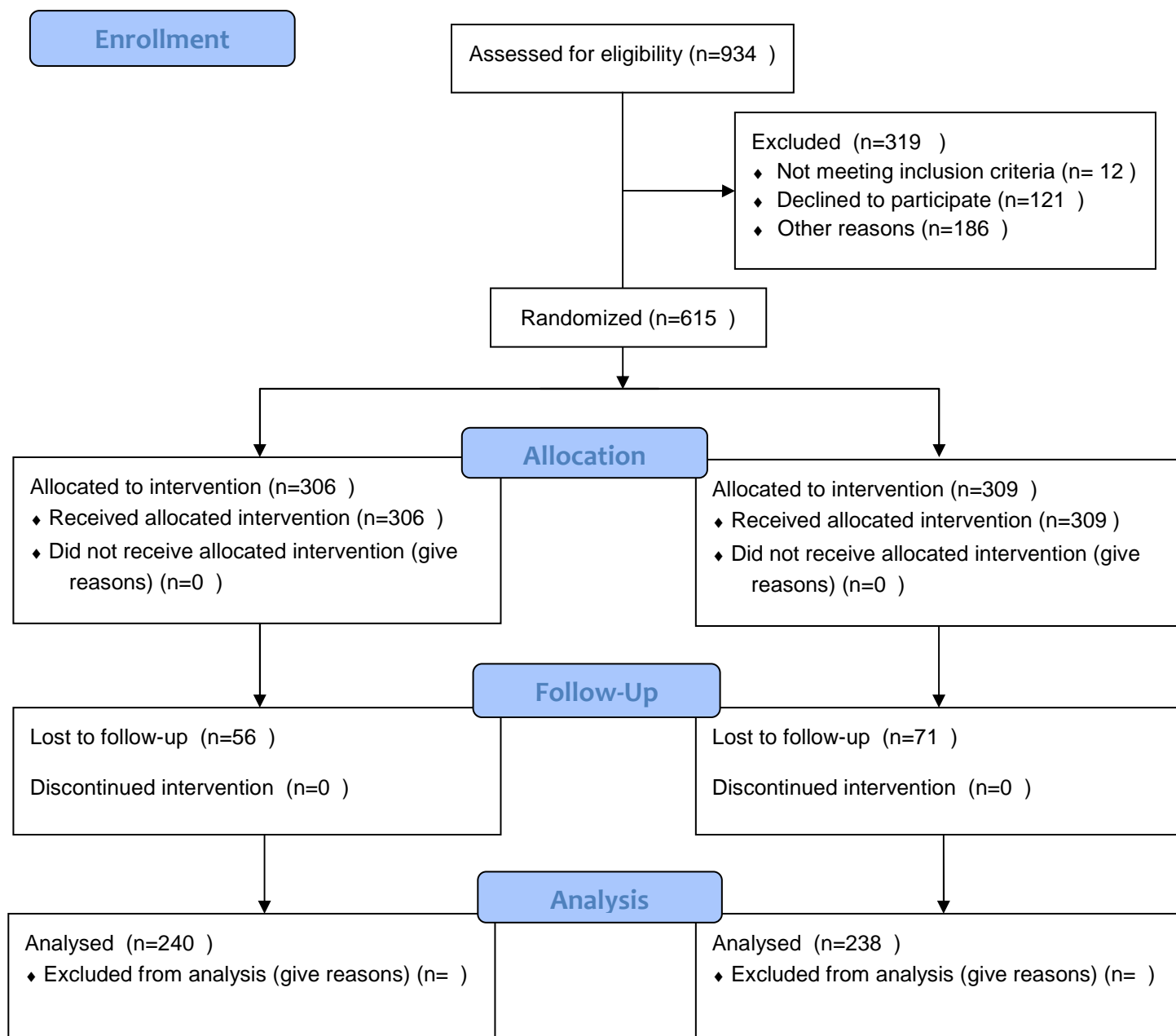

Supplement: Flowchart S1 — CONSORT 2010 Flow Diagram of the BARTrial. (PDF) [file pone.0099466.s007.pdf]
