# Supplementary material for: The Bilirubin Albumin Ratio in the Management of Hyperbilirubinemia in Preterm Infants to Improve Neurodevelopmental Outcome: A Randomized Controlled Trial – BARTrial
Source: PLoS One. 2014 Jun 13;9(6):e99466. doi: 10.1371/journal.pone.0099466 (PMC4057208; doi:10.1371/journal.pone.0099466)
Supplement: Protocol S1 — Study protocols of the BARTrial. (PDF) [file pone.0099466.s008.pdf]

This supplement contains the following items:

1. Original protocol, final protocol, summary of changes.
2. Original statistical analysis plan, final statistical analysis plan, summary of changes.

In short, the summary of changes consists of :

1. Replacement of the 2nd version of the Bayley Scales of Infant Development (BSID II) by the 3rd version (BSID III).

2. Subgroup analysis based on birth weight categories (< 1000 g vs. ≥ 1000 g) rather than an analysis based on gestational age categories (< 29 wks vs. ≥ 29 wks). This was prompted by the publication of the results of the aggresive-conservative phototherapy trial (Morris BH et al., N Engl J Med. 2008 Oct 30;359(18):1885-96).

Changes can be found in the second version of our study protocol (pp 42-81, yellow lines).

RESEARCH PROTOCOL

Reducing Bilirubin-Induced Neurological Dysfunction in Premature Newborns, Additional Use of the Bilirubin:Albumin Ratio in the Treatment of Hyperbilirubinemia

(December 2006)

(TEMPLATE VERSION Oct 2006)

PROTOCOL TITLE: 'Reducing bilirubin-induced neurological dysfunction in premature newborns, additional use of the bilirubin:albumin ratio in the treatment of hyperbilirubinemia '

Protocol ID	ABR 14881 – METc 2006/261
Short title	Reducing BIND in preterm infants
Version - Date	2 - December 22 2006
Co-ordinating investigator/project leader	Dr. Peter H. Dijk & Dr. Christian V. Hulzebos<br>Beatrix Children's Hospital<br>University Medical Centre Groningen<br>Hanzeplein 1<br>P.O. Box 30.001<br>9700 RB GRONINGEN The Netherlands<br>Tel 050 – 3614215
Project group	Dr. Peter H. Dijk: UMC Groningen<br>Dr. C.V. Hulzebos: UMC Groningen<br>Prof dr A.F. Bos: UMC Groningen<br>Dr. P. Govaert: ErasmusMC Rotterdam<br>Dr. M.J.L.N. Benders: UMC Utrecht<br>Prof dr H.J. Verkade: UMC Groningen<br>Dr. E.M. ter Vergert: MTA UMC Groningen
Principal investigators per site	UMC Groningen: Dr. P.H. Dijk & Dr. C.V. Hulzebos<br>Erasmus Rotterdam: Dr. P. Govaert<br>Amsterdam AMC: Dr. L. van Toledo<br>Amsterdam VU: Prof. dr. W.P.F. Fetter<br>UMG Utrecht: Dr. M. Benders<br>UMC Nijmegen: Dr. K.D. Liem<br>Leiden UMC: Prof. dr. F.J. Walther<br>AZ Maastricht: Prof. dr. J.L.I Zimmermann<br>MMC Veldhoven: Dr. J. Buijs<br>Isala Zwolle: Dr. R.A. van Lingen
Independent physician	Dr. Margriet S. van Stuijvenberg<br>Beatrix Children's Hospital<br>UMC Groningen<br>Hanzeplein 1<br>P.O. Box 30.001<br>9700 RB GRONINGEN<br>tel 050 – 3614215

PROTOCOL SIGNATURE SHEET

Name	Signature	Date
Legal representative<br>Head of Department:	Prof dr A.F. Bos<br>Head of Departement of Neonatology<br>Beatrix Childrens Hospital<br>UMC Groningen<br><br>………………………………	Dec 22 2006
Coordinating Investigators	Dr. P.H. Dijk – neonatologist<br><br>……………………………….<br><br>&<br>Dr. C.V. Hulzebos – neonatologist<br><br>………………………………….	Dec 22 2006<br><br>Dec 22 2006

TABLE OF CONTENTS

1. INTRODUCTION AND RATIONALE 10
2. OBJECTIVES 13
3. STUDY DESIGN 13
4. STUDY POPULATION 14
4.1 Population (base) 14
4.2 Inclusion criteria 14
4.3 Exclusion criteria 14
4.4 Sample size calculation 14
5. TREATMENT OF SUBJECTS 15
5.1 Investigational product/treatment 15
5.2 Use of co-intervention (if applicable) 15
5.3 Escape medication (if applicable) 15
6. METHODS 16
6.1 Study parameters/endpoints 16
6.1.1 Main study parameter/endpoint 16
6.1.2 Secondary study parameters/endpoints (if applicable) 16
6.1.3 Other study parameters (if applicable) 16
6.2 Randomisation, blinding and treatment allocation 16
6.3 Study procedures 17
6.4 Withdrawal of individual subjects 17
6.4.1 Specific criteria for withdrawal (if applicable) 17
6.5 Replacement of individual subjects after withdrawal 17
6.6 Follow-up of subjects withdrawn from treatment 18
6.7 Premature termination of the study 18
7. ECONOMIC EVALUATION 19
8. SAFETY REPORTING 20
8.1 Section 10 WMO event 20
8.2 Adverse and serious adverse events 20
8.2.1 Suspected unexpected serious adverse reactions (SUSAR) 20
8.2.2 Annual safety report 20
8.3 Follow-up of adverse events 20
8.4 Data Safety Monitoring Board (DSMB) 20
9. STATISTICAL ANALYSIS 21
9.1 Descriptive statistics 21
9.2 Univariate analysis 21
9.3 Multivariate analysis 21

9.4 Interim analysis (not applicable) 21
10. ETHICAL CONSIDERATIONS 22
10.1 Regulation statement 22
10.2 Recruitment and consent 22
10.3 Objection by minors or incapacitated subjects (if applicable) 22
10.4 Benefits and risks assessment, group relatedness 22
10.5 Compensation for injury 23
10.6 Incentives (if applicable) 23
11. ADMINISTRATIVE ASPECTS AND PUBLICATION 24
11.1 Handling and storage of data and documents 24
11.2 Amendments 24
11.3 Annual progress report 24
11.4 End of study report 24
11.5 Public disclosure and publication policy 24
12. REFERENCES 25
SUPPLEMENTS 26
Study guidelines 27
Patient information letter (patienteninformatiebrief) 28
Informed consent (toestemmingsverklaring) 33
Systematic Review 34

LIST OF ABBREVIATIONS AND RELEVANT DEFINITIONS

AAP	American Academy of Pediatrics
ABR	Auditory Brainstem Evoked Responses
ABR	ABR form (General Assessment and Registration form) is the application form that is required for submission to the accredited Ethics Committee (ABR = Algemene Beoordeling en Registratie)
AE	Adverse Event
AR	Adverse Reaction
AUC	Area Under the Curve
B:A ratio	Bilirubin:Albumin ratio
Bf	Free Unbound Bilirubin
BIND	Bilirubin-Induced Neurological Dysfunction
BSID	Bayley Scales of Infant Development
BPD	Bronchopulmonary dysplasia
CA	Competent Authority
CCMO	Centrale Commissie Mensgebonden Onderzoek
CCMO	Central Committee on Research Involving Human Subjects
CRF	Case Record Form
CV	Curriculum Vitae
DOL	Day Of Life
DSMB	Data Safety Monitoring Board
EU	European Union
EudraCT	European drug regulatory affairs Clinical Trials
GA	Gestational Age
GCP	Good Clinincal Practice
IB	Investigator's Brochure
IC	Informed Consent
IMP	Investigational Medicinal Product
IMPD	Investigational Medicinal Product Dossier
IVH	Intraventricular Hemorrhage
MDI	Mental Developmental Index
METC	Medical research ethics committee (MREC); in Dutch: medisch ethische toetsing commissie (METC)
NEC	Necrotising Enterocolitis
NICHD	National Institute of Child Health and Human Development
NICU	Neonatal Intensive Care Unit
NVK	Nederlandse Vereniging voor Kindergeneeskunde
PDA	Patent Ductus Arteriosus
PDI	Psychomotor Developmental Index

PMA	Postmenstrual Age
PVE	Periventricular Echodensity
PVL	Periventricular Leucomalacia
ROP	Retinopathy of Prematurity
(S)AE	Serious Adverse Event
SD	Standard Deviation
SPC	Summary of Product Characteristics (in Dutch: officiële productinfomatie IB1-tekst)
SUSAR	Suspected Unexpected Serious Adverse Reaction
TSB	Total Serum Bilirubin
Wbp	Personal Data Protection Act (in Dutch: Wet Bescherming Persoonsgevens)
WMO	Medical Research Involving Human Subjects Act (Wet Medisch-wetenschappelijk Onderzoek met mensen

SUMMARY

Background

Neonatal jaundice due to unconjugated hyperbilirubinemia occurs in almost all preterm infants and is potentially neurotoxic. Treatment is based on total serum bilirubin (TSB), but is not evidence based. TSB is an unreliable predictor of bilirubin induced neurological dysfunction (BIND). Low albumin levels increase free bilirubin (Bf) levels and may potentiate BIND. The bilirubin:albumin ratio is an interesting additional method in the management of hyperbilirubinemia.

Objective

The research question of this study is whether BIND is reduced using B:A ratio together with TSB versus TSB-only as indicator for treatment of hyperbilirubinemia in preterm infants.

Study design

Prospective, randomized, controlled, open label, blinded outcome cost-effectiveness multicenter study in tertiary neonatal intensive care units in the Netherlands.

Study population

Preterm infants born at a gestational age of less than 32 weeks postmentrual age.

Intervention

Hyperbilirubinemia is evaluated daily using the B:A ratio together with TSB (study group) *versus* TSB only (control group – care as usual). Treatment guidelines are based on B:A ratio and TSB (whichever comes first) in the study group *versus* on TSB-only in control group.

Main study parameters/endpoints

The primary outcome variables are the neurodevelopmental index scores at 18-24 months of age, using a standardised neurological examination and mental- and psychomotor developmental index scores (MDI and PDI).
Secondary outcome variables are: peak TSB, peak B:A ratio, duration of hyperbilirubinemia, number and duration of phototherapy, number of exchange transfusion, and all the "complications of prematurity" such as mortality, respiratory distress, bronchopulmonary dysplasia (BPD), retinopathy of prematurity (ROP), patent ductus arteriosus (PDA), necrotising enterocolitis (NEC), intraventricular hemorrhage (IVH), periventricular echodensitity (PVE) and periventricular leucomalacia (PVL).
Economic evaluations are part of this study. Costs-effectiveness assessments will be performed from a hospital perspective with a time-horizon of 18-24 months. Costs of diagnostics and treatments will be included. Long-term effects of neurological impairment and developmental delay on costs are estimated using modelling techniques.

Nature and extent of the burden and risks associated with participation, benefit and group relatedness:
Daily laboratory examinations will include bilirubin and albumin levels for the first ten days of life in both study groups . Usually, these measurements do not require extra blood volume. Sometimes, more blood is required (maximal 10 x 0.1 ml = 1.0 ml), but no extra puncture will be necessary. It is possible that more children will be treated with phototherapy and/or exchange transfusion in the study group in comparison to the care-as-usual protocols On the other hand, an earlier start phototherapy may reduce TSB-levels and prevent exchange transfusions. The net effect cannot be predicted.
A part of the study-population (i.e. infants admitted to the NICU of the UMC Groningen) will be subjected to a more extended audiological evaluation (ABR) during the first 10 days of life , than in the routine protocol for NICU patients (Algo-screening). In analogy of the Algo-screening, the ABR consists of auditory stimuli by headphone, registered by adhesive head-electrodes (3 instead of 1). Full developmental tests are included of this study protocol. These tests are part of the standard follow-up visits of NICU-graduates (care as usual).

Time schedule

Anticipated start of this study is March 2007. Inclusion period of 9 months. Follow-up at 18-24 months of age. Total duration of study is 36 months. End of study is March 2010.

Financial support

This study is financed by grant nr 945-07-407 of the "Doelmatigheidsonderzoek" program DO 07 of the ZonMw.

1. INTRODUCTION AND RATIONALE

Problem definition

Unconjugated hyperbilirubinemia occurs in nearly all preterm infants (Watchko 1992). Free bilirubin, the fraction not bound to albumin, is toxic for the central nervous system (Shapiro 2003). Free bilirubin is difficult to measure and routine clinical laboratory measurement is not available. Therefore, the TSB level is used to evaluate hyperbilirubinemia, but is not a good predictor of neurotoxicity. Follow-up studies that have evaluated the association between TSB and adverse outcome are inconclusive, and most failed (Van de Bor 1989 and 1992, Watchko 2003, Oh 2003). Preterm newborns are at greater risk for BIND than term newborns, but precise TSB levels at which treatment should be initiated to prevent neurological damage are not known. In addition, preterm newborns do not exhibit acute neurologic clinical signs of bilirubin encephalopathy seen in term newborns (Amin 2001,Govaert 2003). It is difficult to determine whether a preterm infant is at risk for bilirubin neurotoxicity, and no evidence-based management guidelines for preterm infants with hyperbilirubinemia exist. The aim of treatment is to prevent BIND while not causing harm. Phototherapy and exchange transfusions are effective and relatively safe in term newborns. Preterm newborns are much more likely to experience complications of exchange transfusions, such as arrhythmia, thrombosis, thrombocytopenia, necrotising enterocolitis, infections or death (Maisels 2003). Phototherapy is effective in the prevention for the need of exchange transfusions and side-effects are considered rare (Maisels 2003).
In summary, it is a healthcare problem that despite effective treatment strategies for hyperbilirubinemia, BIND still occurs in preterm infants.

Disease subject in this study

Hyperbilirubinemia and BIND

Subgroup of patients

Preterm infants < 32 weeks of gestational age.
Variation in sex, age and cultural background: Bilirubin production, metabolism and neurotoxicity may be influenced by gestational age, sex, race, feeding and possible risk factors for bilirubin neurotoxicity such as asphyxia, acidosis, sepsis, hemolysis and intracranial bleeding. Subgroup and multivariate analyses will be performed.

Usual care in the Netherlands

The American Academy of Pediatrics (AAP 2004) has recently published guidelines for the management of hyperbilirubinemia in term newborns based on TSB levels. This guideline will be adapted for the Netherlands supported by the Nederlandse Vereniging voor Kindergeneeskunde (NVK). For preterm infants no general accepted guidelines for the management of hyperbilirubinemia are available. Therefore, the usual care is not uniform. Also in the Netherlands several treatment schemes are in use, often adopted from text-books in neonatology. The schemes use gestational age (or birthweight), postnatal age and TSB, and reflect TSB thresholds at which phototherapy is initiated and higher levels at which exchange transfusion is recommended.

Who are involved in the usual care

Preterm infants born at GA less than 32 weeks are treated in 10 neonatal intensive care units in the Netherlands. Neonatologists and pediatricians are responsible for the medical care.

Motivation of the chosen intervention

Bilirubin neurotoxicity is mainly determined by free bilirubin, which is very difficult to measure. The B:A ratio is an estimate of free bilirubin and might therefore be a better indicator for a harmful bilirubin level than TSB. High levels of free bilirubin have been associated with BIND in preterm infants (Cashore 1982, Nakamura 1992). The B:A ratio correlates with free bilirubin and, has been used in determining the need for exchange transfusion (Ahlfors 1994). In addition, raised free bilirubin concentrations are more closely associated than TSB levels with signs of acute neurotoxicity in auditory brainstem evoked responses (ABR) (Amin 2001). There are no contemporary long-term studies in preterm infants relating free bilirubin levels and acute neurotoxic signs to long-term neurodevelopmental outcome.

We propose to investigate the use of B:A ratio along with TSB vs TSB only as markers of bilirubin toxicity and as intervention markers. The intervention thresholds are based on those published by Maisels 2003 and Ahlfors 1994. The B:A ratios are derived from TSB (umol/L) divided by 25 g/L for infants < 1250g and divided by 30 g/L for infants > 1250 g, which are albumin levels considered to be safe with regard to bilirubin-binding. Lower albumin levels result in higher B:A ratios that correspond with increased free bilirubin levels. The B:A thresholds are also based on the recommendations of Maisels 2003 and Ahlfors 2003.

We chose to investigate the effect on neurodevelopmental outcome at 18-24 months of age because it is one of the most relevant outcomes in neonatology (Shapiro 2005). We intend additional neurodevelopmental follow-up assessments at later ages, but those are beyond the scope of this study protocol.

Relevance

This study may contribute to the resolution of the healthcare problem in several ways. First, this study will elucidate whether the B:A ratio is an additional marker to TSB in predicting acute and chronic neurotoxicity causing neurodevelopmental impairment in preterm infants. Therefore neurotoxicity can be prevented, and the neurodevelopmental outcome of vulnerable preterm infants might improve.

Second, this multicenter study can provide evidence on the proposed management guidelines and uniform the wide range of currently used guidelines in the management of hyperbilirubinemia in preterm infants. Several foreign experts in the field state that our study could provide novel information on this topic.

Other studies related to this healthcare problem

In the USA a NICHD Neonatal Research Network randomised control study in preterm infants comparing conservative versus prophylactic i.e. aggressive use of phototherapy and exchange transfusion is underway. The main healthcare problem of bilirubin neurotoxicity in preterm infants is the same, but research questions and the chosen intervention are different from our study.

Reports by advisory boards on this subject

The American Academy of Pediatrics (AAP 2004) and the National Institute of Child Health and Human Development (Blackmon 2004) have called for further research into the clinical use of bilirubin – albumin relation to improve the ability to determine those babies truly needing treatment. The NICHD called for development of rigorous validation of measures to detect and quantify early manifestations of bilirubin-induced brain injury and to confirm the time-course of such manifestations in response to interventions including: (amongst others) neurophysiologic testing, including ABR monitoring (AAP 2004). The current American Academy of Pediatrics (AAP

2004) guidelines for managing healthy jaundiced term and near term newborns recommends the use of the bilirubin/albumin ratio in addition to the total serum bilirubin.
This study is supported by the Kwaliteitsbureau of the Nederlandse Vereniging voor Kindergeneeskunde.

Incidence of the targeted (sub)population
In the Netherlands, each year about 1400 infants are born with a gestational age of less than 32 weeks. Almost all these infants are treated in the ten NICUs in the Netherlands. Visible jaundice is an almost universal feature in these preterm children. Therefore, the majority is treated with phototherapy for several days. Only a small number preterm infants (estimated at about 20-50 per year in NL) is treated with exchange transfusions for more severe hyperbilirubinemia.

Estimated potential effects on health from the interventions evaluated in this study
At 18-24 months of age 40% of the children who were born before 32 wks of GA have neurodevelopmental delay (Stoelhorst 2003). This delay is due to several variables during infancy, and bilirubin toxicity is probably one of them. The potential effects of using the B:A ratio in addition to the TSB-level versus the TSB only (usual care) as diagnostic determinant for starting (and stopping) treatment (phototherapy or exchange therapy) for hyperbilirubinemia are based on the assumption that the B:A ratio is more closely related to the potential toxic free bilirubin levels than the TSB is. In preterm infants with low albumin levels, who are more vulnerable to bilirubin toxicity, therapy will be started at relatively (related to albumin) low bilirubin levels. Therefore, therapy will be started in those infants that really need it .We expect that in the vulnerable group of infants with low levels of albumin, bilirubin neurotoxicity and consequently neurodevelopmental delay will be reduced.

Estimated potential effects on costs from the interventions evaluated in this study
We expect that with a more selective application of the treatment of hyperbilirubinemia we are able to reduce the number of infants with neurological damage. The ex-preterm infants with neurological damage need special treatment e.g. physiotherapy, speech-language-communication-training, hearing-aids, special education and medical check-ups. In the group infants who are more vulnerable to bilirubin toxicity, with relatively low levels of albumin, the number of phototherapy days will probably increase but it is anticipated that the neurological outcome will be better in comparison to the usual care. Although short-term costs might increase (albumin measurements and phototherapy), life-time costs as a result of neurological disabilities will be reduced.

2. OBJECTIVES

The primary objective of this study is to evaluate whether the combination of B:A ratio together with TSB is a better marker for a harmful unconjugated bilirubin level than TSB only. Using this combination of B:A ratio and TSB in the treatment of hyperbilirubinemia will lead to better treatment and reduce BIND.
The primary research question is therefore: Is BIND reduced by using the combination of B:A ratio and TSB *versus* TSB only as indicators for phototherapy and exchange transfusion for the treatment of hyperbilirubinemia in preterm infants (born at less than 32 weeks PMA) ?

3. STUDY DESIGN

Prospective randomized controlled open label, blinded endpoints multicenter study in the ten Neonatal Intensive Care Units in the Netherlands (see flowchart).

Flowchart

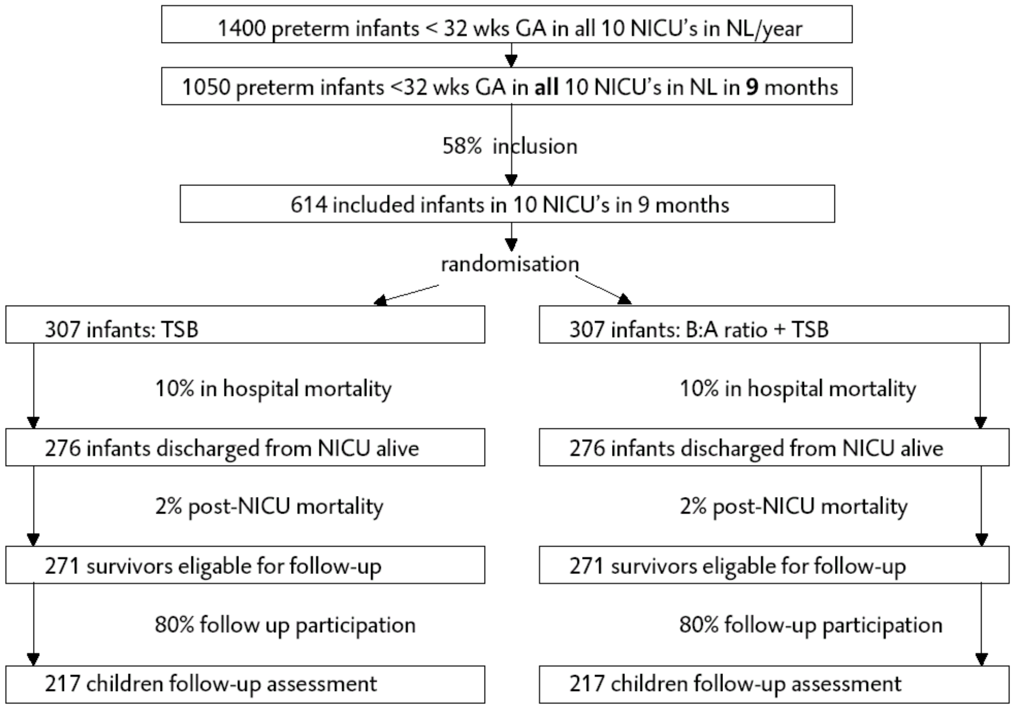

Poweranalysis: for a difference of 7 points in MDI/PDI (mean 96 +/- SD 26)
alpha 0.05 and beta 0.20, 2 x 217 subjects for assessment

4. STUDY POPULATION

4.1 Population (base)

All preterm infants born at less than 32 weeks of PMA treated in one of the ten NICU's in the Netherlands are eligible for this study.
In the Netherlands all infants born before 32 weeks of gestational age are treated in one of the ten NICU's. Non-NICU hospitals have an obligation to send mothers with imminent preterm delivery before 32 weeks of GA and the preterm infants born before 32 weeks PMA to the regional NICU. In the Netherlands each year about 1400 infants are born with a GA of less than 32 weeks. The majority of these infants are inborn (intrauterine transport of the infant). A minority of the children born before a gestational age of 32 weeks are transported postnatally, in their first day of life.

4.2 Inclusion criteria

- Prematurity (< 32+0 weeks of PMA)
- Admitted to a NICU in less than 24 hours after birth
- Informed consent of the parent or legal guardian

4.3 Exclusion criteria

Major congenital malformations, clinical syndromes and chromosomal abnormalities

4.4 Sample size calculation

Neurodevelopmental outcome at 18-24 months of age using mental – and psychomotor development scores (MDI and PDI) are assessed using the Dutch version of Bayley scales of infant development. A significant association has been reported between peak TSB concentration and PDI in a retrospective cohort analysis of the NICHD (Oh 2003). The PDI scale has a population mean of 100 and a standard deviation of 16, but a reference (i.e. in preterm infants <32 weeks) mean of 96 (SD 26) (Oh 2003, Stoelhorst 2003). Both scales range from 51 to 149. An MDI or PDI score of more than 85 is considered normal, a score between 68 and 84 is considered moderate delay and scores less than 68 as severe delay. A difference of 5 - 10 points in developmental score is considered relevant (personal communication Dijk/Hulzebos with all neonatologists in the Netherlands during a consensus meeting (Neoned Retraite in January 2006). With an alpha of 0.05 and power of 80% a change of 7 points on a 100 points scale, with a standard deviation of 26, 2x 217 subjects need to be assessed. If 10% mortality and 20% drop outs are considered in this population, a total of 2 x 307 subjects need to be included (INSTAT statistics, see flow chart).

5. TREATMENT OF SUBJECTS

5.1 Investigational treatment

This study compares two methods of management of hyperbilirubinemia in preterm infants. In the control group, the management of hyperbilirubinemia is based on TSB levels only (care as usual). The thresholds of treatment of hyperbilirubinemia with phototherapy or exchange transfusion are based on TSB only. In the study group a combination of TSB and B:A ratio is used. The thresholds of treatment (phototherapy and exchange transfusion) are based on both, TSB and B:A ratio, whichever comes first. Whenever one of the two markers of hyperbilirubinemia (TSB or B:A ratio) reaches the predefined threshold values, treatment should be started.

5.2 Use of co-intervention

The use of albumine infusion in the treatment of hyperbilirubinemia is discouraged in this study. Treatment with albumine infusion for other indications is not restricted. The study protocol has no restrictions with regard to co-interventions, co-medication or other specific guidelines. The study subjects are treated according to the guidelines of the individual NICU's.

5.3 Escape medication / treatment

Not applicable

6. METHODS

6.1 Study parameters/endpoints

6.1.1 Main study parameters/endpoints

The primary endpoint of this study is the neurodevelopmental outcome at the age of 18-24 months using standardised neurological examination and developmental scores: mental and psychomotor developmental indices (MDI and PDI) are assessed using the Dutch version of Bayley Scale of Infant Development II.

6.1.2 Secondary study parameters/endpoints

Secondary endpoints are bilirubin-related parameters with amongst others peak bilirubin levels, duration of hyperbilirubinemia, peak B:A ratio, number and duration of phototherapy, number of exchange transfusions.
Other secondary outcome parameters are those related to "the complications of prematurity" which are potential confounders for neurodevelopmental outcome. The complications of prematurity are mortality, respiratory distress syndrome (RDS), bronchopulmonary dysplasia (BPD), patent ductus arteriosus (PDA), necrotizing enterocolitis (NEC), retinopathy of prematurity (ROP), infections, intraventricular hemorrhage (IVH), periventricular echodensities (PVE) and periventricular leucomalacia (PVL).

6.1.3 Other study parameters/endpoints

Other study are potential intermediate variables in the evaluation of bilirubin metabolism and -toxicity. These parameters are free bilirubin levels, lumirubine and ABR-measurements.

Other study parameters are those involved in the economic evaluation or cost-effectiveness analyses and contain the direct and indirect costs of management of hyperbilirubinemia, prematurity and developmental delay.

It is the intention to perform additional neurodevelopment assessments at later ages (4-7years), but those are beyond the scope of this study protocol.

6.2 Randomisation, blinding and treatment allocation

Assessment for eligibility is performed by neonatologists and/residents of the participating NICU. They initially ask whether the parents will consider participation of their child and provide written patient information. The co-ordinating research nurses/investigators inform the parents in detail. A 24 hrs – 7 days schedule will be made up to be able to inform and enrol participants day and night, 7 days a week. The written information contains information on the objectives, design, methods, duration, possible advantages and disadvantages of the study treatments, and information that non-co-operation with the study or withdrawal will not have consequences for the treatment of their child. Research nurses/investigators obtain written informed consent and are available for additional information to the parents. On demand, parents can obtain additional information from an independent pediatrician. Parents are allowed to consider participation for several hours, with a maximum of 24 hours, since subjects need to bee included during their first day of life.
After informed consent has been obtained study subjects are randomised using a web-based computer scheme. The subjects are assigned in a 1:1 ratio to one of the two study groups. Randomisation is stratified according to the study site (NICU) and gestational age group (two groups: 24^{+0} - 28^{+6} weeks and 29^{+0} - 31^{+6}weeks) .
Blinded treatment assignment is not possible. Clinicians should be aware whether to apply the hyperbilirubinemia guidelines based on TSB (control group) or B:A ratio and/or TSB (study group). Blinding of the study subjects is not

an issue in preterm infants. The parents are not aware of group-allocation to secure blind assessment of the primary endpoint . The main study endpoints are assessed blindly. Neurodevelopmental outcome tests are performed by neonatologists and assistants who are unaware of the group allocation of the subjects.

6.3 Study procedures

In both study groups hyperbilirubinemia is evaluated daily for the first ten days of life. Treatment of hyperbilirubinemia will be based on total serum bilirubin in the care as usual group, whereas in the study group the combination of bilirubin:albumin ratio and total serum bilirubin will guide treatment. In both groups total serum bilirubin and albumin will be measured for post-hoc analyses purposes.

In general no extra blood volume is needed for these measurements, because these measurements are part of the daily routine blood examinations. In some cases this may cost several tenths of millilitres blood extra (maximal 10x0.1ml=1.0ml), but no extra punctures will be necessary.

The primary outcome is neurodevelopmental outcome by 18-24 months of age and consists of a standardised neurological physical examination and assessments of developmental scores: mental and psychomotor scores using the Dutch version of the Bayley scales of infant development (BSID-II-NL). The mental developmental index (MDI) assesses environmental responsiveness and sensory and perceptual abilities, memory, learning and early language and communication abilities. The psychomotor developmental index (PDI) assesses both gross and fine motor skills. Mental, motor and behaviour functions are tested by playing and observations. The test duration is about 60 min. Each child will undergo a standardised pediatric neurologic evaluation to assess the quality of their motor skills, co-ordination, gait and behaviour. Cerebral palsy is diagnosed with the use of standard criteria, including the location or body part impaired (e.g. hemiplegia or diplegia), the degree of impairment of muscle tone, and reflexes, and the effect of the condition on ambulation. Audiologic and visual impairment are tested by standardised tests.

Residual blood samples will be stored for free bilirubin measurements: no extra punctures will be done for this purpose.

Residual urine samples that are collected as part of the routine clinical treatment, will be stored for lumirubine measurements.

In a subgroup of children (i.e. infants admitted to the NICU of the UMC Groningen), serial automated brainstem evoked potentials (ABR) will be assessed using a earphone to apply sound-clicks and self-adhesive electrodes applied to the head to record the time that sound-stimuli need to reach the brain.

6.4 Withdrawal of individual subjects

Subjects can leave the study at any time for any reason if they (their parents/caretakers) wish to do so without any consequences. The investigator can decide to withdraw the subject from the study for urgent medical reasons.

6.4.1 Specific criteria for withdrawal

Specific criteria for withdrawal are those circumstances in which the exclusion criteria (major congenital malformations, clinical syndromes and chromosomal abnormalities) are being diagnosed after inclusion.

Death and other serious complications of prematurity are not a reason to withdraw subjects.

6.5 Replacement of individual subjects after withdrawal

The number of withdrawn subjects will be replaced.

6.6 Follow-up of subjects withdrawn from treatment

Subjects withdrawn from the study will be treated according to the usual care including neurodevelopmental outcome assessment which is part of the usual follow up of NICU-graduates.

6.7 Premature termination of the study

The primary outcome variable of this study is neurodevelopmental outcome at 18-24 months of age, which is beyond the timespan of the inclusion period of this study (9 months). Therefore, interim analyses or application of stopping rules during the inclusion period is not relevant. A Data Safety Monitoring Board will monitor the study population on safety aspects. This Board may advise the Project group to alter the study protocol or even terminate the study in case of (unexpected) serious adverse events. Please see chapter 8: “Safety Reporting”.

7. Economic evaluation (MTA)

This study includes an economic evaluation, and will be conducted as a cost-effectiveness analysis. Therefore, the costs of care for preterm infants that are evaluated using TSB versus B:A ratio along with TSB, will be compared by looking at the incremental costs per additional point on the PDI scale at 18-24 months. A more favourable score on the PDI scale is expected in the group treated according to the B:A ratio. The evaluation will be conducted from a hospital perspective including direct medical costs. Costs of diagnostics (bilirubin and albumin measurements) as well as treatment in case of unconjugated hyperbilirubinemia (phototherapy and exchange transfusions) will be taken into account. A case record form (CRF) will be used to collect costs for each patient during the study period. In addition, a decision-analytic model will be constructed to assess the long-term cost-effectiveness of prevention of bilirubin induced neurological damage. Costs of long-term effects of neurological impairments such as hospital stay, doctors-visits, hearing-aids, physiotherapy, and speech-therapy will be based on both retrospective data from our institutions and data from the literature (e.g. Io 2004, Hack 2000). The model will be built with Tree-Age Pro Healthcare Edition. In addition, uncertainty surrounding the incremental cost-effectiveness will be presented using a cost-effectiveness plane. Cost components will be vaulted according to the Dutch guidelines for economic evaluation (CVZ).

Time horizon of this study is 18-24 months for the prospective economic evaluation and lifetime for the analysis with the model. Due to a long time-horizon, discounting will be applied. A sensitivity analysis will be conducted to estimate the impact of variation of major cost elements as well as different discount rates.

8. SAFETY REPORTING

8.1 section 10 WMO event

In accordance to section 10, subsection 1, of the WMO, the investigator will inform the subjects (parents/caretakers) and the reviewing accredited METC if anything occurs, on the basis of which it appears that the disadvantages of participation may be significantly greater than was foreseen in the research protocol. The study will be suspended pending further review by the accredited METC, except insofar as suspension would jeopardise the subjects' health. The investigator will take care that all subjects are kept informed.

8.2 Adverse and serious events

This study is evaluating long-term consequences of diagnostic procedures on neurodevelopmental outcome and is not an investigational medical product study. Treatment modalities in this study protocol are equal to those applied in the usual care. It may be possible that more children will be treated with phototherapy and/or exchange transfusion in the study group in comparison to the care-as-usual protocols. Phototherapy and exchange transfusions are effective and relatively safe in term newborns. Preterm newborns are much more likely to experience complications of exchange transfusions, such as arrhythmia, thrombosis, thrombocytopenia, necrotising enterocolitis, infections or death (Maisels 2003). Phototherapy is effective in the prevention for the need of exchange transfusions and side-effects are considered rare (Maisels 2003). Therefore, earlier phototherapy may reduce TSB-levels and prevent exchange transfusions. The net effect cannot be predicted.
Other adverse and serious events are not to be expected and not related to the study protocol. Preterm infants treated in NICUs often suffer from serious effects. These complications of prematurity are recorded in the CRF and analysed continuously and monitored by the Data Safety Monitoring Board.

8.3 Follow-up of adverse events

All infants will participate in the usual NICU-graduate follow-up. This follow-up program is targeted at evaluating and coordinating diagnostic procedures and treatment of all prematurity related problems, in close cooperation with regional and local pediatricians.

8.4 Data Safety Monitoring Board

During the inclusion period of 9 months until 3 months thereafter the Data Safety Monitoring Board will analyse the incidence of the above mentioned complications of prematurity in both study groups. The Board will, as needed, review individual records to be able to analyse potential associations between complications and the study protocol. The Board may advise the Project Group to alter the study protocol or even terminate the study in case of serious adverse effect. The personal composition of the Data Safety Monitoring Board has to be determined.

9. STATISTICAL ANALYSES

9.1 Descriptive statistics

All randomised subjects will be analysed on the intention to treat basis. The unpaired two-sided Student's t-test will be used to compare the primary outcome data (i.e. PDI) in the study group versus the control group.
Categorical data will be compared using two-sided Chi-square tests with continuity correction or Fischer's exact test. Normally distributed continuous variables are compared using the unpaired Student's t-test. Nonparametric continuous variables are compared using the Wilcoxon rank sum test or Mann-Whitney U-test. Significance is defined as $p<0.05$. All variables are tested two-sided.

9.2 and 9.3 Univariate analysis & multivariate analysis

In post- hoc analyses logistic regression models will be applied to analyse the association between bilirubin variables (peak TSB, peak B:A ratio, duration of hyperbilirubinemia etc) and neurodevelopmental outcome variables. Demographic risk factors (e.g. gestational age, birthweight, sexe, mothers' educational level) and other confounders (complications of prematurity such as: intracranial hemorrhage, periventricular leucomalacia, postnatal steroids, necrotising enterocolitis, bronchopulmonary dysplasia, retinopathy of prematurity, late sepsis) will be included in multivariable logistic regression models.

9.4 Interim analysis

Interim analyses are not performed. This study is an long-term outcome study. The outcome assessment at the age of 18-24 months is beyond the end of the inclusion period (9 months). Therefore, it is not relevant to perform interim analyses on the primary outcome.

10. ETHICAL CONSIDERATIONS

10.1 Regulation statement

This study will be conducted according to the principles of the Declaration of Helsinki (version 9.10.2004) and in accordance with the Medical Research Involving Human Subjects Act (WMO).

10.2 Recruitment and consent

Assessment for eligibility is performed by neonatologists and/residents of the participating NICU's. They initially ask whether the parents would consider participation of their child and provide written patient information. The co-ordinating research nurses/investigators inform the parents in detail. A 24 hrs – 7 days schedule will be made up to be able to inform and enrol participants day and night, 7 days a week.. The written information contains information on the objectives, design, methods, duration, possible advantages and disadvantages of the study treatments, and information that non-co-operation with the study or withdrawal will not have consequences for the treatment of their child. Research nurses/investigators obtain written informed consent and are available for additional information to the parents. On demand, parents can obtain additional information from an independent pediatrician. Parents are allowed to consider participation for several hours, with a maximum of 24 hours, since subjects need to bee included during their first day of life. (please see patient information letter and informed consent letter)

10.3 Objection by minors or incapacitated subjects.

This study will be conducted according to the principles of the code of conduct for resistance in minors participating in medical scientific research (Gedragscode bij verzet van minderjarigen die deelnemen aan medisch-wetenschappelijk onderzoek), stated by the Dutch society of pediatrics: Nieuwsbrief Nederlandse Vereniging voor Kindergeneeskunde (NVK), nummer 3, 2001.

10. 4 Benefits and risks assessments, group relatedness

Neonatal jaundice due to unconjugated hyperbilirubinemia is a physiological phenomena of the newborn infant. Hyperbilirubinemia is potentially neurotoxic and related to psychomotor developmental impairment. Studies comparing different management strategies of hyperbilirubinemia in preterm infants assessing neurodevelopmental outcome can only be conducted in preterm infants. There are no other, less vulnerable, potential study subjects in which our research question can be studied. The main goal of this study is to reduce neurodevelopmental impairment in preterm infants. In the Netherlands about 40% of the ex-preterm NICU-graduates have delayed mental and psychomotor development. Therefore, individual subjects of the study population could benefit from this study, by a better neurodevelopmental outcome, if our hypothesis appears to be true.

To our opinion, the risks and hazard associated with participation to this study can be considered negligible and burden minimal. In the study group the B:A ratio is used as marker for potential neurotoxic hyperbilirubinemia, in addition to the total serum bilirubin (TSB). In the control group, in which the care as usual is applied, only the TSB level is taken into account. Therefore this study can be considered safe.

10.5 Compensation for injury

The sponsor/investigator has a liability insurance which is in accordance with article 7, subsection 6 of the WMO.

The sponsor (also) has an insurance which is in accordance with the legal requirements in the Netherlands (Article 7 WMO and the Measure regarding Compulsory Insurance for Clinical Research in Humans of 23rd June 2003). This insurance provides cover for damage to research subjects through injury or death caused by the study.

1. € 450.000,-- (i.e. four hundred and fifty thousand Euro) for death or injury for each subject who participates in the Research;
2. € 3.500.000,-- (i.e. three million five hundred thousand Euro) for death or injury for all subjects who participate in the Research;
3. € 5.000.000,-- (i.e. five million Euro) for the total damage incurred by the organisation for all damage disclosed by scientific research for the Sponsor as 'verrichter' in the meaning of said Act in each year of insurance coverage.

The insurance applies to the damage that becomes apparent during the study or within 4 years after the end of the study.

10.6 Incentives

Eligible participants do not receive any special incentives that may encourage participation in this study.

11. ADMINISTRATIVE ASPECTS AND PUBLICATION

11.1 Handling and storage of data and documents

Data are collected in digital patient record forms. Study data are handled confidentially and anonymously and in accordance with the Dutch personal data protection act (de wet bescherming persoonsgegevens). Subjects have unique identification codes (study number), that are not logically related to the personal data. The list of codes that link the study numbers to the individual subjects is safeguarded by the central investigator. Blood samples and study related data that are not directly linked to clinical patient management are stored using that study number.

11.2 Amendments

All substantial amendments will be notified to the METC of the UMC Groningen. Non-substantial amendments will not be notified to the accredited METC and the competent authority, but will be recorded and filed by the investigator.

11.3 Annual progress reports

The investigator will submit a summary of the progress of the study to the accredited METC once a year.
Information will be provided on the date of inclusion of the first subject, numbers of subjects included and numbers of subjects that have completed the trial, serious adverse events/ serious adverse reactions, other problems, and amendments.

11.4 End of study report

The investigator will notify the accredited METC of the end of the study within a period of 8 weeks. The end of the study is defined as the last patient's last visit. In case the study is ended prematurely, the investigator will notify the accredited METC, including the reasons for the premature termination.
Within one year after the end of the study, the investigator will submit a final study report with the results of the study, including any publications/abstracts of the study, to the accredited METC.

11.5 Public disclosure and publication policy

This clinical trial will be registered in a clinical trial register (Nederlands Trial Register at www.trialregister.nl). Papers for publication in scientific literature will be admitted on behalf of the "project group on hyperbilirubinemia in preterm infants". Members are the members of the scientific project group, one co-ordinating investigator of each participating centre, and MTA investigators.

12. REFERENCES

Watchko JF, Oski FA. Kernicterus in preterm newborns: past, present, and future. Pediatrics 1992; 90: 707-715.

Shapiro SM. Bilirubin toxicity in the developing nervous system. Pediatr Neurol 2003; 29: 410-421.

Van de Bor et al. Hyperbilirubinemia in preterm infants and neurodevelopmental outcome at 2 years of age. Pediatrics 1989;83:915-920.

Van de Bor et al. Hyperbilirubinemia in low birth weight infants and outcome at 5 years of age. Pediatrics 1992;89:359-364.

Watchko JF, Maisels MJ. Jaundice in low birth weight infants: pathobiology and outcome. Arch Dis Child Fetal Neonatal Ed 2003;88:F455-F458.

Oh W, Tyson JE, Fanaroff AA et al. Association between peak serum bilirubin and neurodevelopmental outcomes in extremely low birht weight infants. Pediatrics 2003;112:773-779.

Govaert P et al. Changes in globus pallidus with preterm kernicterus. Pediatrics 2003;11:1256-1263.

Amin SB, Ahlfors C, Orlando MS, Dalzell LE, Merle KS, Guillet R. Bilirubin and serial auditory brainstem responses in premature infants. Pediatrics 2001; 107: 664-670.

Maisels MJ, Watchko JF. Treatment of jaundice in low birthweight infants. Arch Dis Child Fetal Neonatal Ed 2003; 88: F459-F463.

AAP. Management of hyperbilirubinemia in the newborn infant 35 or more weeks of gestation. Pediatrics 2004; 114: 297-316.

Cashore WJ, Oh W. Unbound bilirubin and kernicterus in low birthweight infants. Pediatrics 1982;69:481-485.

Nakamura H, Yonetani M, Uetani Y et al. Determination of serum unbound bilirubin for prediction of kernicterus in low birth weight infants. Acta Paediatr Jpn 1992;54:642-647.

Ahlfors CE. Criteria for exchange transfusion in jaundiced newborns. Pediatrics 1994;93:488-494.

Shapiro SM. Definition of the clinical spectrum of kernicterus and BIND. J Perinatol 2005;25:54-59.

Blackmon et al. Research on prevention of bilirubin induced brain injury and kernicterus. Pediatrics 2004;114:229-233.

Stoelhorst et al. Developmental outcome at 18 and 24 month of age in very preterm infants. Early Hum Dev 2003;72:83-95

SUPPLEMENTS

Study guidelines
Patiënteninformatiebrief
Informed consent
Systematic Review

STUDY GUIDELINES

Reducing BIND in preterm infants ZonMw DO 2007

Table 1. Study guideline for use of phototherapy and exchange transfusion in preterm infants based on B:A ratio (µmol/l /g/l) and/or TSB (µmol/l), whichever comes first

Birthweight (g)	Phototherapy				Exchange transfusion			
	standard risk		high risk		standard risk		high risk	
	TSB	B:A	TSB	B:A	TSB	B:A	TSB	B:A
**<1250**	150	6.0	100	4.0	220	8.8	170	6.8
**1250 - 1499**	190	6.3	150	5.0	260	10.4	220	8.8
**1500 - 2000**	220	7.3	190	6.3	290	11.6	260	10.4
**2000 - 2500**	240	8.0	220	7.3	310	12.4	290	11.6

Guideline for the use of phototherapy and exchange transfusion in preterm infants based on TSB and B:A ratio, whichever comes first. TSB in µmol/l and albumin in g/l, B:A ratio in µmol/l/g/l = µmol/g.
Phototherapy is initiated whenever one of the thresholds is reached. Phototherapy is stopped whenever both, B:A ratio and TSB levels are under the thresholds. Exchange transfusion is considered whenever the thresholds are reached despite intensive phototherapy. Phototherapy thresholds are derived from exchange transfusion threshold minus 4 mg/dl = 70 µmol/l.

high risk:

- asfyxia: apgar score < 3 at 5 minutes
- hypoxemia: for more than 2 hours PaO2 < 40 mmHg (< 5.3 kPa) in recent 24 hours
- acidosis: for more than 1 hour pH < 7.15 in recent 24 hours
- birthweight < 1000 gram
- hemolysis: positive coombs reaction
- clinical or central nervous system deterioration: sepsis needing vasopressors, intracranial heamorrhage, meningitis.

based on guidelines provided by Ahlfors 1994 and Maisels 2003

Table 2. Study guideline for use of phototherapy and exchange transfusion in preterm infants based on TSB (µmol/l) only

Birthweight	Phototherapy		Exchange transfusion	
	standard risk	high risk	standard risk	high risk
	TSB	*TSB*	*TSB*	*TSB*
**<1250**	150	100	220	170
**1250 - 1499**	190	150	260	220
**1500 - 2000**	220	190	290	260
**2000 - 2500**	240	220	310	290

Guideline for the use of phototherapy and exchange transfusion in preterm infants based on TSB (in µmol/l).
Phototherapy is initiated whenever the threshold is reached. Phototherapy is stopped whenever TSB is under the threshold. Exchange transfusion is considered whenever the threshold is reached despite intensive phototherapy.

high risk:

- asfyxia: apgar score < 3 at 5 minutes
- hypoxemia: for more than 2 hours PaO2 < 40 mmHg (< 5.3 kPa) in recent 24 hours
- acidosis: for more than 1 hour pH < 7.15 in recent 24 hours
- birthweight < 1000 gram
- hemolysis: positive coombs reaction
- clinical or central nervous system deterioration: sepsis needing vasopressors, intracranial heamorrhage, meningitis.

based on guidelines provided by Ahlfors 1994 and Maisels 2003

Study guidelines December 2006

PATIENTEN INFORMATIE EN INFORMED CONSENT

Reducing BIND in preterm Infants ABR 14881 METc 2006/261 December 2006

ONDERZOEK NAAR HYPERBILIRUBINEMIE BIJ PREMATUREN

Reducing bilirubin induced neurological dysfunction in preterm infants: Additional use of bilirubin:albumin ratio in the treatment of hyperbilirubinemia.

-Het reduceren van bilirubine geïnduceerde neurologische schade bij te vroeg geboren kinderen: additioneel gebruik van de bilirubine: albumine ratio voor de behandeling van hyperbilirubinemie -

INFORMATIE TEN BEHOEVE VAN OUDERS VAN DEELNEMERS

Geachte mevrouw/mijnheer,

Uw kind is in onze kliniek onder behandeling omdat hij/zij te vroeg geboren is. Zoals in alle academische ziekenhuizen wordt ook in het UMC Groningen medisch-wetenschappelijk onderzoek gedaan om de zorg te verbeteren. Dergelijk onderzoek is alleen mogelijk met de medewerking van patiënten. Wij willen u hierbij vragen om toestemming om ook uw kind in een onderzoek te betrekken. In deze brief willen wij u informatie geven over het doel van het onderzoek waarvoor uw kind in aanmerking komt, de onderzoeks-procedure en de voor- en nadelen ervan. Deelname aan het onderzoek is vrijwillig. Als u deze informatie gelezen hebt en hierover nog vragen hebt kunt u die met uw arts bespreken. Als u vindt dat u voldoende informatie hebt kunt u beslissen of u aan het onderzoek wilt deelnemen.

Wetenschappelijk onderzoek

Zoals u waarschijnlijk weet kunnen alle pasgeborenen enkele dagen na de geboorte een gele kleur krijgen. Dit wordt veroorzaakt door een gele kleurstof (bilirubine). Het bilirubine is een afvalstof van de rode bloedcellen. Kort na de geboorte is de lever nog niet rijp genoeg om de gele bilirubine op te ruimen en blijft er dus meer bilirubine in het bloed. Bilirubine kan vanuit het bloed in de huid komen en geeft de huid de gele kleur. Bij kinderen die te vroeg geboren zijn is de aanmaak van bilirubine hoger en is de lever nog minder rijp. Daarom worden te vroeg geboren kinderen nog vaker dan op tijd geboren kinderen geel. Het bloed bevat dan veel bilirubine. Dit heet hyperbilirubinemie. Een te hoog gehalte van bilirubine kan schadelijk zijn voor de hersenen. Wanneer het bilirubine gehalte boven een bepaalde grenswaarde komt, zal een behandeling gestart moeten worden die erop gericht is het bilirubinegehalte te laten dalen. De behandeling bestaat uit lichttherapie (fototherapie), waarbij blauwe licht dat op de huid schijnt, bilirubine afbreekt tot stoffen, die gemakkelijk uit het lichaam verwijderd kunnen worden. Dit duurt enige tijd. Soms is het gehalte bilirubine in het bloed zo hoog dat we daar niet op kunnen wachten en moet het gele bloed gewisseld worden met ander bloed om snel het bilirubine gehalte te laten dalen. Deze wisseltherapie is een meer ingrijpende behandeling die ook tot complicaties kan leiden.
De huidige behandeling is gebaseerd op het totale bilirubinegehalte. Wij willen onderzoeken of de behandeling van hyperbilirubine nog beter kan door de behandeling niet alleen van het bilirubine, maar ook van het albumine eiwit af te laten hangen. In het bloed wordt namelijk het grootste deel van het bilirubine gebonden oftewel vastgehouden door dit eiwit (albumine). Vooral het niet aan albumine gebonden bilirubine (vrije bilirubine) kan vanuit het bloed in de hersenen komen. Het aan albumine gebonden bilirubine kan dat niet. Een laag albumine gehalte kan dus betekenen dat er meer vrij bilirubine is met daardoor een toegenomen kans op hersenschade.
De vraag is of de schadelijke effecten van bilirubine op de ontwikkeling van kinderen kan worden voorkomen door de behandeling te baseren op een combinatie van bilirubine en albumine. Om dit te

bestuderen wordt in ons ziekenhuis een wetenschappelijk onderzoek uitgevoerd. Naast ons ziekenhuis doen ook andere ziekenhuizen in Nederland mee. In totaal zullen ruim 600 kinderen aan het onderzoek mee gaan doen.

Het onderzoek waarvoor uw deelname wordt gevraagd

In het onderzoek wordt bestudeerd of de behandeling van te vroeg geboren babies met een hoog bilirubine gehalte door het meten van het eiwit albumine in het bloed, naast het bilirubine gehalte mogelijke negatieve effecten op de ontwikkeling van de kinderen worden voorkomen. De kinderen die aan het onderzoek meedoen, worden door de computer in twee groepen verdeeld. De ene groep wordt behandeld op de manier zoals we dat gebruikelijk doen, dus behandeling op basis van het totale gehalte van bilirubine in het bloed. Bij de andere groep wordt naast het totale gehalte van bilirubine, het albumine gehalte meegenomen in de beoordeling of behandeling met blauw licht of wisseltherapie gestart moet worden. Op twee jarige leeftijd zal de ontwikkeling van de kinderen getest worden. Door de resultaten onderling te vergelijken kunnen we nagaan wat het beste is.

De gang van zaken tijdens het onderzoek

Bij beide groepen kinderen zullen we de eerste tien levensdagen het bilirubine- en albumine gehalte in het bloed bepalen. Het onderzoek duurt in principe 10 dagen. Als u kind langer geel blijft zal het onderzoek zolang duren totdat die geelheid verdwenen is. Daarna zal op tweejarige leeftijd de ontwikkeling van uw kind worden getest en een uitgebreid lichamelijk onderzoek worden gedaan. Naast de standaard gehoorscreening zal er in de eerste 10 levensdagen een uitgebreider gehoors-onderzoek (afgekort: ABR) plaatsvinden, waarbij geluidsklikjes via een koptelefoontje worden gegeven en via 3 plakelectroden op het hoofd wordt gemeten of het geluid goed aankomt.
De totale duur van het hele onderzoek is 3 jaar. Na die periode vergelijken we de resultaten van alle kinderen die aan het onderzoek hebben meegedaan en kunnen we zien wat de beste manier van behandelen is.

Wat betekent meedoen voor uw kind ?

De eerste tien levensdagen zal er dagelijks bloed worden onderzocht op bilirubine en albumine. Als het bilirubinegehalte te hoog is zal behandeling worden gestart in beide groepen. In de tweede groep wordt ook gekeken naar het albumine gehalte. Mocht dat relatief laag zijn dan zullen we eerder beginnen met de behandeling. De keuze op welke van de twee wijzen uw kind behandeld wordt, wordt door de computer gedaan. U en wij mogen dus niet kiezen, zodat de kinderen zo eerlijk mogelijk over beide groepen verdeeld worden. We mogen u dus ook niet vertellen in welke groep uw kind terrecht is gekomen, zodat zowel u en wij niet bevooroordeeld zijn als de ontwikkelingstests worden gedaan.
Bij alle kinderen die voor de 32^{e} zwangerschapsweek geboren zijn wordt de eerste levensdagen vaak dagelijks bloedonderzoek verricht. Dit is nodig voor de gebruikelijke behandelingen. Meestal zal dan ook het bilirubinegehalte een aantal keren worden bepaald. Voor dit onderzoek zal het bilirubine gehalte dagelijks worden bepaald. Het bepalen van het eiwit albumine is extra. Daarvoor is echter meestal geen extra bloed afname nodig. Restanten van bloedmonsters en urinemonsters zullen bewaard blijven zodat we daarin stoffen (vrij bilirubine en lumirubine) kunnen meten die te maken hebben met de bilirubine stofwisseling.
Er zal als uw kind 18-24 maanden oud is, bij de gebruikelijke polikliniek controle met aandacht voor groei en ontwikkeling van uw kind, een uitgebreide ontwikkelingstest worden gedaan. Ook de artsen en assistenten op de polikliniek weten niet in welke onderzoeksgroep uw kind is geweest, zodat zij niet bevooroordeeld zijn. De poliklinIekbezoeken (follow-up) zijn standaard voor alle kinderen die te vroeg

geboren zijn, dus ook voor de kinderen die niet meedoen aan het onderzoek. De ontwikkelingstest is extra in verband met het onderzoek, het gebeurd spelenderwijs en duurt ongeveer 3 uur.
Het is mogelijk dat we op latere leeftijd (4-7 jaar) nogmaals een uitgebreide ontwikkelingstest zouden willen doen, maar dat is nog geen onderdeel van deze studie. Misschien benaderen we u later of u daaraan zou willen meedoen. Wij vragen nu alvast uw toestemming hiervoor.

Bijwerkingen, voor- en nadelen

Voor dit onderzoek hoeft u kind geen extra prikken te ondergaan en meestal hoeft er ook niet meer bloed te worden afgenomen dan voor de gebruikelijke behandeling nodig is. In sommige gevallen moet er wel iets meer bloed worden afgenomen (0.1 ml). Daarnaast is het mogelijk dat uw kind eerder en/of langer blauw lichttherapie krijgt in verband met de hyperbilirubinemie. Uw kind zou mogelijk eerder in aanmerking komen voor wisseltherapie, maar het kan ook zijn dat door het eerder starten van de blauw lichttherapie juist wisseltherapie voorkomen kan worden. Het effect daarop weten we niet goed.

Bedenktijd

Natuurlijk zult u tijd nodig hebben om erover na te denken of u aan dit onderzoek wilt meewerken. Hiervoor krijgt u uiteraard de gelegenheid. Omdat de studie op de eerste levensdag moet beginnen is de maximale bedenktijd 24 uur. Mocht er iets niet geheel duidelijk zijn dan willen wij u dat graag uitleggen.

Vertrouwelijkheid van de gegevens

De gegevens die voor dit onderzoek over uw kind verzameld worden, zullen vertrouwelijk worden behandeld. De gegevens worden op aparte formulieren ingevuld, waarop alleen een nummer voorkomt, niet de naam en persoonlijke gegevens. De gegevens worden dus anoniem verwerkt. In publicaties zal de naam van uw kind niet terug te vinden zijn.
Het is van groot belang dat de resultaten van het onderzoek juist worden weergegeven. Daarom worden al tijdens het onderzoek de gegevens gecontroleerd. Hiervoor zal tijdens het onderzoek de verzamelde gegevens door een onafhankelijke waarnemer worden vergeleken met de gegevens in het medische dossier van uw kind. De leider van het onderzoek is er verantwoordelijk voor dat op vertrouwelijke wijze met die gegevens wordt omgegaan. Tenslotte bestaat er de mogelijkheid dat de Inspectie voor de Volksgezondheid, als officiële instantie, de gang van zaken rond het onderzoek komt inspecteren.

Verzekering

Voor alle kinderen die meedoen aan deze studie is een verzekering afgesloten voor onverwachtse schade door deelname aan dit onderzoek. Het betreft de schade door letsel of overlijden die zich openbaart gedurende de deelname aan dit onderzoek en de schade die zich openbaart binnen vier jaar na beëindiging van deelname aan dit onderzoek. De verzekering is voor had Universitair Medisch Centrum Groningen afgesloten bij Onderlinge Waarborgmaatschappij Centramed B.A., Postbus 191, 2270 AD Voorburg.

Vrijwilligheid van deelname

U bent er geheel vrij in wel of geen toestemming te verlenen voor de deelname aan dit onderzoek. Verder heeft u altijd, ook wanneer u schriftelijk heeft verklaard te willen deelnemen, het recht om zonder opgave

van redenen af te zien van verdere deelname aan het onderzoek. Deze beslissing zal geen nadelige gevolgen hebben op de verdere behandeling en geen invloed hebben op de zorg en aandacht, waarop uw kind in ons ziekenhuis recht heeft. Ook uw behandelend arts kan in het belang van uw kind achten het onderzoek voortijdig te beëindigen. Hij/zij zal dit dan met u bespreken.
Indien u er niets voor voelt om met het onderzoek mee te doen zal de tot nu toe gebruikelijke therapie of handelwijze gevolgd worden, de behandelend arts zal op indicatie het bilirubine gehalte bepalen en niet het albuminegehalte.
Mocht in de periode dat u aan het onderzoek deelneemt nieuwe informatie bekend worden die van invloed kan zijn op uw bereidheid om mee te werken, dan zullen wij u hiervan zo spoedig mogelijk op de hoogte stellen, zodat u uw beslissing kunt heroverwegen.

Nadere informatie

Mocht u na het lezen van de brief, voor of tijdens het onderzoek nog nadere informatie willen ontvangen of komen er nog vragen bij u op dan kunt u altijd contact opnemen met de uitvoerders van het onderzoek, Dr. P.H. Dijk en Dr. C.V. Hulzebos telefonisch te bereiken via 050 -3614215. In spoedeisende gevallen kunt u ons ziekenhuis bellen via het algemene telefoonnummer: 050-3616161.
Indien u nadere informatie wenst onafhankelijk van de uitvoerder van het onderzoek dan kunt u contact opnemen met Dr. M. van Stuijvenberg, kinderarts, telefonisch te bereiken op 050-3614215. Dr. van Stuijvenberg is niet direct bij het onderzoek betrokken.

Ondertekening toestemmingsverklaring

Als u besluit mee te werken aan het onderzoek zullen wij u vragen een formulier te ondertekenen. Door ondertekening van dit formulier ('Toestemmingsverklaring' oftewel 'Informed Consent') stemt u in met deelname aan dit onderzoek. U blijft de vrijheid behouden om wegens voor u relevante redenen uw medewerking te stoppen. De arts of verpleegkundige zal het formulier eveneens ondertekenen en bevestigt dat hij/zij u heeft geïnformeerd over het onderzoek, deze informatiebrief heeft overhandigd en bereid is om waar mogelijk in te gaan op nog opkomende vragen.

Brochure

Over deelname aan wetenschappelijk onderzoek in het algemeen is in ons ziekenhuis een brochure beschikbaar. Als u deze niet tegelijk met deze informatiebrief krijgt uitgereikt kunt u deze op verzoek alsnog krijgen.

Dr. P.H. Dijk, kinderarts-neonatoloog
Dr. C.V. Hulzebos, kinderarts-neonatoloog
Tel 050-3614215 via zoemer 55077 of 55078

BIJLAGE BIJ SCHRIFTELIJKE INFORMATIE
ONDERZOEK NAAR HYPERBILIRUBINEMIE BIJ PREMATUREN

-Het reduceren van bilirubine geïnduceerde neurologische schade bij te vroeg geboren kinderen: additioneel gebruik van de bilirubine: albumine ratio voor de behandeling van hyperbilirubinemie -

INFORMATIE INZAKE DE VERZEKERING

Er is een verzekering afgesloten voor onverwachte schade die u lijdt door uw deelname aan dit wetenschappelijk onderzoek. Het betreft de schade door letsel of overlijden die zich openbaart gedurende de deelname aan dit onderzoek en de schade die zich openbaart binnen vier jaar na beëindiging van deelname aan dit onderzoek.

Het bedrag waarvoor de verzekering is afgesloten is maximaal € 5.000.000,- voor de totale schade die zich per verzekeringsjaar bij proefpersonen heeft geopenbaard bij alle onderzoek dat door het Universitair Medisch Centrum Groningen en Rijksuniversiteit Groningen wordt verricht, maximaal € 3.500.000,- voor de totale schade bij dit onderzoek en maximaal € 450.000,- per proefpersoon.

Bepaalde soorten van schade kennen wettelijk gelimiteerde vergoedingen.
Van de dekking door de verzekering is uitgesloten:

- schade van te verwachten risico's zoals beschreven in de schriftelijke informatie voor proefpersonen, tenzij deze ernstiger zijn dan beschreven;
- bij deelname door patiënten: schade door verslechtering van de gezondheid of het uitblijven van de verbetering van de gezondheid;
- schade waarvan (nagenoeg) zeker is dat deze zich bij de proefpersoon zal voordoen;
- schade door aantasting van de gezondheid van de proefpersoon die zich ook geopenbaard zou hebben wanneer de proefpersoon niet aan dit onderzoek had deelgenomen;
- schade die zich bij nakomelingen openbaart als gevolg van een nadelige inwerking van het onderzoek op het genetisch materiaal van de proefpersoon;

De verzekering is afgesloten bij Onderlinge Waarborgmaatschappij Centramed, Postbus 191, 2270 AD VOORBURG onder polisnummer 624.529.102.

Indien u schade heeft geleden of het vermoeden daarvan heeft, dient u zich direct met Dr P.H. Dijk of Dr. C.V. Hulzebos (leden van het onderzoeksteam) telefoonnummer 050-3614215 in verbinding te stellen en de aanwijzingen op te volgen.
Ook kunt u in zo'n geval contact op te nemen met de juridisch stafmedewerker van het Universitair Medisch Centrum Groningen bereikbaar via telefoonnummer 050-3614929 of 050-3614304.

ONDERZOEK NAAR HYPERBILIRUBINEMIE BIJ PREMATUREN

-Het reduceren van bilirubine geïnduceerde neurologische schade bij te vroeg geboren kinderen: additioneel gebruik van de bilirubine: albumine ratio voor de behandeling van hyperbilirubinemie -

TOESTEMMINGSVERKLARING/ INFORMED CONSENT

Wij zijn gevraagd om toestemming te verlenen voor deelname aan bovenvermeld wetenschappelijk onderzoek van:

Naam en voorletters van patiënt/deelnemer: ……………………………………………

Geboortedatum van de patiënt/deelnemer: ……………………………………………..

Wij zijn naar tevredenheid over het onderzoek geïnformeerd. Wij hebben de schriftelijke informatie goed gelezen. Wij zijn in de gelegenheid gesteld om vragen over het onderzoek te stellen. Onze vragen zijn naar tevredenheid beantwoord. Wij hebben goed over deelname aan het onderzoek kunnen nadenken. Wij hebben het recht onze toestemming op ieder moment weer in te trekken zonder dat wij daarvoor een reden behoeven op te geven.

Wij stemmen toe met deelname van bovenvermelde persoon aan het onderzoek.

Wij willen in de toekomst wel benaderd worden voor eventueel vervolgonderzoek.

Naam en voorletters van de ouder 1: ……………………………………………………

Relatie tot patiënt-deelnemer: …………………………………………………………….

Handtekening: ……………………………………………**Datum:**……………………………

Naam en voorletters van ouder 2: ………………………………………………………

Relatie tot patiënt-deelnemer: …………………………………………………………….

Handtekening: …………………………………………… **Datum:** ………………………….

Ondergetekende verklaart dat de hierboven genoemde personen zowel schriftelijk als mondeling over het bovengemelde onderzoek geïnformeerd zijn. Hij/zij verklaart tevens dat een voortijdige beëindiging van de deelname door bovengenoemde persoon van geen enkele invloed zal zijn of de zorg die hem of haar toekomt.

Naam arts/verpleegkundige: ……………………………………………………………

Functie: ……………………………………………………………………………………...

Handtekening: …………………………………………… **Datum:**…………………….........

SYSTEMATIC REVIEW

Reducing bilirubin-induced neurological dysfunction in premature newborns: additional use of bilirubin:albumin ratio in the treatment of hyperbilirubinemia.

This record should be cited as: Dijk PH, Hulzebos CV. A systematic review upon the bilirubin:albumin ratio in the treatment of hyperbilirubinemia in preterm newborns.

Background

Neonatal jaundice due to unconjugated hyperbilirubinemia occurs in almost all preterm infants and is potentially harmful for the central nervous system. Treatment is based on total serum bilirubin (TSB) concentration, but is not evidence based. The risk of bilirubin-induced neurological dysfunction including neurodevelopmental delay (BIND) is in part determined by the total bilirubin pool, but also by the ability of non-albumin bound free bilirubin to enter the brain. Therefore, BIND may depend also on the mutual relation between bilirubin and albumin. The bilirubin:albumin (B:A) ratio is an interesting additional measurement in the management of hyperbilirubinemia.

Objectives

To determine the efficacy of the additional use of the B:A ratio in the management of hyperbilirubinemia, reducing the incidence of BIND including neurodevelopmental delay in preterm infants with unconjugated hyperbilirubinemia when compared to TSB concentrations only.

Search strategy

We searched National library of medicine (Medline): http://www.pubmed.nl, Cochrane Library: http://www.nelh.nhs.uk/cochrane.asp, including Cochrane Database of Systematic Reviews (CDSR), Database of Abstracts on Reviews and Effectiveness (DARE), Cochrane Controlled Trial Register (CCTR), Current Controlled Trials (CCT): http://controlled-trials.com/, ClinicalTrials.gov: http://clinicaltrials.gov/, NHS Centre for Reviews and Dissemination (CRD): http://www.york.ac.uk/inst/crd/crddatabases.htm.
We used the relevant MESH and Text search terms: “hyperbilirubinemia”, “bilirubin”, “jaundice, neonatal” or “kernicterus” in combination with “infant, premature” or “infant, low birth weight” in combination with “serum albumin” or “bilirubin:albumin ratio” or “B:A ratio” in combination with “BIND”, “neurodevelopmental outcome” or “neurotoxicity”. We hand-searched the articles cited in each publication obtained. Two authors extracted data independently.

Selection criteria

We included studies, in which preterm neonates born at gestational age less than 32 weeks (age 28 days of life or less) with unconjugated hyperbilirubinemia due to any cause were analyzed with reference to the association between TSB concentration and and/or B:A ratio, and neurodevelopmental outcome or neurotoxicity. We excluded studies in term and near-term infants.

Results

Initially, we found no references following the search combination "hyperbilirubinemia" and "infant, premature" and "serum albumin" or "B:A ratio" and "neurodevelopmental outcome" or "outcome".

We found and read 21 papers following the search combination "hyperbilirubinemia" or "kernicterus"and "infant, premature" and "serum albumin".

We found 293 references following the search "hyperbilirubinemia" or "kernicterus" and "infant, premature". These abstracts of these references were screened for relevance.

We found 169 references following the search "hyperbilirubinemia" or "kernicterus" and "serum albumin". The abstracts of these references were screened for relevance.

The majority of the abstracts and papers that have been screened did not contain any outcome measure related to neurotoxicity or neurodevelopmental outcome, others did not contain albumin or B:A ratio measurement. All papers with a combination of bilirubin or hyperbilirubinemia, albumin or B:A ratio, prematurity and a outcome related to neurotoxicity (including kernicterus) were included. No additional methodological exclusion criteria were applied due to the small number of relevant papers left.

We found no ongoing clinical trials that evaluate the efficacy of B:A ratio versus TSB in preterm infants with hyperbilirubinemia. We found no systematic reviews or prospective clinical trials evaluating long term neurodevelopmental consequences of using the B:A ratio (with or without the additional use of TSB) versus TSB only in the management of preterm infants with hyperbilirubinemia. One prospective cohort study evaluated short term effects of B:A ratio's in predicting bilirubin encephalopathy (Amin 2001). One studie evaluated retrospecively data of a prospective randomised controlled NICHD phototherapy trial, and studied the association between neurodevelopmental outcome and the B:A ratio (NICHD 1985, Scheidt 1990 and Scheidt 1991). One prospective and two retrospective case-control post-mortum studies analysed the relation between the B:A ratio and kernicterus in preterm infants (Ritter 1982, Kim 1980, Cashore 1982). One patient series reported MRI documented kernicterus and its relation to B:A ratio's (Govaert 2003). Several reviews discussed the use of the B:A ratio in the management of hyperbilirubinemia in preterm infants (Ahlfors 1994, Maisels 2003, Watchko 2003, Bhutani 2004 2x, Kaplan 2005).

Amin et al studied in a single center prospective cohort study the usefulness of the B:A ratio and unbound bilirubin as compared with TSB in predicting bilirubin encephalopathy assessed by serial auditory brainstem responses (ABR) in 143 infants of 28-32 weeks of gestational age (Amin 2001). The mean peak TSB concentration in infants with normal ABR maturation was not significantly different from the mean peak TSB in infants with abnormal maturation.

In the total group there was a trend that B:A ratio was better than TSB in predicting abnormal ABR maturation. In the subset of 45 infants in whom unbound bilirubin was measured, although TSB was not different, there was a significant difference in B:A ratio between the infants with normal versus abnormal ABR maturation. Unbound bilirubin showed to be the most sensitive predictor of abnormal ABR maturation, and hence transient bilirubin encephalopathy in premature newborns with hyperbilirubinemia. The authors support at least to consider the B:A ratio along with the TSB in the management of hyperbilirubinemia in preterm infants.

The National Institute of Child Health and Human Development Cooperative Phototherapy Study was performed in the US between 1974 and 1976 (NICHD 1995, Scheidt 1990 and 1991). This randomised controlled study at six centers comparing the effect of phototherapy versus no-phototherapy for the treatment of hyperbilirubinemia on neurodevelopmental outcome in 1339 infants, included a cohort of low birth weight infants < 2000 gram. It was shown that phototherapy effectively controlled neonatal hyperbilirubinemia without evidence of adverse outcome at 6 years of age.

Scheidt et al analysed a subgroup of 224 preterm / low birth weight infants (< 2000 gram) that were randomised to the control group. The control group did not receive phototherapy and TSB levels were maintained below specified levels by the use of exchange transfusions. In this subgroup the relationship between TSB levels and neurodevelopmental outcome was evaluated. There was no relation between TSB levels and cerebral palsy, and IQ was not associated with TSB levels, time and duration of exposure to bilirubin. The B:A ratio had a near-signficant (p=0.06), though weak, inverse relation with IQ.

Kim et al. reviewed 398 neonatal autopsies in preterm infants (Kim1980). The 27 infants with kernicterus (prevalence of 7%) had relatively low mean TSB peak concentrations, which were similar to those in a restrospective contol group of 103 infants without kernicterus. Serum albumin values and bilirubin binding capacity were significantly lower in kernicteric infants compared to controls. B:A ratios were calculated in six of the 27 kernicteric infants and 15 of the 103 non-kernicteric infants. B:A ratios were similar in both groups.

Cashore et al. determined TSB, free unbound bilirubin, B:A ratio and bilirubin binding affinity in 13 preterm infants < 1500 gram with hyperbilirubinemia before exchange transfusions were performed and later died (Cashore 1982). Five of these 13 infants had kernicterus at autopsy, eight had not. Compared to non-kernicteric infants unbound bilirubin levels were increased and bilirubin binding affinity was decreased in infants with kernicterus, in association with low B:A ratios in the latter.

Ritter et al. prospectively assessed risk factors in the development of kernicterus in 91 infants < 1500 g. (Ritter 1982). In 30 of the 53 infants that died, autopsie was performed. Seven had kernicterus and 23 not. There was no statistically significant difference between infants with and without kernicterus in peak TSB concentration (mean and SEM: 7.3 ±1.3 vs 6.1 ±0. 5 mg/dl), free bilirubin (18.2 ± 4.5 vs 11.1 ± 0.9 nm/l) or albumin (2.8 ±0.2 vs 2.8 ±0.1 g/dl). Although not explicity provided in their paper, B:A ratios also appear to be not statistically different between infants with and without kernicterus (calculated group mean values 2.6 vs 2.2 mg/g).

Govaert et al. reported five preterm infants with clinical signs of kernicterus and related changes in globus pallidus on magnetic resonance imaging (MRI) and/or sonography with TSB levels below currently used exchange transfusion thresholds, but with high B:A ratios (Govaert 2003). In three infants, a combined respiratory and metabolic acidosis had been present around the peak TSB concentration. ABR's were severely impaired in all

preterm infants with elevated B:A ratios and all developed hearing loss facing "accepted" TSB levels. The authors conclude that the pathophysiological role of low serum albumin levels must be considered in BIND especially in acidotic jaundiced preterm infants.
Ahlfors investigated in 54 infants (35 term and 19 preterm) whether the B:A ratio is a reliable predictor of bilirubin-albumin binding by mathematical analyses of the relationship of the B:A ratio to the unbound bilirubin concentration in jaundiced newborns. He found the B:A ratio to be an reliable surrogate for the unbound bilirubin concentration (Alhfors 1994). He concluded that the B:A ratio is a simple nonambiguous way of incorporating the serum albumin concentration into exchange transfusion criteria.
Watchko and Maisels reviewed the pathophysiology, neurodevelopmental outcome and treatment of jaundice in low birth weight infants (Watchko 2003 and Maisels 2003). The authors discussed the lack of legitimate evidence in the treatment of hyperbilirubinemia in preterm infants and summarized the role of albumin binding and B:A ratio. Although no contemporay long term studies relating unbound bilirubin or B:A ratio to developmental outcome are available, the authors support the use of the B:A ratio together with, but not in lieu of, the TSB level as an additional factor in determining the need for exchange transfusion.
Bhutani et al. described six case studies of preterm infants who developed kernicterus (Bhutani 2004). They conclude that the remergence of kernicterus in preterm infants is a matter of concern. They suggest that in the absence of commercial assays for unbound bilirubin or albumin binding reserve, in the mean time, the B:A ratio offers the clinician a reasonable measure of bilirubin binding to albumin in the management of hyperbilirubinemia.
Bhutani and Johnson discussed the need for accurate and precise bilirubin measurements to prevent kernicterus and BIND (Bhutani 2004). The care of the vulnerable sick and preterm infant could be optimized by better techniques to assess the neurotoxic potential of bilirubin. The authors express that measures of bilirubin-binding reserve to albumin and the B:A ratio can be used in the evaluation of preterm infants with hyperbilirubinemia.

Discussion

We found in our review of the literature no evidence from prospective clinical trials that the use of B:A ratios in the management of hyperbilirubinemia reduces long term bilirubin related neurotoxicity. However, it must be recognised that circumstantial evidence illustrates that the additional use of the B:A ratio in the management of hyperbilirubinemia may have beneficial effects in the prevention of BIND in preterm infants.

Recently, several reviews have discussed the role of the bilirubin-albumin binding in the pathophysiology of bilirubin induced neurotoxicity in preterm infants (Ahlfors 2004, Maisels 2003, Kaplan 2005).

Jaundice in newborns result from an increased bilirubin pool due to increased bilirubin production in relation to a relatively low bilirubin elimination. When the bilirubin load exceed the binding capacity of albumin, free bilirubin levels rise. The free bilirubin portion is related to the TSB, and inversely proportional to albumin and its intrinsic ability to bind bilirubine (K). Therefore, the free bilirubin level helps interpret the risk of bilirubin toxicity at a given TSB. Free bilirubin can cross the blood brain barrier and enter the brain. In vitro - and animal studies have shown the

neurotoxic potential of free unconjugated bilirubin. These studies have recently been reviewed by Ostrow 2003 and Tiribelli 2005.
Preterm infants are more prone to bilirubin neurotoxity because of increased bilirubin load, slower elimination and higher susceptibility. Hyperbilirubinemia in preterm infants is more pronounced due to a higher bilirubin production and lower bilirubin exretion, as a result of immaturity of neonatal red cells, hepatic enzymatic conjugation system, hepatobiliary extrection and gastrointestinal sytem. Furthermore, the potential neurotoxic effects of hyperbilirubinemia is increased in preterm infants due to higher accessability of bilirubin to the brain and more pronounced susceptability of the preterm brain to bilirubin. Nevertheless, several neurodevelopmental follow up studies have shown conflicting results regarding the association between peak TSB levels and later adverse outcome in preterm infants (Watchko 2003). Furthermore, preterm infants have lower serum albumin levels. Therefore, bilirubin binding capacity will be decreased and free bilirubin levels increased. Free bilirubin levels are more closely related to short term (ABR maturation abnormalities) and long term outcomes (kernicterus) than TSB (Amin 2001 and Cashore 1980). Nevertheless, there are no studies evaluating the effect of using free bilirubin measurements in the management of hyperbilirubinemia. The lack of these studies is due to the fact that routine clinical labatory measurements of free bilirubin are not generally available. Therefore, the bilirubin:albumin ratio has been used as an surrogate.

Ahlfors (1994) showed that the B:A ratio correlates with measured free bilirubin levels in newborns, indicating that B:A ratio's might be helpfull in predicting neurotoxicity. In retrospective blood sample analyses of preterm infants who died with or without kernicterus, free bilirubin levels and B:A ratio's tend to have a better correlation with kernicterus than the TSB level did (Kim 1980 and Cashore 1982).
Technological advances in research tools such as magnetic resonance imaging (MRI) and auditory brainstem evoked response (ABR) have been used for objective assessments of bilirubin induced neurotoxicity (Shapiro 2003). Govaert showed that typical subtle MRI abnormalties in preterm infants with clinical signs of kernicterus were related to high B:A ratio;s, but not with peak TSB levels (Govaert 2003). The ABR is an objective tool in the evaluation of bilirubin toxicity in term and preterm infants, as has been recently reviewed by Amin 2004 and Shapiro 2005. Amin et al 2001 showed in a prospective cohort study in preterm infants that free bilirubine and the B:A ratio are more closely related than TSB with transient ABR abnormalities. Although this study was not an interventional trial, it suggests that bilirubin is potentially neurotoxic at lower levels in preterm infants, that this effect can be transient, and that B:A ratio's (after free bilirubin) could better than TSB in monitoring preterm infants in the prevention of potential bilirubin toxicity. This study did not assess neurodevelopmental follow-up, yet.
Scheidt et al did study the association between B:A ratio and neurodevelopmental follow up. In their retrospective analyses of the control subgroup of preterm infants who did not receive phototherapy in the NICHD phototherapy study, a near-significant correlation between B:A ratio and neurodevelopmental outcome at 6 years of age was found.
Despite the lack of evidence from randomised controlled trial several experts in the field of bilirubin research and advisory committee's advocate the use of B:A ratio's in term infants with high bilirubin levels close to the exchange

criteria or in the treatment of hyperbilirubinemia in preterm infants (Ahlfors 1994, Maisels 2003, Watchko 2003, Blackmon 2003, Bhutani 2004 2x).

Conclusions

There is no evidence from prospective clinical trials that the use of B:A ratio's in the management of hyperbilirubinemia reduces BIND including neurodevelopmental delay. However, there is circumstantial evidence that the additional use of B:A ratio may be able to prevent BIND in preterm infants. Therefore, along with the opinion of several experts in the field of bilirubin research the reviewers conclude that the additional use of the B:A ratio may have benificial effects in the prevention of BIND in the management of hyperbilirubinemia in preterm infants. Further prospective clinical studies are needed to elucidate this potential effect.

References

Amin et al. Pediatrics 2001;107:664-670. Bilirubin and serial auditory brainstem responses in premature infants.
National Institute of Child Health and Human Development. Pediatrics 1985;75(suppl):385-441. Randmomized controlled trial of phototherapy for neonatal hyperbilirubinemia.
Scheidt et al. Pediatrics 1990;85:455-463. Phototherapy for neonatal hyperbilirubinemia: six year follow up of the national institute of child health and human developmetnt clinical trial.
Scheidt et al. Pediatrics 1991:87:797-805. Intelligence at six years in relation to neonatal bilirubin level: follow up of the national institute of child health and human development clinical trial of phototherapy.
Kim et al. Pediatrics 1980:66:852-858. Lack in predictive indices in kernicterus.
Cashore et al. Pediatrics 1982;69:418-485. Unbound bilirubin and kernicterus in low birth weight infants.
Ritter et al. Pediatrics 1982;69:260-266. A prospective study of free bilirubin and other risk factors in the development of kernicterus in preterm infants.
Govaert et al. Pediatrics 2003;112:1256-1263. Changes in globus pallidus with (pre)term kernicterus.
Ahlfors. Pediatrics 1994;93:488-494. Criteria for exchange transfusion in jaundiced newborns.
Watchko et al. Arch Dis Child Fetal Neonatal Ed 2003:88:F455-F458. Jaundice in low birth weight infants: pathobiology and outcome.
Maisels et al. Arch Dis Child Fetal Neonatal Ed 2003:88:F459-F463. Treatment of jaundice in low birthweight infants.
Bhutani et al. Sem in Perinatol 2004;28:319-325. Kernicterus in sick and preterm infants (1999-2002): a need for an effective preventive approach.
Bhutani et al. Clinical Chemistry 2004;50:477-480. Urgent clinical need for accurate and precise bilirubin measurements in the united states to prevent kernicterus.
Ahlfors et al. Sem in Perinatol. 2004;28:334-339. Bilirubin-albumin binding and neonatal jaundice.
Tiribelli et al. J of Hepatology. 2005;43:156-166. The molecular basis of bilirubin encephalopathy and toxicity.
Kaplan et al. Clin Chem Acta 2005;356:9-21. Understanding severe hyperbilirubinemia and preventing kernicterus.

Shapiro. Pediatr Neurol 2003:29:410-421. Bilirubin toxicity in the developing nervous system.
Amin et al. Sem in Perinatol 2004;28:340-347. Clinical assessment of bilirubin induced neurotoxicity in premature infants.
Shapiro. J of Perinatol. 2005:25:54-59. Definition of the clinical spectrum of kernicterus and bilirubin induced neurological dysfunction.
Blackmon et al. Pediatrics 2004;114:229-233. Research on prevention of bilirubin induced brain injury and kernicterus: NICHD conference executive summary.

RESEARCH PROTOCOL

Reducing Bilirubin-Induced Neurological Dysfunction in Premature Newborns, Additional Use of the Bilirubin:Albumin Ratio in the Treatment of Hyperbilirubinemia

(October 2008)

(TEMPLATE VERSION Oct 2006)

PROTOCOL TITLE: 'Reducing bilirubin-induced neurological dysfunction in premature newborns, additional use of the bilirubin:albumin ratio in the treatment of hyperbilirubinemia'

Protocol ID	ABR 14881 – METc 2006/261
Short title	Reducing BIND in preterm infants
Version - Date	3 - October 2008
Co-ordinating investigator/project leader	Dr. Peter H. Dijk & Dr. Christian V. Hulzebos<br>Beatrix Children's Hospital<br>University Medical Centre Groningen<br>Hanzeplein 1<br>P.O. Box 30.001<br>9700 RB GRONINGEN The Netherlands<br>Tel 050 – 3614215
Project group	Dr. Peter H. Dijk: UMC Groningen<br>Dr. C.V. Hulzebos: UMC Groningen<br>Prof dr A.F. Bos: UMC Groningen<br>Dr. P. Govaert: ErasmusMC Rotterdam<br>Dr. M.J.L.N. Benders: UMC Utrecht<br>Prof dr H.J. Verkade: UMC Groningen<br>Dr. E.M. ter Vergert: MTA UMC Groningen
Principal investigators per site	UMC Groningen: Dr. P.H. Dijk & Dr. C.V. Hulzebos<br>Erasmus Rotterdam: Dr. P. Govaert<br>Amsterdam AMC: Dr. L. van Toledo<br>Amsterdam VU: Prof. dr. W.P.F. Fetter<br>UMG Utrecht: Dr. M. Benders<br>UMC Nijmegen: Dr. K.D. Liem<br>Leiden UMC: Prof. dr. F.J. Walther<br>AZ Maastricht: Prof. dr. J.L.I Zimmermann<br>MMC Veldhoven: Dr. J. Buijs<br>Isala Zwolle: Dr. R.A. van Lingen
Independent physician	Dr. Margriet S. van Stuijvenberg<br>Beatrix Children's Hospital<br>UMC Groningen<br>Hanzeplein 1<br>P.O. Box 30.001<br>9700 RB GRONINGEN<br>tel 050 – 3614215

PROTOCOL SIGNATURE SHEET

Name	Signature	Date
Legal representative<br>Head of Department:	Prof dr A.F. Bos<br>Head of Departement of Neonatology<br>Beatrix Childrens Hospital<br>UMC Groningen<br><br>………………………………	october 2008
Coordinating Investigators	Dr. P.H. Dijk – neonatologist<br><br>………………………………..<br><br>&<br>Dr. C.V. Hulzebos – neonatologist<br><br>…………………………………..	october 2008<br><br>october 2008

TABLE OF CONTENTS

1.	INTRODUCTION AND RATIONALE	10
2.	OBJECTIVES	13
3.	STUDY DESIGN	13
4.	STUDY POPULATION	14
4.1	Population (base)	14
4.2	Inclusion criteria	14
4.3	Exclusion criteria	14
4.4	Sample size calculation	14
5.	TREATMENT OF SUBJECTS	15
5.1	Investigational product/treatment	15
5.2	Use of co-intervention (if applicable)	15
5.3	Escape medication (if applicable)	15
6.	METHODS	16
6.1	Study parameters/endpoints	16
6.1.1	Main study parameter/endpoint	16
6.1.2	Secondary study parameters/endpoints (if applicable)	16
6.1.3	Other study parameters (if applicable)	16
6.2	Randomisation, blinding and treatment allocation	16
6.3	Study procedures	17
6.4	Withdrawal of individual subjects	17
6.4.1	Specific criteria for withdrawal (if applicable)	17
6.5	Replacement of individual subjects after withdrawal	17
6.6	Follow-up of subjects withdrawn from treatment	18
6.7	Premature termination of the study	18
7.	ECONOMIC EVALUATION	19
8.	SAFETY REPORTING	20
8.1	Section 10 WMO event	20
8.2	Adverse and serious adverse events	20
8.2.1	Suspected unexpected serious adverse reactions (SUSAR)	20
8.2.2	Annual safety report	20
8.3	Follow-up of adverse events	20
8.4	Data Safety Monitoring Board (DSMB)	20
9.	STATISTICAL ANALYSIS	21
9.1	Descriptive statistics	21
9.2	Univariate analysis	21
9.3	Multivariate analysis	21

9.4 Interim analysis (not applicable) 21
10. ETHICAL CONSIDERATIONS 22
10.1 Regulation statement 22
10.2 Recruitment and consent 22
10.3 Objection by minors or incapacitated subjects (if applicable) 22
10.4 Benefits and risks assessment, group relatedness 22
10.5 Compensation for injury 23
10.6 Incentives (if applicable) 23
11. ADMINISTRATIVE ASPECTS AND PUBLICATION 24
11.1 Handling and storage of data and documents 24
11.2 Amendments 24
11.3 Annual progress report 24
11.4 End of study report 24
11.5 Public disclosure and publication policy 24
12. REFERENCES 25
SUPPLEMENTS 26
Study guidelines 27
Patient information letter (patienteninformatiebrief) 28
Informed consent (toestemmingsverklaring) 33
Systematic Review 34

LIST OF ABBREVIATIONS AND RELEVANT DEFINITIONS

AAP	American Academy of Pediatrics
ABR	Auditory Brainstem Evoked Responses
ABR	ABR form (General Assessment and Registration form) is the application form that is required for submission to the accredited Ethics Committee (ABR = Algemene Beoordeling en Registratie)
AE	Adverse Event
AR	Adverse Reaction
AUC	Area Under the Curve
B:A ratio	Bilirubin:Albumin ratio
Bf	Free Unbound Bilirubin
BIND	Bilirubin-Induced Neurological Dysfunction
BSID	Bayley Scales of Infant Development
BPD	Bronchopulmonary dysplasia
CA	Competent Authority
CCMO	Centrale Commissie Mensgebonden Onderzoek
CCMO	Central Committee on Research Involving Human Subjects
CRF	Case Record Form
CV	Curriculum Vitae
DOL	Day Of Life
DSMB	Data Safety Monitoring Board
EU	European Union
EudraCT	European drug regulatory affairs Clinical Trials
GA	Gestational Age
GCP	Good Clinincal Practice
IB	Investigator’s Brochure
IC	Informed Consent
IMP	Investigational Medicinal Product
IMPD	Investigational Medicinal Product Dossier
IVH	Intraventricular Hemorrhage
MDI	Mental Developmental Index
METC	Medical research ethics committee (MREC); in Dutch: medisch ethische toetsing commissie (METC)
NEC	Necrotising Enterocolitis
NICHD	National Institute of Child Health and Human Development
NICU	Neonatal Intensive Care Unit
NVK	Nederlandse Vereniging voor Kindergeneeskunde
PDA	Patent Ductus Arteriosus
PDI	Psychomotor Developmental Index

PMA	Postmenstrual Age
PVE	Periventricular Echodensity
PVL	Periventricular Leucomalacia
ROP	Retinopathy of Prematurity
(S)AE	Serious Adverse Event
SD	Standard Deviation
SPC	Summary of Product Characteristics (in Dutch: officiële productinfomatie IB1-tekst)
SUSAR	Suspected Unexpected Serious Adverse Reaction
TSB	Total Serum Bilirubin
Wbp	Personal Data Protection Act (in Dutch: Wet Bescherming Persoonsgevens)
WMO	Medical Research Involving Human Subjects Act (Wet Medisch-wetenschappelijk Onderzoek met mensen

SUMMARY

Background

Neonatal jaundice due to unconjugated hyperbilirubinemia occurs in almost all preterm infants and is potentially neurotoxic. Treatment is based on total serum bilirubin (TSB), but is not evidence based. TSB is an unreliable predictor of bilirubin induced neurological dysfunction (BIND). Low albumin levels increase free bilirubin (Bf) levels and may potentiate BIND. The bilirubin:albumin ratio is an interesting additional method in the management of hyperbilirubinemia.

Objective

The research question of this study is whether BIND is reduced using B:A ratio together with TSB versus TSB-only as indicator for treatment of hyperbilirubinemia in preterm infants.

Study design

Prospective, randomized, controlled, open label, blinded outcome cost-effectiveness multicenter study in tertiary neonatal intensive care units in the Netherlands.

Study population

Preterm infants born at a gestational age of less than 32 weeks postmentrual age.

Intervention

Hyperbilirubinemia is evaluated daily using the B:A ratio together with TSB (study group) *versus* TSB only (control group – care as usual). Treatment guidelines are based on B:A ratio and TSB (whichever comes first) in the study group *versus* on TSB-only in control group.

Main study parameters/endpoints

The primary outcome variables are the neurodevelopmental index scores at 18-24 months of age, using a standardised neurological examination and composite motor score and composite cognitive score (Bayley Scale of Infant Development-III). Secondary outcome variables are: peak TSB, peak B:A ratio, duration of hyperbilirubinemia, number and duration of phototherapy, number of exchange transfusion, and all the “complications of prematurity” such as mortality, respiratory distress, bronchopulmonary dysplasia (BPD), retinopathy of prematurity (ROP), patent ductus arteriosus (PDA), necrotising enterocolitis (NEC), intraventricular hemorrhage (IVH), periventricular echodensitity (PVE) and periventricular leucomalacia (PVL).

Economic evaluations are part of this study. Costs-effectiveness assessments will be performed from a hospital perspective with a time-horizon of 18-24 months. Costs of diagnostics and treatments will be included. Long-term effects of neurological impairment and developmental delay on costs are estimated using modelling techniques.

Nature and extent of the burden and risks associated with participation, benefit and group relatedness:
Daily laboratory examinations will include bilirubin and albumin levels for the first ten days of life in both study groups . Usually, these measurements do not require extra blood volume. Sometimes, more blood is required (maximal 10 x 0.1 ml = 1.0 ml), but no extra puncture will be necessary. It is possible that more children will be treated with phototherapy and/or exchange transfusion in the study group in comparison to the care-as-usual protocols On the other hand, an earlier start phototherapy may reduce TSB-levels and prevent exchange transfusions. The net effect cannot be predicted.
A part of the study-population (i.e. infants admitted to the NICU of the UMC Groningen) will be subjected to a more extended audiological evaluation (ABR) during the first 10 days of life , than in the routine protocol for NICU patients (Algo-screening). In analogy of the Algo-screening, the ABR consists of auditory stimuli by headphone, registered by adhesive head-electrodes (3 instead of 1). Full developmental tests are included of this study protocol. These tests are part of the standard follow-up visits of NICU-graduates (care as usual).

Time schedule
Anticipated start of this study is March 2007. Inclusion period of 9 months. Follow-up at 18-24 months of age. Total duration of study is 36 months. End of study is March 2010.

Financial support
This study is financed by grant nr 945-07-407 of the “Doelmatigheidsonderzoek” program DO 07 of the ZonMw.

1. INTRODUCTION AND RATIONALE

Problem definition

Unconjugated hyperbilirubinemia occurs in nearly all preterm infants (Watchko 1992). Free bilirubin, the fraction not bound to albumin, is toxic for the central nervous system (Shapiro 2003). Free bilirubin is difficult to measure and routine clinical laboratory measurement is not available. Therefore, the TSB level is used to evaluate hyperbilirubinemia, but is not a good predictor of neurotoxicity. Follow-up studies that have evaluated the association between TSB and adverse outcome are inconclusive, and most failed (Van de Bor 1989 and 1992, Watchko 2003, Oh 2003). Preterm newborns are at greater risk for BIND than term newborns, but precise TSB levels at which treatment should be initiated to prevent neurological damage are not known. In addition, preterm newborns do not exhibit acute neurologic clinical signs of bilirubin encephalopathy seen in term newborns (Amin 2001,Govaert 2003). It is difficult to determine whether a preterm infant is at risk for bilirubin neurotoxicity, and no evidence-based management guidelines for preterm infants with hyperbilirubinemia exist. The aim of treatment is to prevent BIND while not causing harm. Phototherapy and exchange transfusions are effective and relatively safe in term newborns. Preterm newborns are much more likely to experience complications of exchange transfusions, such as arrhythmia, thrombosis, thrombocytopenia, necrotising enterocolitis, infections or death (Maisels 2003). Phototherapy is effective in the prevention for the need of exchange transfusions and side-effects are considered rare (Maisels 2003).

In summary, it is a healthcare problem that despite effective treatment strategies for hyperbilirubinemia, BIND still occurs in preterm infants.

Disease subject in this study

Hyperbilirubinemia and BIND

Subgroup of patients

Preterm infants < 32 weeks of gestational age.

Variation in sex, age and cultural background: Bilirubin production, metabolism and neurotoxicity may be influenced by gestational age, sex, race, feeding and possible risk factors for bilirubin neurotoxicity such as asphyxia, acidosis, sepsis, hemolysis and intracranial bleeding. Subgroup and multivariate analyses will be performed.

Usual care in the Netherlands

The American Academy of Pediatrics (AAP 2004) has recently published guidelines for the management of hyperbilirubinemia in term newborns based on TSB levels. This guideline will be adapted for the Netherlands supported by the Nederlandse Vereniging voor Kindergeneeskunde (NVK). For preterm infants no general accepted guidelines for the management of hyperbilirubinemia are available. Therefore, the usual care is not uniform. Also in the Netherlands several treatment schemes are in use, often adopted from text-books in neonatology. The schemes use gestational age (or birthweight), postnatal age and TSB, and reflect TSB thresholds at which phototherapy is initiated and higher levels at which exchange transfusion is recommended.

Who are involved in the usual care

Preterm infants born at GA less than 32 weeks are treated in 10 neonatal intensive care units in the Netherlands. Neonatologists and pediatricians are responsible for the medical care.

Motivation of the chosen intervention

Bilirubin neurotoxicity is mainly determined by free bilirubin, which is very difficult to measure. The B:A ratio is an estimate of free bilirubin and might therefore be a better indicator for a harmful bilirubin level than TSB. High levels of free bilirubin have been associated with BIND in preterm infants (Cashore 1982, Nakamura 1992). The B:A ratio correlates with free bilirubin and, has been used in determining the need for exchange transfusion (Ahlfors 1994). In addition, raised free bilirubin concentrations are more closely associated than TSB levels with signs of acute neurotoxicity in auditory brainstem evoked responses (ABR) (Amin 2001). There are no contemporary long-term studies in preterm infants relating free bilirubin levels and acute neurotoxic signs to long-term neurodevelopmental outcome.

We propose to investigate the use of B:A ratio along with TSB vs TSB only as markers of bilirubin toxicity and as intervention markers. The intervention thresholds are based on those published by Maisels 2003 and Ahlfors 1994. The B:A ratios are derived from TSB (umol/L) divided by 25 g/L for infants < 1250g and divided by 30 g/L for infants > 1250 g, which are albumin levels considered to be safe with regard to bilirubin-binding. Lower albumin levels result in higher B:A ratios that correspond with increased free bilirubin levels. The B:A thresholds are also based on the recommendations of Maisels 2003 and Ahlfors 2003.

We chose to investigate the effect on neurodevelopmental outcome at 18-24 months of age because it is one of the most relevant outcomes in neonatology (Shapiro 2005). We intend additional neurodevelopmental follow-up assessments at later ages, but those are beyond the scope of this study protocol.

Relevance

This study may contribute to the resolution of the healthcare problem in several ways. First, this study will elucidate whether the B:A ratio is an additional marker to TSB in predicting acute and chronic neurotoxicity causing neurodevelopmental impairment in preterm infants. Therefore neurotoxicity can be prevented, and the neurodevelopmental outcome of vulnerable preterm infants might improve.

Second, this multicenter study can provide evidence on the proposed management guidelines and uniform the wide range of currently used guidelines in the management of hyperbilirubinemia in preterm infants. Several foreign experts in the field state that our study could provide novel information on this topic.

Other studies related to this healthcare problem

In the USA a NICHD Neonatal Research Network randomised control study in preterm infants comparing conservative versus prophylactic i.e. aggressive use of phototherapy and exchange transfusion is underway. The main healthcare problem of bilirubin neurotoxicity in preterm infants is the same, but research questions and the chosen intervention are different from our study. The recently published results of the Aggressive vs. Conservative phototherapy trial (Morris BH et al., N Engl J Med. 2008 Oct 30;359(18):1885-96), prompt us, for comparative reasons, to perform subgroup analyses based on birth weight groups. Subgroup analyses will be conducted for two birthweight groups: ≤ 1000 g versus > 1000 g.

Reports by advisory boards on this subject

The American Academy of Pediatrics (AAP 2004) and the National Institute of Child Health and Human Development (Blackmon 2004) have called for further research into the clinical use of bilirubin – albumin relation to improve the ability to determine those babies truly needing treatment. The NICHD called for development of rigorous validation of measures to detect and quantify early manifestations of bilirubin-induced brain injury and to

confirm the time-course of such manifestations in response to interventions including: (amongst others) neurophysiologic testing, including ABR monitoring (AAP 2004). The current American Academy of Pediatrics (AAP 2004) guidelines for managing healthy jaundiced term and near term newborns recommends the use of the bilirubin/albumin ratio in addition to the total serum bilirubin.
This study is supported by the Kwaliteitsbureau of the Nederlandse Vereniging voor Kindergeneeskunde.

Incidence of the targeted (sub)population
In the Netherlands, each year about 1400 infants are born with a gestational age of less than 32 weeks. Almost all these infants are treated in the ten NICUs in the Netherlands. Visible jaundice is an almost universal feature in these preterm children. Therefore, the majority is treated with phototherapy for several days. Only a small number preterm infants (estimated at about 20-50 per year in NL) is treated with exchange transfusions for more severe hyperbilirubinemia.

Estimated potential effects on health from the interventions evaluated in this study
At 18-24 months of age 40% of the children who were born before 32 wks of GA have neurodevelopmental delay (Stoelhorst 2003). This delay is due to several variables during infancy, and bilirubin toxicity is probably one of them. The potential effects of using the B:A ratio in addition to the TSB-level versus the TSB only (usual care) as diagnostic determinant for starting (and stopping) treatment (phototherapy or exchange therapy) for hyperbilirubinemia are based on the assumption that the B:A ratio is more closely related to the potential toxic free bilirubin levels than the TSB is. In preterm infants with low albumin levels, who are more vulnerable to bilirubin toxicity, therapy will be started at relatively (related to albumin) low bilirubin levels. Therefore, therapy will be started in those infants that really need it .We expect that in the vulnerable group of infants with low levels of albumin, bilirubin neurotoxicity and consequently neurodevelopmental delay will be reduced.

Estimated potential effects on costs from the interventions evaluated in this study
We expect that with a more selective application of the treatment of hyperbilirubinemia we are able to reduce the number of infants with neurological damage. The ex-preterm infants with neurological damage need special treatment e.g. physiotherapy, speech-language-communication-training, hearing-aids, special education and medical check-ups. In the group infants who are more vulnerable to bilirubin toxicity, with relatively low levels of albumin, the number of phototherapy days will probably increase but it is anticipated that the neurological outcome will be better in comparison to the usual care. Although short-term costs might increase (albumin measurements and phototherapy), life-time costs as a result of neurological disabilities will be reduced.

2. OBJECTIVES

The primary objective of this study is to evaluate whether the combination of B:A ratio together with TSB is a better marker for a harmful unconjugated bilirubin level than TSB only. Using this combination of B:A ratio and TSB in the treatment of hyperbilirubinemia will lead to better treatment and reduce BIND.
The primary research question is therefore: Is BIND reduced by using the combination of B:A ratio and TSB *versus* TSB only as indicators for phototherapy and exchange transfusion for the treatment of hyperbilirubinemia in preterm infants (born at less than 32 weeks PMA) ?

3. STUDY DESIGN

Prospective randomized controlled open label, blinded endpoints multicenter study in the ten Neonatal Intensive Care Units in the Netherlands (see flowchart).

Flowchart

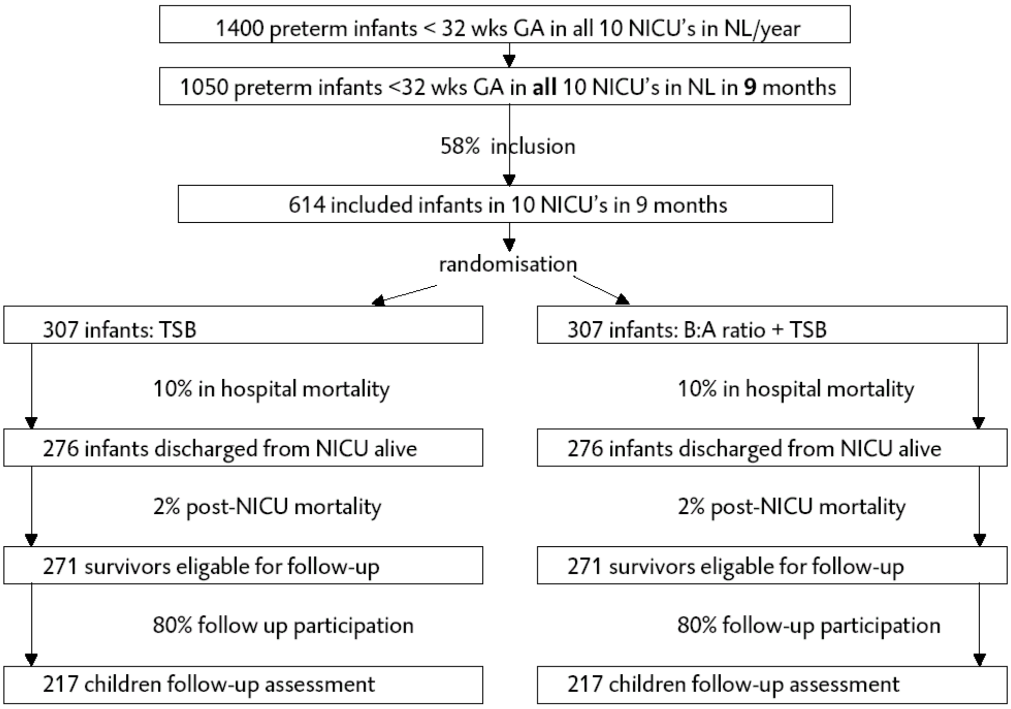

Poweranalysis: for a difference of 7 points in MDI/PDI (mean 96 +/- SD 26)
alpha 0.05 and beta 0.20, 2 x 217 subjects for assessment

4. STUDY POPULATION

4.1 Population (base)

All preterm infants born at less than 32 weeks of PMA treated in one of the ten NICU's in the Netherlands are eligible for this study.

In the Netherlands all infants born before 32 weeks of gestational age are treated in one of the ten NICU's. Non-NICU hospitals have an obligation to send mothers with imminent preterm delivery before 32 weeks of GA and the preterm infants born before 32 weeks PMA to the regional NICU. In the Netherlands each year about 1400 infants are born with a GA of less than 32 weeks. The majority of these infants are inborn (intrauterine transport of the infant). A minority of the children born before a gestational age of 32 weeks are transported postnatally, in their first day of life.

4.2 Inclusion criteria

- Prematurity (< 32+0 weeks of PMA)
- Admitted to a NICU in less than 24 hours after birth
- Informed consent of the parent or legal guardian

4.4 Exclusion criteria

Major congenital malformations, clinical syndromes and chromosomal abnormalities

4.4 Sample size calculation

Neurodevelopmental outcome at 18-24 months of age using mental – and psychomotor development scores (MDI and PDI) are assessed using the Dutch version of Bayley scales of infant development. A significant association has been reported between peak TSB concentration and PDI in a retrospective cohort analysis of the NICHD (Oh 2003). The PDI scale has a population mean of 100 and a standard deviation of 16, but a reference (i.e. in preterm infants <32 weeks) mean of 96 (SD 26) (Oh 2003, Stoelhorst 2003). Both scales range from 51 to 149. An MDI or PDI score of more than 85 is considered normal, a score between 68 and 84 is considered moderate delay and scores less than 68 as severe delay. A difference of 5 - 10 points in developmental score is considered relevant (personal communication Dijk/Hulzebos with all neonatologists in the Netherlands during a consensus meeting (Neoned Retraite in January 2006). With an alpha of 0.05 and power of 80% a change of 7 points on a 100 points scale, with a standard deviation of 26, 2x 217 subjects need to be assessed. If 10% mortality and 20% drop outs are considered in this population, a total of 2 x 307 subjects need to be included (INSTAT statistics, see flow chart). In a consensus meeting with all participating NICUs and representatives of the Netherlands Neonatal Follow-Up organisation (May 2008) it was agreed to replace the 2nd version of the Bayley Scales of Infant Development (BSID II) by the 3rd version (BSID III). After that meeting, the BSID III is used for routine clinical follow-up in all preterm born infants with a gestational age of less than 30 weeks, and for all children included in this clinical trial. All agreed to test the composite motor score scales (primary endpoint) and composite cognitive score scales.

5. TREATMENT OF SUBJECTS

5.1 Investigational treatment

This study compares two methods of management of hyperbilirubinemia in preterm infants. In the control group, the management of hyperbilirubinemia is based on TSB levels only (care as usual). The thresholds of treatment of hyperbilirubinemia with phototherapy or exchange transfusion are based on TSB only. In the study group a combination of TSB and B:A ratio is used. The thresholds of treatment (phototherapy and exchange transfusion) are based on both, TSB and B:A ratio, whichever comes first. Whenever one of the two markers of hyperbilirubinemia (TSB or B:A ratio) reaches the predefined threshold values, treatment should be started.

5.2 Use of co-intervention

The use of albumine infusion in the treatment of hyperbilirubinemia is discouraged in this study. Treatment with albumine infusion for other indications is not restricted. The study protocol has no restrictions with regard to co-interventions, co-medication or other specific guidelines. The study subjects are treated according to the guidelines of the individual NICU's.

5.3 Escape medication / treatment

Not applicable

6. METHODS

6.1 Study parameters/endpoints

6.1.1 Main study parameters/endpoints

The primary endpoint of this study is the neurodevelopmental outcome at the age of 18-24 months using standardised neurological examination and developmental scores: composite motor score and composite cognitive score are assessed using the Dutch version of the BSID III.

6.1.2 Secondary study parameters/endpoints

Secondary endpoints are bilirubin-related parameters with amongst others peak bilirubin levels, duration of hyperbilirubinemia, peak B:A ratio, number and duration of phototherapy, number of exchange transfusions.
Other secondary outcome parameters are those related to “the complications of prematurity” which are potential confounders for neurodevelopmental outcome. The complications of prematurity are mortality, respiratory distress syndrome (RDS), bronchopulmonary dysplasia (BPD), patent ductus arteriosus (PDA), necrotizing enterocolitis (NEC), retinopathy of prematurity (ROP), infections, intraventricular hemorrhage (IVH), periventricular echodensities (PVE) and periventricular leucomalacia (PVL).

6.1.3 Other study parameters/endpoints

Other study are potential intermediate variables in the evaluation of bilirubin metabolism and -toxicity. These parameters are free bilirubin levels, lumirubine and ABR-measurements.

Other study parameters are those involved in the economic evaluation or cost-effectiveness analyses and contain the direct and indirect costs of management of hyperbilirubinemia, prematurity and developmental delay.

It is the intention to perform additional neurodevelopment assessments at later ages (4-7years), but those are beyond the scope of this study protocol.

6.2 Randomisation, blinding and treatment allocation

Assessment for eligibility is performed by neonatologists and/residents of the participating NICU. They initially ask whether the parents will consider participation of their child and provide written patient information. The co-ordinating research nurses/investigators inform the parents in detail. A 24 hrs – 7 days schedule will be made up to be able to inform and enrol participants day and night, 7 days a week. The written information contains information on the objectives, design, methods, duration, possible advantages and disadvantages of the study treatments, and information that non-co-operation with the study or withdrawal will not have consequences for the treatment of their child. Research nurses/investigators obtain written informed consent and are available for additional information to the parents. On demand, parents can obtain additional information from an independent pediatrician. Parents are allowed to consider participation for several hours, with a maximum of 24 hours, since subjects need to bee included during their first day of life.
After informed consent has been obtained study subjects are randomised using a web-based computer scheme. The subjects are assigned in a 1:1 ratio to one of the two study groups. Randomisation is stratified according to the study site (NICU) and gestational age group (two groups: 24^{+0} - 28^{+6} weeks and 29^{+0} - 31^{+6}weeks) .
Blinded treatment assignment is not possible. Clinicians should be aware whether to apply the hyperbilirubinemia guidelines based on TSB (control group) or B:A ratio and/or TSB (study group). Blinding of the study subjects is not

an issue in preterm infants. The parents are not aware of group-allocation to secure blind assessment of the primary endpoint . The main study endpoints are assessed blindly. Neurodevelopmental outcome tests are performed by neonatologists and assistants who are unaware of the group allocation of the subjects.

6.3 Study procedures

In both study groups hyperbilirubinemia is evaluated daily for the first ten days of life. Treatment of hyperbilirubinemia will be based on total serum bilirubin in the care as usual group, whereas in the study group the combination of bilirubin:albumin ratio and total serum bilirubin will guide treatment. In both groups total serum bilirubin and albumin will be measured for post-hoc analyses purposes.
In general no extra blood volume is needed for these measurements, because these measurements are part of the daily routine blood examinations. In some cases this may cost several tenths of millilitres blood extra (maximal 10x0.1ml=1.0ml), but no extra punctures will be necessary.
The primary outcome is neurodevelopmental outcome by 18-24 months of age and consists of a standardised neurological physical examination and assessments of developmental scores: motor and cognitive scores using the Dutch version of the Bayley scales of Infant Development (BSID-III-NL). The composite cognitive score assesses environmental responsiveness and sensory and perceptual abilities, memory, learning abilities. The composite motor score assesses both gross and fine motor skills.
Mental, motor and behaviour functions are tested by playing and observations. The test duration is about 60 min. Each child will undergo a standardised pediatric neurologic evaluation to assess the quality of their motor skills, co-ordination, gait and behaviour. Cerebral palsy is diagnosed with the use of standard criteria, including the location or body part impaired (e.g. hemiplegia or diplegia), the degree of impairment of muscle tone, and reflexes, and the effect of the condition on ambulation. Audiologic and visual impairment are tested by standardised tests.
Residual blood samples will be stored for free bilirubin measurements: no extra punctures will be done for this purpose.
Residual urine samples that are collected as part of the routine clinical treatment, will be stored for lumirubine measurements.
In a subgroup of children (i.e. infants admitted to the NICU of the UMC Groningen), serial automated brainstem evoked potentials (ABR) will be assessed using a earphone to apply sound-clicks and self-adhesive electrodes applied to the head to record the time that sound-stimuli need to reach the brain.

6.5 Withdrawal of individual subjects

Subjects can leave the study at any time for any reason if they (their parents/caretakers) wish to do so without any consequences. The investigator can decide to withdraw the subject from the study for urgent medical reasons.

6.4.1 Specific criteria for withdrawal

Specific criteria for withdrawal are those circumstances in which the exclusion criteria (major congenital malformations, clinical syndromes and chromosomal abnormalities) are being diagnosed after inclusion.
Death and other serious complications of prematurity are not a reason to withdraw subjects.

6.5 Replacement of individual subjects after withdrawal

The number of withdrawn subjects will be replaced.

6.7 Follow-up of subjects withdrawn from treatment

Subjects withdrawn from the study will be treated according to the usual care including neurodevelopmental outcome assessment which is part of the usual follow up of NICU-graduates.

6.7 Premature termination of the study

The primary outcome variable of this study is neurodevelopmental outcome at 18-24 months of age, which is beyond the timespan of the inclusion period of this study (9 months). Therefore, interim analyses or application of stopping rules during the inclusion period is not relevant. A Data Safety Monitoring Board will monitor the study population on safety aspects. This Board may advise the Project group to alter the study protocol or even terminate the study in case of (unexpected) serious adverse events. Please see chapter 8: “Safety Reporting”.

7. Economic evaluation (MTA)

This study includes an economic evaluation, and will be conducted as a cost-effectiveness analysis. Therefore, the costs of care for preterm infants that are evaluated using TSB versus B:A ratio along with TSB, will be compared by looking at the incremental costs per additional point on the PDI scale at 18-24 months. A more favourable score on the PDI scale is expected in the group treated according to the B:A ratio. The evaluation will be conducted from a hospital perspective including direct medical costs. Costs of diagnostics (bilirubin and albumin measurements) as well as treatment in case of unconjugated hyperbilirubinemia (phototherapy and exchange transfusions) will be taken into account. A case record form (CRF) will be used to collect costs for each patient during the study period. In addition, a decision-analytic model will be constructed to assess the long-term cost-effectiveness of prevention of bilirubin induced neurological damage. Costs of long-term effects of neurological impairments such as hospital stay, doctors-visits, hearing-aids, physiotherapy, and speech-therapy will be based on both retrospective data from our institutions and data from the literature (e.g. Io 2004, Hack 2000). The model will be built with Tree-Age Pro Healthcare Edition. In addition, uncertainty surrounding the incremental cost-effectiveness will be presented using a cost-effectiveness plane. Cost components will be vaulted according to the Dutch guidelines for economic evaluation (CVZ).

Time horizon of this study is 18-24 months for the prospective economic evaluation and lifetime for the analysis with the model. Due to a long time-horizon, discounting will be applied. A sensitivity analysis will be conducted to estimate the impact of variation of major cost elements as well as different discount rates.

9. SAFETY REPORTING

8.1 section 10 WMO event

In accordance to section 10, subsection 1, of the WMO, the investigator will inform the subjects (parents/caretakers) and the reviewing accredited METC if anything occurs, on the basis of which it appears that the disadvantages of participation may be significantly greater than was foreseen in the research protocol. The study will be suspended pending further review by the accredited METC, except insofar as suspension would jeopardise the subjects' health. The investigator will take care that all subjects are kept informed.

8.2 Adverse and serious events

This study is evaluating long-term consequences of diagnostic procedures on neurodevelopmental outcome and is not an investigational medical product study. Treatment modalities in this study protocol are equal to those applied in the usual care. It may be possible that more children will be treated with phototherapy and/or exchange transfusion in the study group in comparison to the care-as-usual protocols. Phototherapy and exchange transfusions are effective and relatively safe in term newborns. Preterm newborns are much more likely to experience complications of exchange transfusions, such as arrhythmia, thrombosis, thrombocytopenia, necrotising enterocolitis, infections or death (Maisels 2003). Phototherapy is effective in the prevention for the need of exchange transfusions and side-effects are considered rare (Maisels 2003). Therefore, earlier phototherapy may reduce TSB-levels and prevent exchange transfusions. The net effect cannot be predicted.

Other adverse and serious events are not to be expected and not related to the study protocol. Preterm infants treated in NICUs often suffer from serious effects. These complications of prematurity are recorded in the CRF and analysed continuously and monitored by the Data Safety Monitoring Board.

8.3 Follow-up of adverse events

All infants will participate in the usual NICU-graduate follow-up. This follow-up program is targeted at evaluating and coordinating diagnostic procedures and treatment of all prematurity related problems, in close cooperation with regional and local pediatricians.

8.4 Data Safety Monitoring Board

During the inclusion period of 9 months until 3 months thereafter the Data Safety Monitoring Board will analyse the incidence of the above mentioned complications of prematurity in both study groups. The Board will, as needed, review individual records to be able to analyse potential associations between complications and the study protocol. The Board may advise the Project Group to alter the study protocol or even terminate the study in case of serious adverse effect. The personal composition of the Data Safety Monitoring Board has to be determined.

9. STATISTICAL ANALYSES

9.1 Descriptive statistics

All randomised subjects will be analysed on the intention to treat basis. The unpaired two-sided Student's t-test will be used to compare the primary outcome data (i.e. composite motor score) in the study group versus the control group. Categorical data will be compared using two-sided Chi-square tests with continuity correction or Fischer's exact test. Normally distributed continuous variables are compared using the unpaired Student's t-test. Nonparametric continuous variables are compared using the Wilcoxon rank sum test or Mann-Whitney U-test. Significance is defined as $p<0.05$. All variables are tested two-sided.

9.2 and 9.3 Univariate analysis & multivariate analysis

In post- hoc analyses logistic regression models will be applied to analyse the association between bilirubin variables (peak TSB, peak B:A ratio, duration of hyperbilirubinemia etc) and neurodevelopmental outcome variables. Demographic risk factors (e.g. gestational age, birthweight, sexe, mothers' educational level) and other confounders (complications of prematurity such as: intracranial hemorrhage, periventricular leucomalacia, postnatal steroids, necrotising enterocolitis, bronchopulmonary dysplasia, retinopathy of prematurity, late sepsis) will be included in multivariable logistic regression models.

The recently presented results of the NICHD aggressive vs. conservative PT trial (Morris BH et al., N Engl J Med. 2008 Oct 30;359(18):1885-96) prompt us, for comparative reasons, to perform subgroup analyses based on birth weight groups in stead of subgroup analyses based on gestational age groups. Therefore, subgroup analyses will be conducted for two birth-weight groups: ≤ 1000 g versus > 1000 g.

9.4 Interim analysis

Interim analyses are not performed. This study is an long-term outcome study. The outcome assessment at the age of 18-24 months is beyond the end of the inclusion period (9 months). Therefore, it is not relevant to perform interim analyses on the primary outcome.

10. ETHICAL CONSIDERATIONS

10.1 Regulation statement

This study will be conducted according to the principles of the Declaration of Helsinki (version 9.10.2004) and in accordance with the Medical Research Involving Human Subjects Act (WMO).

10.2 Recruitment and consent

Assessment for eligibility is performed by neonatologists and/residents of the participating NICU's. They initially ask whether the parents would consider participation of their child and provide written patient information. The co-ordinating research nurses/investigators inform the parents in detail. A 24 hrs – 7 days schedule will be made up to be able to inform and enrol participants day and night, 7 days a week.. The written information contains information on the objectives, design, methods, duration, possible advantages and disadvantages of the study treatments, and information that non-co-operation with the study or withdrawal will not have consequences for the treatment of their child. Research nurses/investigators obtain written informed consent and are available for additional information to the parents. On demand, parents can obtain additional information from an independent pediatrician. Parents are allowed to consider participation for several hours, with a maximum of 24 hours, since subjects need to bee included during their first day of life. (please see patient information letter and informed consent letter)

10.3 Objection by minors or incapacitated subjects.

This study will be conducted according to the principles of the code of conduct for resistance in minors participating in medical scientific research (Gedragscode bij verzet van minderjarigen die deelnemen aan medisch-wetenschappelijk onderzoek), stated by the Dutch society of pediatrics: Nieuwsbrief Nederlandse Vereniging voor Kindergeneeskunde (NVK), nummer 3, 2001.

10. 4 Benefits and risks assessments, group relatedness

Neonatal jaundice due to unconjugated hyperbilirubinemia is a physiological phenomena of the newborn infant. Hyperbilirubinemia is potentially neurotoxic and related to psychomotor developmental impairment. Studies comparing different management strategies of hyperbilirubinemia in preterm infants assessing neurodevelopmental outcome can only be conducted in preterm infants. There are no other, less vulnerable, potential study subjects in which our research question can be studied. The main goal of this study is to reduce neurodevelopmental impairment in preterm infants. In the Netherlands about 40% of the ex-preterm NICU-graduates have delayed mental and psychomotor development. Therefore, individual subjects of the study population could benefit from this study, by a better neurodevelopmental outcome, if our hypothesis appears to be true.
To our opinion, the risks and hazard associated with participation to this study can be considered negligible and burden minimal. In the study group the B:A ratio is used as marker for potential neurotoxic hyperbilirubinemia, in addition to the total serum bilirubin (TSB). In the control group, in which the care as usual is applied, only the TSB level is taken into account. Therefore this study can be considered safe.

10.5 Compensation for injury

The sponsor/investigator has a liability insurance which is in accordance with article 7, subsection 6 of the WMO.

The sponsor (also) has an insurance which is in accordance with the legal requirements in the Netherlands (Article 7 WMO and the Measure regarding Compulsory Insurance for Clinical Research in Humans of 23rd June 2003). This insurance provides cover for damage to research subjects through injury or death caused by the study.

4. € 450.000,-- (i.e. four hundred and fifty thousand Euro) for death or injury for each subject who participates in the Research;
5. € 3.500.000,-- (i.e. three million five hundred thousand Euro) for death or injury for all subjects who participate in the Research;
6. € 5.000.000,-- (i.e. five million Euro) for the total damage incurred by the organisation for all damage disclosed by scientific research for the Sponsor as 'verrichter' in the meaning of said Act in each year of insurance coverage.

The insurance applies to the damage that becomes apparent during the study or within 4 years after the end of the study.

10.6 Incentives

Eligible participants do not receive any special incentives that may encourage participation in this study.

11. ADMINISTRATIVE ASPECTS AND PUBLICATION

11.1 Handling and storage of data and documents

Data are collected in digital patient record forms. Study data are handled confidentially and anonymously and in accordance with the Dutch personal data protection act (de wet bescherming persoonsgegevens). Subjects have unique identification codes (study number), that are not logically related to the personal data. The list of codes that link the study numbers to the individual subjects is safeguarded by the central investigator. Blood samples and study related data that are not directly linked to clinical patient management are stored using that study number.

11.2 Amendments

All substantial amendments will be notified to the METC of the UMC Groningen. Non-substantial amendments will not be notified to the accredited METC and the competent authority, but will be recorded and filed by the investigator.

11.3 Annual progress reports

The investigator will submit a summary of the progress of the study to the accredited METC once a year.
Information will be provided on the date of inclusion of the first subject, numbers of subjects included and numbers of subjects that have completed the trial, serious adverse events/ serious adverse reactions, other problems, and amendments.

11.4 End of study report

The investigator will notify the accredited METC of the end of the study within a period of 8 weeks. The end of the study is defined as the last patient's last visit. In case the study is ended prematurely, the investigator will notify the accredited METC, including the reasons for the premature termination.
Within one year after the end of the study, the investigator will submit a final study report with the results of the study, including any publications/abstracts of the study, to the accredited METC.

11.5 Public disclosure and publication policy

This clinical trial will be registered in a clinical trial register (Nederlands Trial Register at www.trialregister.nl). Papers for publication in scientific literature will be admitted on behalf of the "project group on hyperbilirubinemia in preterm infants". Members are the members of the scientific project group, one co-ordinating investigator of each participating centre, and MTA investigators.

13. REFERENCES

Watchko JF, Oski FA. Kernicterus in preterm newborns: past, present, and future. Pediatrics 1992; 90: 707-715.

Shapiro SM. Bilirubin toxicity in the developing nervous system. Pediatr Neurol 2003; 29: 410-421.

Van de Bor et al. Hyperbilirubinemia in preterm infants and neurodevelopmental outcome at 2 years of age. Pediatrics 1989;83:915-920.

Van de Bor et al. Hyperbilirubinemia in low birth weight infants and outcome at 5 years of age. Pediatrics 1992;89:359-364.

Watchko JF, Maisels MJ. Jaundice in low birth weight infants: pathobiology and outcome. Arch Dis Child Fetal Neonatal Ed 2003;88:F455-F458.

Oh W, Tyson JE, Fanaroff AA et al. Association between peak serum bilirubin and neurodevelopmental outcomes in extremely low birht weight infants. Pediatrics 2003;112:773-779.

Govaert P et al. Changes in globus pallidus with preterm kernicterus. Pediatrics 2003;11:1256-1263.

Amin SB, Ahlfors C, Orlando MS, Dalzell LE, Merle KS, Guillet R. Bilirubin and serial auditory brainstem responses in premature infants. Pediatrics 2001; 107: 664-670.

Maisels MJ, Watchko JF. Treatment of jaundice in low birthweight infants. Arch Dis Child Fetal Neonatal Ed 2003; 88: F459-F463.

AAP. Management of hyperbilirubinemia in the newborn infant 35 or more weeks of gestation. Pediatrics 2004; 114: 297-316.

Cashore WJ, Oh W. Unbound bilirubin and kernicterus in low birthweight infants. Pediatrics 1982;69:481-485.

Nakamura H, Yonetani M, Uetani Y et al. Determination of serum unbound bilirubin for prediction of kernicterus in low birth weight infants. Acta Paediatr Jpn 1992;54:642-647.

Ahlfors CE. Criteria for exchange transfusion in jaundiced newborns. Pediatrics 1994;93:488-494.

Shapiro SM. Definition of the clinical spectrum of kernicterus and BIND. J Perinatol 2005;25:54-59.

Blackmon et al. Research on prevention of bilirubin induced brain injury and kernicterus. Pediatrics 2004;114:229-233.

Stoelhorst et al. Developmental outcome at 18 and 24 month of age in very preterm infants. Early Hum Dev 2003;72:83-95

Morris BH et al. Aggressive versus conservative phototherapy for infants with extremely low birth weight. N Engl J Med 2008;359:1885-1896

SUPPLEMENTS

Study guidelines
Patiënteninformatiebrief
Informed consent
Systematic Review

STUDY GUIDELINES

Table 1. Study guideline for use of phototherapy and exchange transfusion in preterm infants based on B:A ratio (μmol/l /g/l) and/or TSB (μmol/l), whichever comes first

Birthweight (g)	Phototherapy				Exchange transfusion			
	standard risk		high risk		standard risk		high risk	
	TSB	B:A	TSB	B:A	TSB	B:A	TSB	B:A
**<1250**	150	6.0	100	4.0	220	8.8	170	6.8
**1250 - 1499**	190	6.3	150	5.0	260	10.4	220	8.8
**1500 - 2000**	220	7.3	190	6.3	290	11.6	260	10.4
**2000 - 2500**	240	8.0	220	7.3	310	12.4	290	11.6

Guideline for the use of phototherapy and exchange transfusion in preterm infants based on TSB and B:A ratio, whichever comes first. TSB in μmol/l and albumin in g/l, B:A ratio in μmol/l/g/l = μmol/g.
Phototherapy is initiated whenever one of the thresholds is reached. Phototherapy is stopped whenever both, B:A ratio and TSB levels are under the thresholds. Exchange transfusion is considered whenever the thresholds are reached despite intensive phototherapy. Phototherapy thresholds are derived from exchange transfusion threshold minus 4 mg/dl = 70 μmol/l.
high risk:
- asfyxia: apgar score < 3 at 5 minutes
- hypoxemia: for more than 2 hours PaO2 < 40 mmHg (< 5.3 kPa) in recent 24 hours
- acidosis: for more than 1 hour pH < 7.15 in recent 24 hours
- birthweight < 1000 gram
- hemolysis: positive coombs reaction
- clinical or central nervous system deterioration: sepsis needing vasopressors, intracranial heamorrhage, meningitis.

based on guidelines provided by Ahlfors 1994 and Maisels 2003

Table 2. Study guideline for use of phototherapy and exchange transfusion in preterm infants based on TSB (μmol/l) only

Birthweight	Phototherapy		Exchange transfusion	
	standard risk	high risk	standard risk	high risk
	TSB	*TSB*	*TSB*	*TSB*
**<1250**	150	100	220	170
**1250 - 1499**	190	150	260	220
**1500 - 2000**	220	190	290	260
**2000 - 2500**	240	220	310	290

Guideline for the use of phototherapy and exchange transfusion in preterm infants based on TSB (in μmol/l).
Phototherapy is initiated whenever the threshold is reached. Phototherapy is stopped whenever TSB is under the threshold. Exchange transfusion is considered whenever the threshold is reached despite intensive phototherapy.
high risk:
- asfyxia: apgar score < 3 at 5 minutes
- hypoxemia: for more than 2 hours PaO2 < 40 mmHg (< 5.3 kPa) in recent 24 hours
- acidosis: for more than 1 hour pH < 7.15 in recent 24 hours
- birthweight < 1000 gram
- hemolysis: positive coombs reaction
- clinical or central nervous system deterioration: sepsis needing vasopressors, intracranial heamorrhage, meningitis.

based on guidelines provided by Ahlfors 1994 and Maisels 2003

PATIENTEN INFORMATIE EN INFORMED CONSENT

ONDERZOEK NAAR HYPERBILIRUBINEMIE BIJ PREMATUREN

Reducing bilirubin induced neurological dysfunction in preterm infants: Additional use of bilirubin:albumin ratio in the treatment of hyperbilirubinemia.

-Het reduceren van bilirubine geïnduceerde neurologische schade bij te vroeg geboren kinderen: additioneel gebruik van de bilirubine: albumine ratio voor de behandeling van hyperbilirubinemie -

INFORMATIE TEN BEHOEVE VAN OUDERS VAN DEELNEMERS

Geachte mevrouw/mijnheer,

Uw kind is in onze kliniek onder behandeling omdat hij/zij te vroeg geboren is. Zoals in alle academische ziekenhuizen wordt ook in het UMC Groningen medisch-wetenschappelijk onderzoek gedaan om de zorg te verbeteren. Dergelijk onderzoek is alleen mogelijk met de medewerking van patiënten. Wij willen u hierbij vragen om toestemming om ook uw kind in een onderzoek te betrekken. In deze brief willen wij u informatie geven over het doel van het onderzoek waarvoor uw kind in aanmerking komt, de onderzoeks-procedure en de voor- en nadelen ervan. Deelname aan het onderzoek is vrijwillig. Als u deze informatie gelezen hebt en hierover nog vragen hebt kunt u die met uw arts bespreken. Als u vindt dat u voldoende informatie hebt kunt u beslissen of u aan het onderzoek wilt deelnemen.

Wetenschappelijk onderzoek

Zoals u waarschijnlijk weet kunnen alle pasgeborenen enkele dagen na de geboorte een gele kleur krijgen. Dit wordt veroorzaakt door een gele kleurstof (bilirubine). Het bilirubine is een afvalstof van de rode bloedcellen. Kort na de geboorte is de lever nog niet rijp genoeg om de gele bilirubine op te ruimen en blijft er dus meer bilirubine in het bloed. Bilirubine kan vanuit het bloed in de huid komen en geeft de huid de gele kleur. Bij kinderen die te vroeg geboren zijn is de aanmaak van bilirubine hoger en is de lever nog minder rijp. Daarom worden te vroeg geboren kinderen nog vaker dan op tijd geboren kinderen geel. Het bloed bevat dan veel bilirubine. Dit heet hyperbilirubinemie. Een te hoog gehalte van bilirubine kan schadelijk zijn voor de hersenen. Wanneer het bilirubine gehalte boven een bepaalde grenswaarde komt, zal een behandeling gestart moeten worden die erop gericht is het bilirubinegehalte te laten dalen. De behandeling bestaat uit lichttherapie (fototherapie), waarbij blauwe licht dat op de huid schijnt, bilirubine afbreekt tot stoffen, die gemakkelijk uit het lichaam verwijderd kunnen worden. Dit duurt enige tijd. Soms is het gehalte bilirubine in het bloed zo hoog dat we daar niet op kunnen wachten en moet het gele bloed gewisseld worden met ander bloed om snel het bilirubine gehalte te laten dalen. Deze wisseltherapie is een meer ingrijpende behandeling die ook tot complicaties kan leiden.
De huidige behandeling is gebaseerd op het totale bilirubinegehalte. Wij willen onderzoeken of de behandeling van hyperbilirubine nog beter kan door de behandeling niet alleen van het bilirubine, maar ook van het albumine eiwit af te laten hangen. In het bloed wordt namelijk het grootste deel van het bilirubine gebonden oftewel vastgehouden door dit eiwit (albumine). Vooral het niet aan albumine gebonden bilirubine (vrije bilirubine) kan vanuit het bloed in de hersenen komen. Het aan albumine gebonden bilirubine kan dat niet. Een laag albumine gehalte kan dus betekenen dat er meer vrij bilirubine is met daardoor een toegenomen kans op hersenschade.
De vraag is of de schadelijke effecten van bilirubine op de ontwikkeling van kinderen kan worden voorkomen door de behandeling te baseren op een combinatie van bilirubine en albumine. Om dit te

bestuderen wordt in ons ziekenhuis een wetenschappelijk onderzoek uitgevoerd. Naast ons ziekenhuis doen ook andere ziekenhuizen in Nederland mee. In totaal zullen ruim 600 kinderen aan het onderzoek mee gaan doen.

Het onderzoek waarvoor uw deelname wordt gevraagd

In het onderzoek wordt bestudeerd of de behandeling van te vroeg geboren babies met een hoog bilirubine gehalte door het meten van het eiwit albumine in het bloed, naast het bilirubine gehalte mogelijke negatieve effecten op de ontwikkeling van de kinderen worden voorkomen. De kinderen die aan het onderzoek meedoen, worden door de computer in twee groepen verdeeld. De ene groep wordt behandeld op de manier zoals we dat gebruikelijk doen, dus behandeling op basis van het totale gehalte van bilirubine in het bloed. Bij de andere groep wordt naast het totale gehalte van bilirubine, het albumine gehalte meegenomen in de beoordeling of behandeling met blauw licht of wisseltherapie gestart moet worden. Op twee jarige leeftijd zal de ontwikkeling van de kinderen getest worden. Door de resultaten onderling te vergelijken kunnen we nagaan wat het beste is.

De gang van zaken tijdens het onderzoek

Bij beide groepen kinderen zullen we de eerste tien levensdagen het bilirubine- en albumine gehalte in het bloed bepalen. Het onderzoek duurt in principe 10 dagen. Als u kind langer geel blijft zal het onderzoek zolang duren totdat die geelheid verdwenen is. Daarna zal op tweejarige leeftijd de ontwikkeling van uw kind worden getest en een uitgebreid lichamelijk onderzoek worden gedaan. Naast de standaard gehoorscreening zal er in de eerste 10 levensdagen een uitgebreider gehoors-onderzoek (afgekort: ABR) plaatsvinden, waarbij geluidsklikjes via een koptelefoontje worden gegeven en via 3 plakelectroden op het hoofd wordt gemeten of het geluid goed aankomt.
De totale duur van het hele onderzoek is 3 jaar. Na die periode vergelijken we de resultaten van alle kinderen die aan het onderzoek hebben meegedaan en kunnen we zien wat de beste manier van behandelen is.

Wat betekent meedoen voor uw kind ?

De eerste tien levensdagen zal er dagelijks bloed worden onderzocht op bilirubine en albumine. Als het bilirubinegehalte te hoog is zal behandeling worden gestart in beide groepen. In de tweede groep wordt ook gekeken naar het albumine gehalte. Mocht dat relatief laag zijn dan zullen we eerder beginnen met de behandeling. De keuze op welke van de twee wijzen uw kind behandeld wordt, wordt door de computer gedaan. U en wij mogen dus niet kiezen, zodat de kinderen zo eerlijk mogelijk over beide groepen verdeeld worden. We mogen u dus ook niet vertellen in welke groep uw kind terrecht is gekomen, zodat zowel u en wij niet bevooroordeeld zijn als de ontwikkelingstests worden gedaan.
Bij alle kinderen die voor de 32^{e} zwangerschapsweek geboren zijn wordt de eerste levensdagen vaak dagelijks bloedonderzoek verricht. Dit is nodig voor de gebruikelijke behandelingen. Meestal zal dan ook het bilirubinegehalte een aantal keren worden bepaald. Voor dit onderzoek zal het bilirubine gehalte dagelijks worden bepaald. Het bepalen van het eiwit albumine is extra. Daarvoor is echter meestal geen extra bloed afname nodig. Restanten van bloedmonsters en urinemonsters zullen bewaard blijven zodat we daarin stoffen (vrij bilirubine en lumirubine) kunnen meten die te maken hebben met de bilirubine stofwisseling.
Er zal als uw kind 18-24 maanden oud is, bij de gebruikelijke polikliniek controle met aandacht voor groei en ontwikkeling van uw kind, een uitgebreide ontwikkelingstest worden gedaan. Ook de artsen en assistenten op de polikliniek weten niet in welke onderzoeksgroep uw kind is geweest, zodat zij niet bevooroordeeld zijn. De polikliniekbezoeken (follow-up) zijn standaard voor alle kinderen die te vroeg

geboren zijn, dus ook voor de kinderen die niet meedoen aan het onderzoek. De ontwikkelingstest is extra in verband met het onderzoek, het gebeurd spelenderwijs en duurt ongeveer 3 uur.
Het is mogelijk dat we op latere leeftijd (4-7 jaar) nogmaals een uitgebreide ontwikkelingstest zouden willen doen, maar dat is nog geen onderdeel van deze studie. Misschien benaderen we u later of u daaraan zou willen meedoen. Wij vragen nu alvast uw toestemming hiervoor.

Bijwerkingen, voor- en nadelen

Voor dit onderzoek hoeft u kind geen extra prikken te ondergaan en meestal hoeft er ook niet meer bloed te worden afgenomen dan voor de gebruikelijke behandeling nodig is. In sommige gevallen moet er wel iets meer bloed worden afgenomen (0.1 ml). Daarnaast is het mogelijk dat uw kind eerder en/of langer blauw lichttherapie krijgt in verband met de hyperbilirubinemie. Uw kind zou mogelijk eerder in aanmerking komen voor wisseltherapie, maar het kan ook zijn dat door het eerder starten van de blauw lichttherapie juist wisseltherapie voorkomen kan worden. Het effect daarop weten we niet goed.

Bedenktijd

Natuurlijk zult u tijd nodig hebben om erover na te denken of u aan dit onderzoek wilt meewerken. Hiervoor krijgt u uiteraard de gelegenheid. Omdat de studie op de eerste levensdag moet beginnen is de maximale bedenktijd 24 uur. Mocht er iets niet geheel duidelijk zijn dan willen wij u dat graag uitleggen.

Vertrouwelijkheid van de gegevens

De gegevens die voor dit onderzoek over uw kind verzameld worden, zullen vertrouwelijk worden behandeld. De gegevens worden op aparte formulieren ingevuld, waarop alleen een nummer voorkomt, niet de naam en persoonlijke gegevens. De gegevens worden dus anoniem verwerkt. In publicaties zal de naam van uw kind niet terug te vinden zijn.
Het is van groot belang dat de resultaten van het onderzoek juist worden weergegeven. Daarom worden al tijdens het onderzoek de gegevens gecontroleerd. Hiervoor zal tijdens het onderzoek de verzamelde gegevens door een onafhankelijke waarnemer worden vergeleken met de gegevens in het medische dossier van uw kind. De leider van het onderzoek is er verantwoordelijk voor dat op vertrouwelijke wijze met die gegevens wordt omgegaan. Tenslotte bestaat er de mogelijkheid dat de Inspectie voor de Volksgezondheid, als officiële instantie, de gang van zaken rond het onderzoek komt inspecteren.

Verzekering

Voor alle kinderen die meedoen aan deze studie is een verzekering afgesloten voor onverwachtse schade door deelname aan dit onderzoek. Het betreft de schade door letsel of overlijden die zich openbaart gedurende de deelname aan dit onderzoek en de schade die zich openbaart binnen vier jaar na beëindiging van deelname aan dit onderzoek. De verzekering is voor had Universitair Medisch Centrum Groningen afgesloten bij Onderlinge Waarborgmaatschappij Centramed B.A., Postbus 191, 2270 AD Voorburg.

Vrijwilligheid van deelname

U bent er geheel vrij in wel of geen toestemming te verlenen voor de deelname aan dit onderzoek. Verder heeft u altijd, ook wanneer u schriftelijk heeft verklaard te willen deelnemen, het recht om zonder opgave

van redenen af te zien van verdere deelname aan het onderzoek. Deze beslissing zal geen nadelige gevolgen hebben op de verdere behandeling en geen invloed hebben op de zorg en aandacht, waarop uw kind in ons ziekenhuis recht heeft. Ook uw behandelend arts kan in het belang van uw kind achten het onderzoek voortijdig te beëindigen. Hij/zij zal dit dan met u bespreken.
Indien u er niets voor voelt om met het onderzoek mee te doen zal de tot nu toe gebruikelijke therapie of handelwijze gevolgd worden, de behandelend arts zal op indicatie het bilirubine gehalte bepalen en niet het albuminegehalte.
Mocht in de periode dat u aan het onderzoek deelneemt nieuwe informatie bekend worden die van invloed kan zijn op uw bereidheid om mee te werken, dan zullen wij u hiervan zo spoedig mogelijk op de hoogte stellen, zodat u uw beslissing kunt heroverwegen.

Nadere informatie

Mocht u na het lezen van de brief, voor of tijdens het onderzoek nog nadere informatie willen ontvangen of komen er nog vragen bij u op dan kunt u altijd contact opnemen met de uitvoerders van het onderzoek, Dr. P.H. Dijk en Dr. C.V. Hulzebos telefonisch te bereiken via 050 -3614215. In spoedeisende gevallen kunt u ons ziekenhuis bellen via het algemene telefoonnummer: 050-3616161.
Indien u nadere informatie wenst onafhankelijk van de uitvoerder van het onderzoek dan kunt u contact opnemen met Dr. M. van Stuijvenberg, kinderarts, telefonisch te bereiken op 050-3614215. Dr. van Stuijvenberg is niet direct bij het onderzoek betrokken.

Ondertekening toestemmingsverklaring

Als u besluit mee te werken aan het onderzoek zullen wij u vragen een formulier te ondertekenen. Door ondertekening van dit formulier ('Toestemmingsverklaring' oftewel 'Informed Consent') stemt u in met deelname aan dit onderzoek. U blijft de vrijheid behouden om wegens voor u relevante redenen uw medewerking te stoppen. De arts of verpleegkundige zal het formulier eveneens ondertekenen en bevestigt dat hij/zij u heeft geïnformeerd over het onderzoek, deze informatiebrief heeft overhandigd en bereid is om waar mogelijk in te gaan op nog opkomende vragen.

Brochure

Over deelname aan wetenschappelijk onderzoek in het algemeen is in ons ziekenhuis een brochure beschikbaar. Als u deze niet tegelijk met deze informatiebrief krijgt uitgereikt kunt u deze op verzoek alsnog krijgen.

Dr. P.H. Dijk, kinderarts-neonatoloog
Dr. C.V. Hulzebos, kinderarts-neonatoloog
Tel 050-3614215 via zoemer 55077 of 55078

BIJLAGE BIJ SCHRIFTELIJKE INFORMATIE
ONDERZOEK NAAR HYPERBILIRUBINEMIE BIJ PREMATUREN

-Het reduceren van bilirubine geïnduceerde neurologische schade bij te vroeg geboren kinderen: additioneel gebruik van de bilirubine: albumine ratio voor de behandeling van hyperbilirubinemie -

INFORMATIE INZAKE DE VERZEKERING

Er is een verzekering afgesloten voor onverwachte schade die u lijdt door uw deelname aan dit wetenschappelijk onderzoek. Het betreft de schade door letsel of overlijden die zich openbaart gedurende de deelname aan dit onderzoek en de schade die zich openbaart binnen vier jaar na beëindiging van deelname aan dit onderzoek.

Het bedrag waarvoor de verzekering is afgesloten is maximaal € 5.000.000,- voor de totale schade die zich per verzekeringsjaar bij proefpersonen heeft geopenbaard bij alle onderzoek dat door het Universitair Medisch Centrum Groningen en Rijksuniversiteit Groningen wordt verricht, maximaal € 3.500.000,- voor de totale schade bij dit onderzoek en maximaal € 450.000,- per proefpersoon.

Bepaalde soorten van schade kennen wettelijk gelimiteerde vergoedingen.
Van de dekking door de verzekering is uitgesloten:

- schade van te verwachten risico's zoals beschreven in de schriftelijke informatie voor proefpersonen, tenzij deze ernstiger zijn dan beschreven;
- bij deelname door patiënten: schade door verslechtering van de gezondheid of het uitblijven van de verbetering van de gezondheid;
- schade waarvan (nagenoeg) zeker is dat deze zich bij de proefpersoon zal voordoen;
- schade door aantasting van de gezondheid van de proefpersoon die zich ook geopenbaard zou hebben wanneer de proefpersoon niet aan dit onderzoek had deelgenomen;
- schade die zich bij nakomelingen openbaart als gevolg van een nadelige inwerking van het onderzoek op het genetisch materiaal van de proefpersoon;

De verzekering is afgesloten bij Onderlinge Waarborgmaatschappij Centramed, Postbus 191, 2270 AD VOORBURG onder polisnummer 624.529.102.

Indien u schade heeft geleden of het vermoeden daarvan heeft, dient u zich direct met Dr P.H. Dijk of Dr. C.V. Hulzebos (leden van het onderzoeksteam) telefoonnummer 050-3614215 in verbinding te stellen en de aanwijzingen op te volgen.
Ook kunt u in zo'n geval contact op te nemen met de juridisch stafmedewerker van het Universitair Medisch Centrum Groningen bereikbaar via telefoonnummer 050-3614929 of 050-3614304.

ONDERZOEK NAAR HYPERBILIRUBINEMIE BIJ PREMATUREN

-Het reduceren van bilirubine geïnduceerde neurologische schade bij te vroeg geboren kinderen: additioneel gebruik van de bilirubine: albumine ratio voor de behandeling van hyperbilirubinemie -

TOESTEMMINGSVERKLARING/ INFORMED CONSENT

Wij zijn gevraagd om toestemming te verlenen voor deelname aan bovenvermeld wetenschappelijk onderzoek van:

Naam en voorletters van patiënt/deelnemer: ……………………………………………………

Geboortedatum van de patiënt/deelnemer: ………………………………………………..

Wij zijn naar tevredenheid over het onderzoek geïnformeerd. Wij hebben de schriftelijke informatie goed gelezen. Wij zijn in de gelegenheid gesteld om vragen over het onderzoek te stellen. Onze vragen zijn naar tevredenheid beantwoord. Wij hebben goed over deelname aan het onderzoek kunnen nadenken. Wij hebben het recht onze toestemming op ieder moment weer in te trekken zonder dat wij daarvoor een reden behoeven op te geven.

Wij stemmen toe met deelname van bovenvermelde persoon aan het onderzoek.

Wij willen in de toekomst wel benaderd worden voor eventueel vervolgonderzoek.

Naam en voorletters van de ouder 1: …………………………………………………………

Relatie tot patiënt-deelnemer: ……………………………………………………………….

Handtekening: ……………………………………………**Datum:**…………………………

Naam en voorletters van ouder 2: ……………………………………………………………

Relatie tot patiënt-deelnemer: ……………………………………………………………….

Handtekening: …………………………………………… **Datum:** ………………………….

Ondergetekende verklaart dat de hierboven genoemde personen zowel schriftelijk als mondeling over het bovengemelde onderzoek geïnformeerd zijn. Hij/zij verklaart tevens dat een voortijdige beëindiging van de deelname door bovengenoemde persoon van geen enkele invloed zal zijn of de zorg die hem of haar toekomt.

Naam arts/verpleegkundige: …………………………………………………………………

Functie: ……………………………………………………………………………………...

Handtekening: …………………………………………… **Datum:**…………………..........

SYSTEMATIC REVIEW

Reducing bilirubin-induced neurological dysfunction in premature newborns: additional use of bilirubin:albumin ratio in the treatment of hyperbilirubinemia.

This record should be cited as: Dijk PH, Hulzebos CV. A systematic review upon the bilirubin:albumin ratio in the treatment of hyperbilirubinemia in preterm newborns.

Background

Neonatal jaundice due to unconjugated hyperbilirubinemia occurs in almost all preterm infants and is potentially harmful for the central nervous system. Treatment is based on total serum bilirubin (TSB) concentration, but is not evidence based. The risk of bilirubin-induced neurological dysfunction including neurodevelopmental delay (BIND) is in part determined by the total bilirubin pool, but also by the ability of non-albumin bound free bilirubin to enter the brain. Therefore, BIND may depend also on the mutual relation between bilirubin and albumin. The bilirubin:albumin (B:A) ratio is an interesting additional measurement in the management of hyperbilirubinemia.

Objectives

To determine the efficacy of the additional use of the B:A ratio in the management of hyperbilirubinemia, reducing the incidence of BIND including neurodevelopmental delay in preterm infants with unconjugated hyperbilirubinemia when compared to TSB concentrations only.

Search strategy

We searched National library of medicine (Medline): http://www.pubmed.nl, Cochrane Library: http://www.nelh.nhs.uk/cochrane.asp, including Cochrane Database of Systematic Reviews (CDSR), Database of Abstracts on Reviews and Effectiveness (DARE), Cochrane Controlled Trial Register (CCTR), Current Controlled Trials (CCT): http://controlled-trials.com/, ClinicalTrials.gov: http://clinicaltrials.gov/, NHS Centre for Reviews and Dissemination (CRD): http://www.york.ac.uk/inst/crd/crddatabases.htm.
We used the relevant MESH and Text search terms: “hyperbilirubinemia”, “bilirubin”, “jaundice, neonatal” or “kernicterus” in combination with “infant, premature” or “infant, low birth weight” in combination with “serum albumin” or “bilirubin:albumin ratio” or “B:A ratio” in combination with “BIND”, “neurodevelopmental outcome” or “neurotoxicity”. We hand-searched the articles cited in each publication obtained. Two authors extracted data independently.

Selection criteria

We included studies, in which preterm neonates born at gestational age less than 32 weeks (age 28 days of life or less) with unconjugated hyperbilirubinemia due to any cause were analyzed with reference to the association between TSB concentration and and/or B:A ratio, and neurodevelopmental outcome or neurotoxicity. We excluded studies in term and near-term infants.

Results

Initially, we found no references following the search combination “hyperbilirubinemia” and “infant, premature” and “serum albumin” or “B:A ratio” and “neurodevelopmental outcome” or “outcome”.
We found and read 21 papers following the search combination “hyperbilirubinemia” or “kernicterus”and “infant, premature” and “serum albumin”.
We found 293 references following the search “hyperbilirubinemia” or “kernicterus” and “infant, premature”. These abstracts of these references were screened for relevance.
We found 169 references following the search “hyperbilirubinemia” or “kernicterus” and “serum albumin”. The abstracts of these references were screened for relevance.
The majority of the abstracts and papers that have been screened did not contain any outcome measure related to neurotoxicity or neurodevelopmental outcome, others did not contain albumin or B:A ratio measurement. All papers with a combination of bilirubin or hyperbilirubinemia, albumin or B:A ratio, prematurity and a outcome related to neurotoxicity (including kernicterus) were included. No additional methodological exclusion criteria were applied due to the small number of relevant papers left.

We found no ongoing clinical trials that evaluate the efficacy of B:A ratio versus TSB in preterm infants with hyperbilirubinemia. We found no systematic reviews or prospective clinical trials evaluating long term neurodevelopmental consequences of using the B:A ratio (with or without the additional use of TSB) versus TSB only in the management of preterm infants with hyperbilirubinemia. One prospective cohort study evaluated short term effects of B:A ratio’s in predicting bilirubin encephalopathy (Amin 2001). One studie evaluated retrospecively data of a prospective randomised controlled NICHD phototherapy trial, and studied the association between neurodevelopmental outcome and the B:A ratio (NICHD 1985, Scheidt 1990 and Scheidt 1991). One prospective and two retrospective case-control post-mortum studies analysed the relation between the B:A ratio and kernicterus in preterm infants (Ritter 1982, Kim 1980, Cashore 1982). One patient series reported MRI documented kernicterus and its relation to B:A ratio’s (Govaert 2003). Several reviews discussed the use of the B:A ratio in the management of hyperbilirubinemia in preterm infants (Ahlfors 1994, Maisels 2003, Watchko 2003, Bhutani 2004 2x, Kaplan 2005).

Amin et al studied in a single center prospective cohort study the usefulness of the B:A ratio and unbound bilirubin as compared with TSB in predicting bilirubin encephalopathy assessed by serial auditory brainstem responses (ABR) in 143 infants of 28-32 weeks of gestational age (Amin 2001). The mean peak TSB concentration in infants with normal ABR maturation was not significantly different from the mean peak TSB in infants with abnormal maturation.

In the total group there was a trend that B:A ratio was better than TSB in predicting abnormal ABR maturation. In the subset of 45 infants in whom unbound bilirubin was measured, although TSB was not different, there was a significant difference in B:A ratio between the infants with normal versus abnormal ABR maturation. Unbound bilirubin showed to be the most sensitive predictor of abnormal ABR maturation, and hence transient bilirubin encephalopathy in premature newborns with hyperbilirubinemia. The authors support at least to consider the B:A ratio along with the TSB in the management of hyperbilirubinemia in preterm infants.

The National Institute of Child Health and Human Development Cooperative Phototherapy Study was performed in the US between 1974 and 1976 (NICHD 1995, Scheidt 1990 and 1991). This randomised controlled study at six centers comparing the effect of phototherapy versus no-phototherapy for the treatment of hyperbilirubinemia on neurodevelopmental outcome in 1339 infants, included a cohort of low birth weight infants < 2000 gram. It was shown that phototherapy effectively controlled neonatal hyperbilirubinemia without evidence of adverse outcome at 6 years of age.

Scheidt et al analysed a subgroup of 224 preterm / low birth weight infants (< 2000 gram) that were randomised to the control group. The control group did not receive phototherapy and TSB levels were maintained below specified levels by the use of exchange transfusions. In this subgroup the relationship between TSB levels and neurodevelopmental outcome was evaluated. There was no relation between TSB levels and cerebral palsy, and IQ was not associated with TSB levels, time and duration of exposure to bilirubin. The B:A ratio had a near-signficant (p=0.06), though weak, inverse relation with IQ.

Kim et al. reviewed 398 neonatal autopsies in preterm infants (Kim1980). The 27 infants with kernicterus (prevalence of 7%) had relatively low mean TSB peak concentrations, which were similar to those in a restrospective contol group of 103 infants without kernicterus. Serum albumin values and bilirubin binding capacity were significantly lower in kernicteric infants compared to controls. B:A ratios were calculated in six of the 27 kernicteric infants and 15 of the 103 non-kernicteric infants. B:A ratios were similar in both groups.

Cashore et al. determined TSB, free unbound bilirubin, B:A ratio and bilirubin binding affinity in 13 preterm infants < 1500 gram with hyperbilirubinemia before exchange transfusions were performed and later died (Cashore 1982). Five of these 13 infants had kernicterus at autopsy, eight had not. Compared to non-kernicteric infants unbound bilirubin levels were increased and bilirubin binding affinity was decreased in infants with kernicterus, in association with low B:A ratios in the latter.

Ritter et al. prospectively assessed risk factors in the development of kernicterus in 91 infants < 1500 g. (Ritter 1982). In 30 of the 53 infants that died, autopsie was performed. Seven had kernicterus and 23 not. There was no statistically significant difference between infants with and without kernicterus in peak TSB concentration (mean and SEM: 7.3 ±1.3 vs 6.1 ±0. 5 mg/dl), free bilirubin (18.2 ± 4.5 vs 11.1 ± 0.9 nm/l) or albumin (2.8 ±0.2 vs 2.8 ±0.1 g/dl). Although not explicity provided in their paper, B:A ratios also appear to be not statistically different between infants with and without kernicterus (calculated group mean values 2.6 vs 2.2 mg/g).

Govaert et al. reported five preterm infants with clinical signs of kernicterus and related changes in globus pallidus on magnetic resonance imaging (MRI) and/or sonography with TSB levels below currently used exchange transfusion thresholds, but with high B:A ratios (Govaert 2003). In three infants, a combined respiratory and metabolic acidosis had been present around the peak TSB concentration. ABR's were severely impaired in all

preterm infants with elevated B:A ratios and all developed hearing loss facing “accepted” TSB levels. The authors conclude that the pathophysiological role of low serum albumin levels must be considered in BIND especially in acidotic jaundiced preterm infants.

Ahlfors investigated in 54 infants (35 term and 19 preterm) whether the B:A ratio is a reliable predictor of bilirubin-albumin binding by mathematical analyses of the relationship of the B:A ratio to the unbound bilirubin concentration in jaundiced newborns. He found the B:A ratio to be an reliable surrogate for the unbound bilirubin concentration (Alhfors 1994). He concluded that the B:A ratio is a simple nonambiguous way of incorporating the serum albumin concentration into exchange transfusion criteria.

Watchko and Maisels reviewed the pathophysiology, neurodevelopmental outcome and treatment of jaundice in low birth weight infants (Watchko 2003 and Maisels 2003). The authors discussed the lack of legitimate evidence in the treatment of hyperbilirubinemia in preterm infants and summarized the role of albumin binding and B:A ratio. Although no contemporay long term studies relating unbound bilirubin or B:A ratio to developmental outcome are available, the authors support the use of the B:A ratio together with, but not in lieu of, the TSB level as an additional factor in determining the need for exchange transfusion.

Bhutani et al. described six case studies of preterm infants who developed kernicterus (Bhutani 2004). They conclude that the remergence of kernicterus in preterm infants is a matter of concern. They suggest that in the absence of commercial assays for unbound bilirubin or albumin binding reserve, in the mean time, the B:A ratio offers the clinician a reasonable measure of bilirubin binding to albumin in the management of hyperbilirubinemia.

Bhutani and Johnson discussed the need for accurate and precise bilirubin measurements to prevent kernicterus and BIND (Bhutani 2004). The care of the vulnerable sick and preterm infant could be optimized by better techniques to assess the neurotoxic potential of bilirubin. The authors express that measures of bilirubin-binding reserve to albumin and the B:A ratio can be used in the evaluation of preterm infants with hyperbilirubinemia.

Discussion

We found in our review of the literature no evidence from prospective clinical trials that the use of B:A ratios in the management of hyperbilirubinemia reduces long term bilirubin related neurotoxicity. However, it must be recognised that circumstantial evidence illustrates that the additional use of the B:A ratio in the management of hyperbilirubinemia may have beneficial effects in the prevention of BIND in preterm infants.

Recently, several reviews have discussed the role of the bilirubin-albumin binding in the pathophysiology of bilirubin induced neurotoxicity in preterm infants (Ahlfors 2004, Maisels 2003, Kaplan 2005).

Jaundice in newborns result from an increased bilirubin pool due to increased bilirubin production in relation to a relatively low bilirubin elimination. When the bilirubin load exceed the binding capacity of albumin, free bilirubin levels rise. The free bilirubin portion is related to the TSB, and inversely proportional to albumin and its intrinsic ability to bind bilirubine (K). Therefore, the free bilirubin level helps interpret the risk of bilirubin toxicity at a given TSB. Free bilirubin can cross the blood brain barrier and enter the brain. In vitro - and animal studies have shown the

neurotoxic potential of free unconjugated bilirubin. These studies have recently been reviewed by Ostrow 2003 and Tiribelli 2005.
Preterm infants are more prone to bilirubin neurotoxity because of increased bilirubin load, slower elimination and higher susceptibility. Hyperbilirubinemia in preterm infants is more pronounced due to a higher bilirubin production and lower bilirubin exretion, as a result of immaturity of neonatal red cells, hepatic enzymatic conjugation system, hepatobiliary extrection and gastrointestinal sytem. Furthermore, the potential neurotoxic effects of hyperbilirubinemia is increased in preterm infants due to higher accessability of bilirubin to the brain and more pronounced susceptability of the preterm brain to bilirubin. Nevertheless, several neurodevelopmental follow up studies have shown conflicting results regarding the association between peak TSB levels and later adverse outcome in preterm infants (Watchko 2003). Furthermore, preterm infants have lower serum albumin levels. Therefore, bilirubin binding capacity will be decreased and free bilirubin levels increased. Free bilirubin levels are more closely related to short term (ABR maturation abnormalities) and long term outcomes (kernicterus) than TSB (Amin 2001 and Cashore 1980). Nevertheless, there are no studies evaluating the effect of using free bilirubin measurements in the management of hyperbilirubinemia. The lack of these studies is due to the fact that routine clinical labatory measurements of free bilirubin are not generally available. Therefore, the bilirubin:albumin ratio has been used as an surrogate.

Ahlfors (1994) showed that the B:A ratio correlates with measured free bilirubin levels in newborns, indicating that B:A ratio's might be helpfull in predicting neurotoxicity. In retrospective blood sample analyses of preterm infants who died with or without kernicterus, free bilirubin levels and B:A ratio's tend to have a better correlation with kernicterus than the TSB level did (Kim 1980 and Cashore 1982).
Technological advances in research tools such as magnetic resonance imaging (MRI) and auditory brainstem evoked response (ABR) have been used for objective assessments of bilirubin induced neurotoxicity (Shapiro 2003). Govaert showed that typical subtle MRI abnormalties in preterm infants with clinical signs of kernicterus were related to high B:A ratio;s, but not with peak TSB levels (Govaert 2003). The ABR is an objective tool in the evaluation of bilirubin toxicity in term and preterm infants, as has been recently reviewed by Amin 2004 and Shapiro 2005. Amin et al 2001 showed in a prospective cohort study in preterm infants that free bilirubine and the B:A ratio are more closely related than TSB with transient ABR abnormalities. Although this study was not an interventional trial, it suggests that bilirubin is potentially neurotoxic at lower levels in preterm infants, that this effect can be transient, and that B:A ratio's (after free bilirubin) could better than TSB in monitoring preterm infants in the prevention of potential bilirubin toxicity. This study did not assess neurodevelopmental follow-up, yet.
Scheidt et al did study the association between B:A ratio and neurodevelopmental follow up. In their retrospective analyses of the control subgroup of preterm infants who did not receive phototherapy in the NICHD phototherapy study, a near-significant correlation between B:A ratio and neurodevelopmental outcome at 6 years of age was found.
Despite the lack of evidence from randomised controlled trial several experts in the field of bilirubin research and advisory committee's advocate the use of B:A ratio's in term infants with high bilirubin levels close to the exchange

criteria or in the treatment of hyperbilirubinemia in preterm infants (Ahlfors 1994, Maisels 2003, Watchko 2003, Blackmon 2003, Bhutani 2004 2x).

Conclusions

There is no evidence from prospective clinical trials that the use of B:A ratio's in the management of hyperbilirubinemia reduces BIND including neurodevelopmental delay. However, there is circumstantial evidence that the additional use of B:A ratio may be able to prevent BIND in preterm infants. Therefore, along with the opinion of several experts in the field of bilirubin research the reviewers conclude that the additional use of the B:A ratio may have benificial effects in the prevention of BIND in the management of hyperbilirubinemia in preterm infants. Further prospective clinical studies are needed to elucidate this potential effect.

References

Amin et al. Pediatrics 2001;107:664-670. Bilirubin and serial auditory brainstem responses in premature infants.
National Institute of Child Health and Human Development. Pediatrics 1985;75(suppl):385-441. Randmomized controlled trial of phototherapy for neonatal hyperbilirubinemia.
Scheidt et al. Pediatrics 1990;85:455-463. Phototherapy for neonatal hyperbilirubinemia: six year follow up of the national institute of child health and human developmetnt clinical trial.
Scheidt et al. Pediatrics 1991:87:797-805. Intelligence at six years in relation to neonatal bilirubin level: follow up of the national institute of child health and human development clinical trial of phototherapy.
Kim et al. Pediatrics 1980:66:852-858. Lack in predictive indices in kernicterus.
Cashore et al. Pediatrics 1982;69:418-485. Unbound bilirubin and kernicterus in low birth weight infants.
Ritter et al. Pediatrics 1982;69:260-266. A prospective study of free bilirubin and other risk factors in the development of kernicterus in preterm infants.
Govaert et al. Pediatrics 2003;112:1256-1263. Changes in globus pallidus with (pre)term kernicterus.
Ahlfors. Pediatrics 1994;93:488-494. Criteria for exchange transfusion in jaundiced newborns.
Watchko et al. Arch Dis Child Fetal Neonatal Ed 2003:88:F455-F458. Jaundice in low birth weight infants: pathobiology and outcome.
Maisels et al. Arch Dis Child Fetal Neonatal Ed 2003:88:F459-F463. Treatment of jaundice in low birthweight infants.
Bhutani et al. Sem in Perinatol 2004;28:319-325. Kernicterus in sick and preterm infants (1999-2002): a need for an effective preventive approach.
Bhutani et al. Clinical Chemistry 2004;50:477-480. Urgent clinical need for accurate and precise bilirubin measurements in the united states to prevent kernicterus.
Ahlfors et al. Sem in Perinatol. 2004;28:334-339. Bilirubin-albumin binding and neonatal jaundice.
Tiribelli et al. J of Hepatology. 2005;43:156-166. The molecular basis of bilirubin encephalopathy and toxicity.
Kaplan et al. Clin Chem Acta 2005;356:9-21. Understanding severe hyperbilirubinemia and preventing kernicterus.

Shapiro. Pediatr Neurol 2003:29:410-421. Bilirubin toxicity in the developing nervous system.
Amin et al. Sem in Perinatol 2004;28:340-347. Clinical assessment of bilirubin induced neurotoxicity in premature infants.
Shapiro. J of Perinatol. 2005:25:54-59. Definition of the clinical spectrum of kernicterus and bilirubin induced neurological dysfunction.
Blackmon et al. Pediatrics 2004;114:229-233. Research on prevention of bilirubin induced brain injury and kernicterus: NICHD conference executive summary.
